# On-demand growth of semiconductor heterostructures guided by physics-informed machine learning

**DOI:** 10.1126/sciadv.aeb8867

**Published:** 2026-07-15

**Authors:** Chao Shen, Yuan Li, Wenkang Zhan, Shujie Pan, Fuxin Lin, Kaiyao Xin, Hui Cong, Chi Xu, Xiaotian Cheng, Ruixiang Liu, Zhibo Ni, Chaoyuan Jin, Bo Xu, Siming Chen, Zhongming Wei, Chunlai Xue, Zhanguo Wang, Chao Zhao

**Affiliations:** ^1^ State Key Laboratory of Optoelectronic Materials and Devices, Institute of Semiconductors, Chinese Academy of Sciences, Beijing 100083, China.; ^2^ Center of Materials Science and Optoelectronics Engineering, University of Chinese Academy of Sciences, Beijing 100049, China.; ^3^ Laboratory of Solid State Optoelectronics Information Technology, Institute of Semiconductors, Chinese Academy of Sciences, Beijing 100083, China.; ^4^ HS Photonics Co., Ltd., Xiangjiang Science & Technology Base, Changsha, Hunan 413000, China.; ^5^ State Key Laboratory of Semiconductor Physics and Chip Technologies, Institute of Semiconductors, Chinese Academy of Sciences, Beijing 100083, China.; ^6^ College of Information Science and Electronic Engineering, State Key Laboratory of Silicon and Advanced Semiconductor Materials, Zhejiang University, Hangzhou 310027, China.

## Abstract

Developing tailored heterostructures on demand is essential to meet the growing needs of semiconductor devices. However, traditional methods remain constrained by simulation-based design and iterative trial-and-error optimization. Here, we introduce SemiEpi, a self-driving platform designed for molecular beam epitaxy (MBE) that enables multi-step semiconductor heterostructure growth through in situ Reflection High Energy Electron Diffraction monitoring and on-the-fly feedback control. By integrating MBE reactors, physics-informed machine learning (ML) models, and parameter initialization, SemiEpi designs heterostructures, identifies optimal initial conditions, and proposes experiments for material growth. As a demonstration, we optimized high-density InAs quantum dot growth with a target emission wavelength of 1240 nm, achieving a density of 5 × 10^10^ cm^−2^, a 1.6-fold increase in photoluminescence intensity and a reduced full width at half maximum of 29.13 meV through feedback control of growth temperatures. We further demonstrate SemiEpi’s versatility across different MBE reactors, highlighting its potential to address challenges in multi-step heterostructure growth, enable hardware-independent frameworks, and enhance process repeatability and stability.

## Teaser

Physics-informed machine learning guides on-demand semiconductor heterostructure QD growth via in situ RHEED and feedback control.

## INTRODUCTION

Semiconductor heterostructures are fundamental building blocks in modern electronic and optoelectronic devices. Through precise epitaxial growth, these heterostructures can be customized to achieve desired characteristics such as emission wavelength, carrier mobility, and reflectivity to meet diverse application requirements. For instance, laser diodes require meticulously engineered heterostructures that effectively confine both carriers and photons, ensuring stable emission at target wavelengths (*1*). In contrast, high-electron-mobility transistors incorporate heterojunctions as the channel for electrons to enhance device performance (*2*). However, the development of such heterostructures involves a complex multi-step growth process requiring careful optimization of numerous parameters, with each epitaxial layer demanding specific customization. Conventionally, this process has been heavily reliant on grower expertise and historical data to set parameters, resulting in fixed, open-loop recipes that require trial and error (*3*). Transitioning from material discovery to practical implementation can span considerable periods. As functional demands increase, there is an urgent need for fast, on-demand development of customized semiconductor heterostructures. Achieving such precision has proven to be a substantial challenge for many years due to its complex nature.

In recent years, self-driving experiments guided by machine learning (ML) have shown potential to transform research by streamlining traditionally repetitive tasks with minimal human involvement (*4*). This approach has been successfully employed in small-molecule synthesis, photocatalytic reactions, and the discovery of organic lasers (*5*). Moreover, ML has proven particularly effective in uncovering relationships between dynamic growth processes and final material properties, facilitating the optimization of growth parameters (*6*). However, it faces two key limitations. First, it relies heavily on historical growth datasets, which limits its adaptability to unexplored growth scenarios. Second, the inherent “black-box” and data-hungry nature of ML models obscure interpretability. Integrating physical principles into ML frameworks, termed physics-informed ML (PIML), could enhance both generalization and model transparency, yet this remains a persistent challenge (*7*).

Furthermore, semiconductor heterostructures present multi-faceted challenges due to their inherent complexity, which involves designing the structure through simulation and optimizing multiple interdependent variables, such as growth temperature, flux, and growth rate. Among heterostructures, quantum dot (QD)-based heterostructures are at the forefront of semiconductor research, driven by the growing demand for high-performance and miniaturized devices (*8*). However, developing QD heterostructures introduces additional variables, including precise control over QD size, shape, and density to meet device requirements (*9*). Moreover, dynamic fluctuations in reactor conditions and batch-to-batch variations in sample holders and substrates can render predetermined growth parameters and programs ineffective (*10*). To address these issues, it is essential to establish a self-consistent parameter initialization protocol grounded in the material’s intrinsic properties, such as surface reconstruction transition temperatures, rather than relying solely on historical growth data.

Among growth parameters, temperature stands out as both easily measurable and critically important for QD growth (*11*). Commonly used in situ temperature metrologies—including thermocouples, pyrometers, bandage thermometry, and ellipsometry—are often affected by reactor-specific configurations (*12*–*14*). Calibrating them against intrinsic physical transitions is thus key to deriving an inferred temperature that ensures repeatability across different reactors. Reconstruction transitions, typically monitored in situ via reflective high-energy electron diffraction (RHEED), can vary based on the material’s inherent physical properties. The characteristic temperatures associated with RHEED transitions can help identify optimal growth conditions (*15*). In this context, we reported the growth of QDs and lasers using ML and in situ feedback control assisted by RHEED (*16*–*18*). However, initial conditions still require human intervention, and methods developed for one molecular beam epitaxy (MBE) reactor may not apply to another, which limits the accessibility of customized heterostructures. A robust platform is essential to address these challenges by integrating PIML models (*19*), multi-step processes and enabling real-time optimization. It should prioritize performance-driven heterostructure design, ensure proper parameter initialization, develop predictive models of growth outcomes, and dynamically optimize growth parameters throughout the growth process.

In our work, we introduce SemiEpi, a self-driving platform designed to perform multi-step MBE growth of heterostructures under optimal conditions. It integrates three key innovations: (i) PIML models for QD design, (ii) a self-driving method for multi-step heterostructure growth with parameter initialization, and (iii) in situ monitoring with feedback control for adjusting growth temperatures. Using InAs QDs as a demonstrative heterostructure, SemiEpi employs PIML models to determine the QD size and density based on the desired emission wavelength. The core functionality of SemiEpi involves in situ RHEED videos analysis to monitor surface reconstruction and QD growth. It generates a parameter initialization curve, selects a proper initial growth temperature to ease the burden on the ML models, and fine-tunes the substrate temperature during growth using ML without human intervention. This capability enables the customization of growth conditions for individual heterostructures, marking a notable milestone in establishing a precise growth control scheme and closed-loop experimentation strategies. This way, we can intelligently optimize multi-step growth within a multi-dimensional parameter space, achieving desired material characteristics previously only attainable through time-consuming, labor-intensive experimentation and human intervention. Our platform represents an important step toward the realization of self-driving semiconductor epitaxy, combining physical insights with adaptive ML for unprecedented control over nanoscale material synthesis.

## RESULTS

### Design of SemiEpi

Recent advances in self-driving material synthesis systems have demonstrated the potential to achieve on-demand material properties by leveraging historical experimental data to iteratively optimize growth parameters (*20*–*23*). A critical yet often overlooked aspect of these systems is proper parameter initialization to minimize deviations during growth and reduce the need for adjustments, improving universality across different systems to achieve optimal outcomes. Although recent studies have proposed theoretical frameworks for parameter initialization and growth outcome prediction using in situ data, these methodologies have yet to be implemented or integrated into dynamic control frameworks (*24*, *25*).

**Fig. 1.**
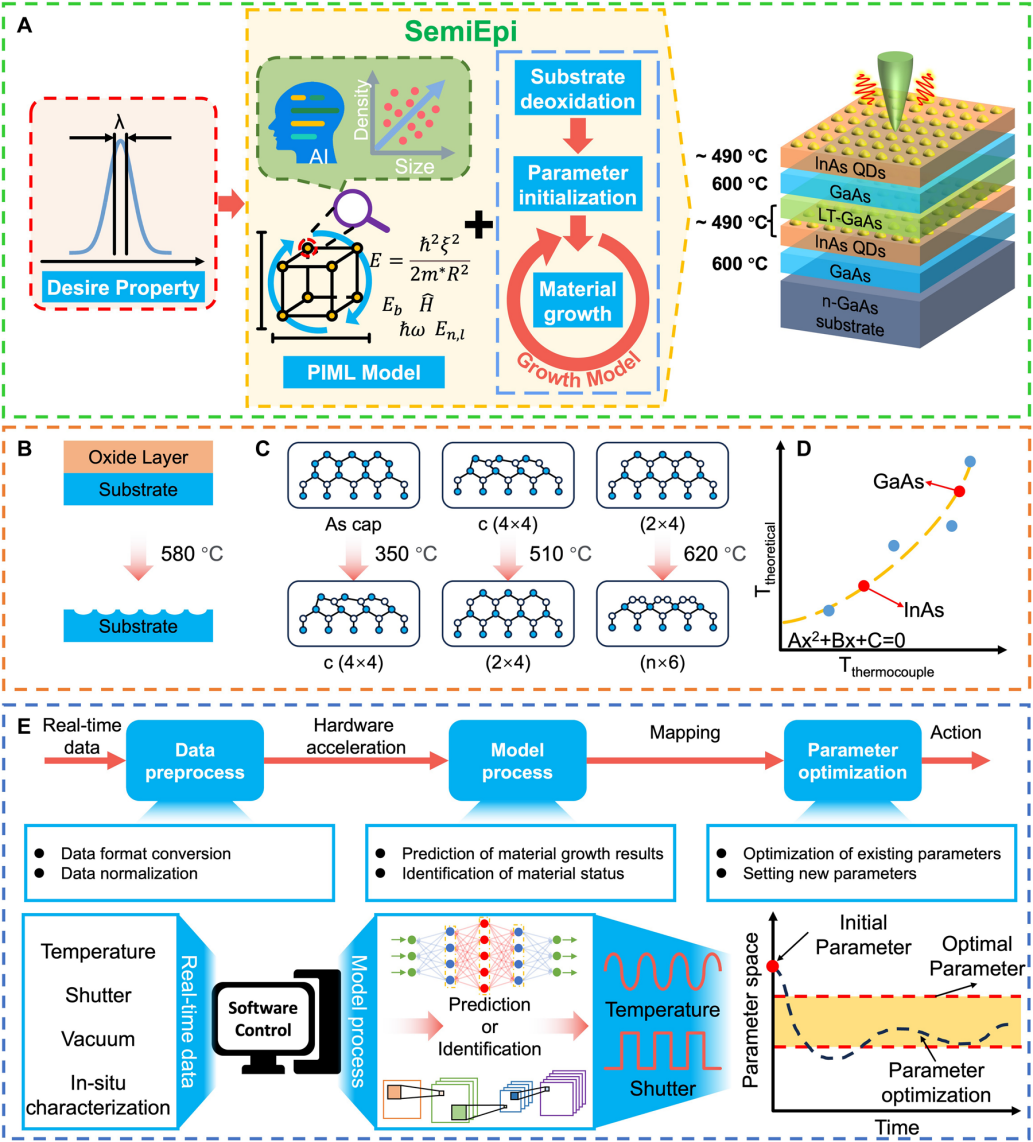
Overview of SemiEpi. (**A**) SemiEpi’s framework. Before SemiEpi, growers defined target properties. SemiEpi then employs a multi-module, sequential workflow to design and grow samples with specified structures. Schematic of (**B**) substrate deoxidation, (**C**) surface reconstruction, (**D**) parameter initialization, and (**E**) material growth.

SemiEpi is designed to address the challenge of optimizing complex, multi-step growth processes for demanded material properties. It integrates PIML models that combine physical constraints to ensure robust interpretability while allowing exploration of parameter spaces associated with QD sizes corresponding to specific emission wavelengths (see Fig. 1A). PIML further leverages ML to uncover complex, nonlinear relationships between growth outcomes. While the emission wavelength of QDs follows established quantum confinement models that relate it to their size, their density and size exhibit a characteristic inverse proportion at fixed deposition amounts. Our framework correlates RHEED signatures with ex-situ morphology and decouples this complexity: physical computation maps the wavelength to QD size, while ML captures the nonlinear relationship between size and density. This synergistic strategy is validated by broader experimental observations of QD evolution under fixed deposition amounts, confirming the reliability of RHEED for real-time morphology prediction (*26*–*29*). This approach opens up opportunities to control materials properties during growth. Once the desired QD size linked to the target emission wavelength is identified, PIML models can predict the corresponding QD density. This methodology transforms complex growth characteristics, often challenging to observe directly, into accessible and measurable parameters. Importantly, the target window is not arbitrarily defined for evaluation but is derived from physics-informed predictions linking QD density and emission wavelength. The task of the system is therefore to realize a predefined physical objective under realistic growth constraints, rather than optimizing toward a self-defined metric.

SemiEpi’s heterostructure growth process is governed by three sequential operational modules: (i) substrate deoxidation, (ii) parameter initialization, and (iii) material growth based on in situ characterization results and ML models called “Initialization Model” and “Growth Models”. It facilitates continuous in situ monitoring, self-optimization, and on-the-fly feedback control to grow specific structures. The process initiates with the substrate deoxidation module, which performs critical surface preparation by thermally removing native oxides to ensure a fresh growth front. During this phase, the substrate temperature gradually increased until the deoxidation feature is observed at ~580°C through in situ RHEED, and the deoxidation temperature is subsequently recorded (see Fig. 1B) (*30*).

The parameter initialization module employs RHEED to monitor real-time surface reconstruction states using the Initialization Model while simultaneously recording the corresponding thermocouple temperatures. During substrate temperature modulation, surface energy and stress variations drive atomic rearrangement, enabling observation of distinct reconstruction states (*31*). Each state transition occurs at a characteristic temperature range. For example, the As cap desorbs from the GaAs surface within a range of 300–350°C as surface energy decreases with increasing temperature (*32*–*35*). Further heating induces sequential reconstruction transitions from c(4 × 4) to (2 × 4) at 480–550°C, followed by (2 × 4) to (n × 6) at 580–620°C (see Fig. 1C) (*31*, *36*, *37*). By correlating measured thermocouple readings with these theoretically established transition temperatures, SemiEpi constructs a robust parameter initialization curve (see Fig. 1D). Finally, we determine the theoretical temperatures for these transitions as 350°C, 510°C, and 620°C, respectively. While these benchmarks are selected from established ranges, this choice does not compromise growth outcomes. This enables precise alignment of actual growth temperatures with inferred optima. In this way, SemiEpi provides inherent adaptability across different MBE systems, overcoming the limitations of indirect measurement and minimizing the need for reactor-specific parameter adjustments in subsequent processes. Beyond parameter initialization results, SemiEpi will subsequently integrate Growth Models and real-time feedback control to fine-tune the substrate temperature based on in situ RHEED data dynamically. Initialization and real-time control play distinct roles in SemiEpi. Initialization provides a physically consistent starting point by calibrating reactor-specific parameters based on intrinsic material transitions. However, it does not determine the final outcome.

The material growth module functions as the closed-loop control core for heterostructure growth. It starts by continuously acquiring multifaceted real-time data—including temperature, shutter status, and in situ characterization inputs—for a comprehensive view of the growth environment (see Fig. 1E). The raw data is preprocessed (converted and normalized) for numerical stability and model compatibility, then fed into Growth Models selected by data type. To meet the strict latency requirements of precision epitaxy, the models are hardware-accelerated, using parallel computing to both identify material status and predict growth. The module monitors the material during growth, uses the Growth Models to assess conditions and predict outcomes, and maps these via a systematic logic to a parameter optimization table. This logic determines whether to optimize existing parameters or set new ones, bridging model outputs with reactor control commands. Each iterative cycle ends in a hardware action that steers the parameter space, enabling dynamic feedback control. In this closed-loop manner, the Growth Models efficiently analyze in situ data while real-time information iteratively guides parameter optimization until the parameter space converges to the target optimal window, ensuring the material remains within desired conditions until growth is complete. Following parameter initialization, the 100-nm GaAs is re-grown at an inferred temperature of 600°C based on the parameter initialization results. The substrate is subsequently cooled to an inferred temperature of 490°C for sequential growth of buried InAs QDs, followed by a 10-nm GaAs layer. The temperature is then raised to an inferred temperature of 600°C to grow an additional 100-nm GaAs. Finally, the substrate is cooled to the temperature used for buried InAs QDs growth, and the surface InAs QDs are grown starting from this temperature.

Combining PIML with on-the-fly feedback control, SemiEpi automates execution and analysis. This approach allows for dynamic optimization of these parameters within a single experiment, minimizing the need for multiple experimental runs and resulting in more efficient and effective material growth. SemiEpi is a self-driving setup that offers substantial advantages over conventional methods by customizing growth conditions for each substrate, ensuring compatibility across different reactors without requiring code modifications. It eliminates the need for extensive semiconductor or MBE process expertise to achieve the best results, making it a valuable platform for use in any epitaxy laboratory regardless of the grower’s level of knowledge and experience.

### SemiEpi configuration

#### Software and Hardware

Existing industrial software functions as isolated, sequential processing systems that maintain a fundamental disconnect between material growth and characterization data. This segregation hinders effective data fusion between material growth and characterization systems, making it difficult to adjust growth parameters in real-time. Additionally, most industrial software prioritizes automating predefined steps rather than intelligently evaluating or adjusting the growth condition based on real-time data.

SemiEpi was developed using LabVIEW. It utilizes Virtual Instrument Software Architecture (NI-VISA), a standard industry protocol for instrument communication, to control parameters for MBE. Specifically, it communicates with address-specific controllers through the Modbus protocol (a standard industrial serial communication protocol), allowing for the reading and writing of commands to control temperatures. Additionally, it employs direct binary serial commands, where specific bit positions correspond to the states of individual shutters, enabling precise control over different shutters. It also utilizes NI VISION to acquire RHEED video from the fluorescent screen. The acquired data is processed using Python libraries and transferred to the ML model, formatted in the Open Neural Network Exchange (ONNX) format, an open standard for ensuring model compatibility and interoperability across different frameworks, and optimized with TensorRT, a high-performance deep learning inference software development kit (SDK) and runtime library, for faster inference (see notes S1 and S2).

SemiEpi combines a standard MBE reactor, a camera, temperature controllers, shutter controllers, and an in situ RHEED system to enable monitoring and optimization. It was designed and deployed on a Windows 10 system with an AMD R9 7950X CPU, 64GB of RAM, an NVIDIA 3090 graphics card, and a 2 TB solid-state drive. The system is linked to a temperature controller and a shutter controller via USB 2.0 for data exchange. The Modbus protocol enables the connection of multiple temperature controllers in series, along with precise control of the In and Ga effusion cells using addresses. USB 3.0 allows the connection of a camera in a dark room outside the fluorescent screen. Furthermore, the model training and data preprocessing processes used by SemiEpi are also conducted on the system (see note S3).

#### PIML model construction and utilization

**Fig. 2.**
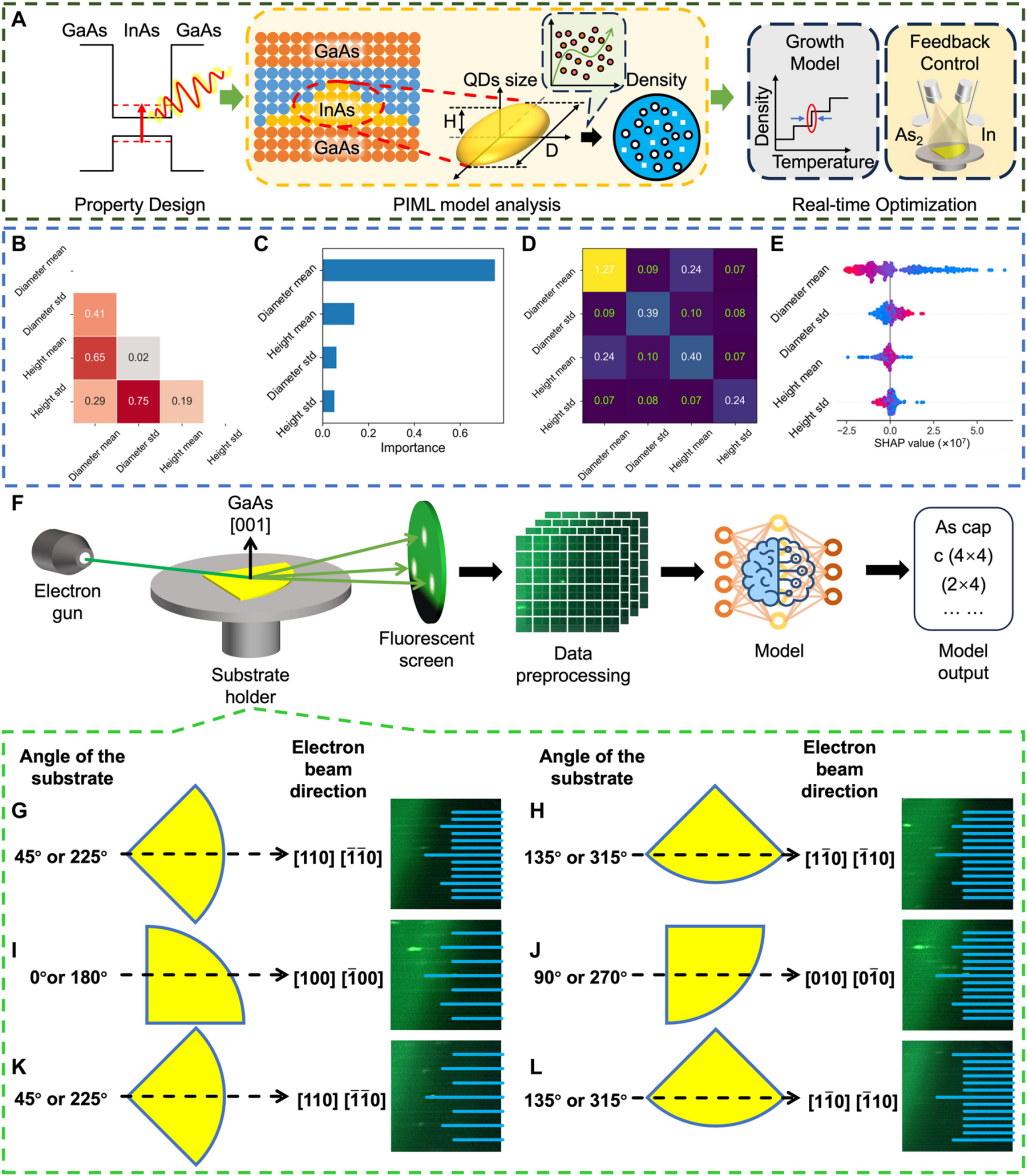
The QDs size feature analysis results and RHEED data process methods. (**A**) The process to design and grow QDs with emission wavelengths. (**B**) The workflow for RHEED data acquisition and processing. The feature analysis of CatBoost model. (**C**) The feature correlation, (**D**) feature importance, (**E**) feature interaction heat map, and (**F**) SHAP summary plot of QD size data. (**G** to **L**) Typical RHEED images obtained at various substrate angles. The blue lines on the images represent reconstruction streaks. The blue lines on the images represent diffraction features: the longest lines mark the specular spots, medium-length lines indicate integer order streaks, and the shortest lines denote half-integer order streaks.

SemiEpi begins with Property Design, where a QD heterostructure is designed for an emission wavelength of 1240 nm, suitable for gas sensing applications (see Fig. 2A) (*38*). The PIML model is implemented in two stages to achieve this design. First, the target wavelength is input into a physics model that simulates QDs with finite potential barriers (see note S4) (*39*). By performing material modeling and simulation within the PIML model analysis framework, the system establishes a quantitative relationship between desired performance and core QD size. Unlike simplified geometric models, this framework accounts for the specific geometric shape of the QDs and the heterogeneous material parameters, such as potential energy barriers and effective mass mismatches between the QD and the barrier materials (*40*, *41*). Incorporating these factors related to both shape and material allows the model to establish a precise quantitative link between target wavelengths and structural specifications. Due to fluctuations during growth and material inhomogeneity, QDs emitting the same target wavelength can vary in size. As a result, the model is executed multiple times, and the mean and standard deviation (std) of the results are statistically analyzed. Ultimately, the model predicts that the QDs have a mean diameter of 20.90 nm with a std. of 2.74 nm and a mean height of approximately 5.56 nm with a std. of 0.98 nm. Consequently, the predicted QD sizes can be used to correspondingly infer their density.

Second, we estimate QD density ranges from predicted sizes to enable indirect monitoring. Determining the average QD size directly is only feasible after the growth transition from a 2D wetting layer to a 3D island-like structure, and the size evolution typically happens within just a few seconds. This narrow time window limits the opportunity to optimize parameters to achieve the desired QD properties (*42*). However, previous studies have shown that RHEED can predict changes in QD density before QD formation (*16*). As an in situ tool, RHEED captures real-time surface reconstruction dynamics, where the chevron intensity and streak features provide a more direct physical manifestation of nucleation density than the final PL intensity (*27*–*29*, *43*). Consequently, in the material growth stage, the RHEED feedback loop correlates RHEED patterns directly with QD density to ensure high-fidelity control, rather than using size parameters as direct real-time inputs. Since QD size is correlated with density, this presents an opportunity to indirectly infer QD size by observing QD density (*16*). To this end, the PIML model estimates QD density ranges and values based on QD size. Note that a direct mapping from RHEED to PL is fundamentally limited. RHEED probes surface reconstruction and early-stage nucleation kinetics in situ, whereas PL is an ex-situ observable that depends not only on quantum dot formation but also on carrier recombination dynamics, defect states, and post-growth conditions. As a result, the correlation between RHEED and PL is indirect and results in noisy, inconsistent datasets with poor physical correspondence. Introducing QD density as an intermediate variable provides a physically meaningful bridge between growth dynamics and optical response. This decoupling separates the controllable in situ growth process from the structure–property relationship, enabling both interpretability and real-time control. We collected approximately 380 data points from atomic force microscopy (AFM) to create a robust dataset, which facilitates modeling the relationship between QD density and size. Finally, the workflow culminates in the Real-time Optimization phase. Here, SemiEpi leverages the integrated Growth Model and Feedback Control hardware to execute on-the-fly optimization, fabricating QDs that achieve the targeted emission properties with high precision.

We conducted a comprehensive analysis of the dataset to model the relationship between QD density and size (see note S5). The feature correlation heatmap revealed a strong positive correlation between “Diameter std” and “Height std”, with a coefficient of 0.75 (see Fig. 2B). Furthermore, the feature importance plot indicated that “Diameter mean” was the most significant predictor for density estimation (see Fig. 2C). The feature interaction heatmap showed that the combination of “Diameter mean” and “Height mean” exhibited the strongest interaction intensity, making it the most crucial pairing in our analysis (see Fig. 2D). The SHapley Additive exPlanations (SHAP) summary plot—a game-theoretic approach used to explain the output of ML models—clarified that higher values of “Diameter mean” consistently resulted in positive effects on density predictions (*44*). In contrast, “Height std” displayed bidirectional effects (see Fig. 2E). When constructing the PIML model to convert QD size to density, we selected the core input parameters: “Diameter mean”, “Diameter std”, “Height mean”, and “Height std”. We also screened and compared several regression models based on their performance, ultimately choosing the CatBoost model (see note S6). Using the QD size data obtained from the first stage of the PIML model, the inference from the CatBoost model suggests that the required QD density ranges from 4.4 × 10^10^ cm^−2^ to 5.5 × 10^10^ cm^−2^ as the std. increases (*45*).

#### RHEED data acquisition and feature analysis

During growth, the electron beam from the RHEED gun interacts with the continuously rotating sample, producing a diffracted pattern on the fluorescent screen (see Fig. 2F). A reminder is triggered to ensure adequate time to manually open the RHEED fluorescent screen shutter before capturing and preprocessing real-time RHEED images, which are then sent to models for analysis. The preprocessing methods used in this study align with previously reported methods (*18*). Each frame of the RHEED data is processed as a single channel of luminance information. These processed data are then stacked along an additional dimension to form a three-dimensional array, which serves as the input sample for the models. Thus, RHEED data are continuously recorded and analyzed in real-time, enabling precise determination of the critical transition temperature. While manual adjustments to the RHEED power supply and the fluorescent screen shutter were necessary in this study, these processes could be improved for computer control by integrating an RHEED system with the optional “microprocessor-controlled beam current regulation” feature and upgrading the fluorescent screen shutter to an automatic control system.

Gathering as many temperature points as possible is essential to obtain the parameter initialization curve. SemiEpi focuses on several vital temperatures, such as the deoxidation temperature, the transition temperature of As cap/c(4 × 4), c(4 × 4)/(2 × 4), and (2 × 4)/(n × 6). We reported RHEED characteristics of GaAs deoxidation, corresponding to the theoretical temperature of 580°C (*30*, *46*). After deoxidation, the substrate temperature is reduced and subsequently increased to observe the transition from the As cap to the (n × 6) stage (*34*, *47*). By observing the brightness and spacing of RHEED streaks from different angles, the ×4 reconstruction line can be identified from two angles, corresponding to c(4 × 4) (see Fig. 2, G and H). As the temperature increases, thermal vibrations of surface atoms intensify, leading to atomic reconstructions. Consequently, a gradual transition from c(4 × 4) to (2 × 4) can be observed around the theoretical temperature of 510°C (see Fig. 2, I and J) (*36*). When the temperature exceeds a theoretical temperature of 620°C, the (2 × 4) becomes increasingly blurred, and the (n × 6) gradually appears due to the lattice expansion of the GaAs crystal (*31*). In this study, ×2 and ×6 reconstruction lines can be observed from two angles, corresponding to the (2 × 6) structure (see Fig. 2, K and L). Although RHEED patterns exhibit inherent noise from the high camera gain required to detect faint diffraction features, this noise is distinct from the streaks’ spatial structure and does not compromise the identification of surface reconstruction states.

We used a dataset of 38 samples to develop the Initialization Model and Growth Models. Of these, 8 samples were GaAs substrates observed at different temperatures. Since the GaAs surface undergoes a gradual transformation, we collected data from each sample multiple times, with each video lasting at least 20 minutes. The remaining 30 samples were InAs QDs grown under various conditions (see note S7). The growth for each of these samples was repeated multiple times, with each video recording lasting at least 90 seconds. The dataset used for model training consists of various RHEED images, including clear and blurred examples.

During data preprocessing, image augmentation was applied to simulate experimental fluctuations without discarding critical physical information. Adjustments to brightness, saturation, and contrast mimicked variations in the RHEED power supply and imaging conditions, while noise addition and blurriness simulated mechanical vibrations and beam instability (*48*). Additionally, random cropping was performed by extracting a 400 × 400-pixel square from a starting coordinate randomly shifted within a 50 × 50-pixel range near the top-left corner of the much larger original image. This slight offset effectively increased data diversity and simulated physical electron beam drift or enhanced tolerance to Region of Interest (ROI) alignment errors. Crucially, these operations preserved the essential physical information—the geometric arrangement and relative intensity contrast of diffraction streaks—while forcing the model to learn invariant features, thereby enhancing robustness to degraded inputs without losing critical surface evolution parameters. We then converted images into single-channel intensity images for model input (*49*). This approach enabled the model to effectively process more degraded images during validation, enhancing its generalizability. The final dataset comprised approximately 370,000 NumPy arrays, generated from about 100 raw RHEED videos (8 fps, 90–130 s each). To capture temporal dynamics, a sliding window with a 1-frame stride was used to concatenate 24 consecutive frames into each sample. Each sample then underwent five independent rounds of random augmentation and normalization before conversion to NPY format.

#### Initialization and Growth Models’ construction and evaluation

**Fig. 3.**
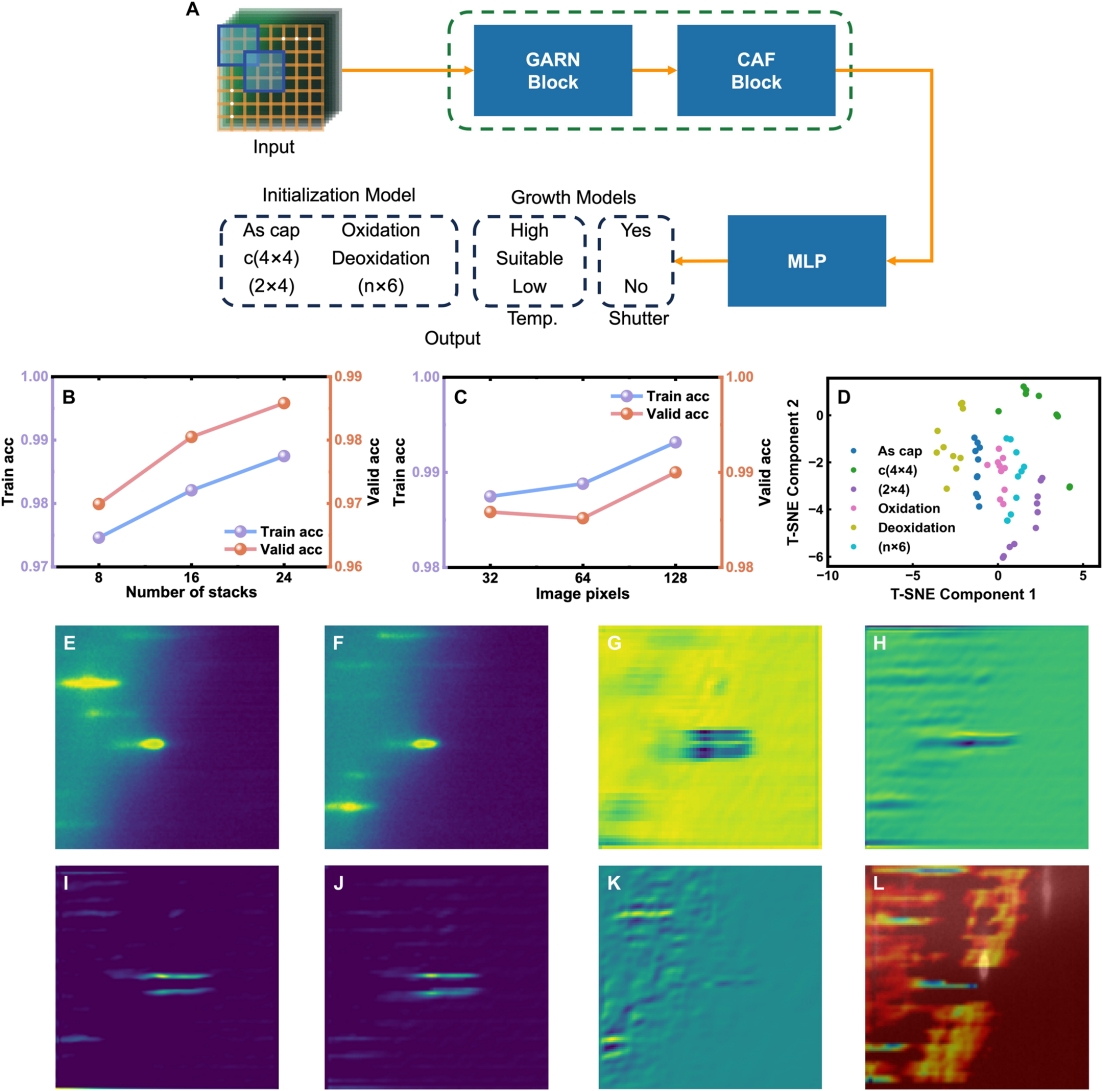
Initialization and Growth Models’ construction and evaluation. (**A**) A simplified architectural diagram of the construction of the “Initialization Model” and “Growth Models.” The variation of model validation accuracy and validation loss under different (**B**) heads and depths and (**C**) image stacks. Model feature processing: (**D**) t-SNE visualization of high-dimensional features. (**E** and **F**) Typical RHEED images. (**G** and **H**) Convolutional layer feature maps, (**I** and **J**) Attention heat maps, (**K**) Combined feature map, (**L**) Gradient-weighted class activation mapping results.

To enable self-driving heterostructure growth, SemiEpi integrates specialized ML models optimized to analyze RHEED data across its different operational processes: substrate deoxidation, parameter initialization, and material growth. The conventional convolutional neural network (CNN) approach processes all color channels in an image simultaneously, integrating information from each channel to extract features. This is effective for images with rich color information (*50*). However, it is less practical for datasets comprising multiple stacks of single-channel luminance information, such as RHEED data acquired from different angles of a continuously rotating substrate. To address this issue, a model must emphasize channel information, strengthen inter-channel correlations, and enhance data processing efficiency (*51*). Therefore, we introduce the Global Attention Residual Network (GARN) block and the Cross-layer Adaptive Fusion (CAF) block, both specifically developed to prioritize angle-dependent channel information (see note S8). The GARN block leverages a channel attention mechanism to automatically weight the most informative rotation angles. In parallel, the CAF block leverages a transformer architecture to capture global dependencies across image blocks; this design provides robustness against substrate “wobbling”, a condition where essential diffraction information shifts spatially. This study constructs three different models to address the distinct physical transitions of the growth process: an “Initialization Model” and two “Growth Models” (the “Temperature Model” and the “Shutter Model”) (see Fig. 3A). These models are designed for a multi-class classification task, with “Initialization Model” identifying reconstruction and deoxidation states, “Temperature Model” predicting the optimal InAs growth temperature, and “Shutter Model” evaluating the completion conditions for InAs QD growth.

The number of stacks in the Initialization Model and Growth Models is crucial for delivering richer input, especially when analyzing RHEED data, which experiences periodic changes. The distinctness of RHEED diffraction features naturally fluctuates across different rotation angles. If the number of image stacks is too small, the training samples may exhibit insufficient feature density in certain orientations. This can lead to inconsistent training outcomes, unstable validation performance, and even overfitting. Therefore, we optimized the model input sizes and determined that the best training performance was achieved using 24 images per sample, each with a resolution of 128 × 128 pixels (see Fig. 3, B and C). The accuracy is calculated as the ratio of correctly predicted labels to the total number of predictions. In our work, we selected the cross-entropy loss function, a well-established choice for classification tasks. This function guides the model in adjusting its weights and biases by minimizing the loss, thereby enhancing the precision of predictions (*52*). To expedite the training process, we employed the stochastic gradient descent optimizer, which effectively updates the model parameters during each iteration. The resulting validation accuracies for the “Initialization Model”, “Temperature Model”, and “Shutter Model” reached 99.1%, 99.6%, and 99.9%, respectively. To assess the necessity of the GARN-CAF architecture, we conducted a comparative study with alternative models and found that GARN-CAF substantially outperforms them (see note S9).

We analyzed alignment features of the “Initialization Model”. Using t-Distributed Stochastic Neighbor Embedding (t-SNE) analysis, we observed a clear separation between different color points, indicating the model effectively identifies both deoxidation and various reconstruction states (see Fig. 3D) (*53*). We also extracted two frames of typical RHEED maps from a sample, both showing prominent ×2 (see Fig. 3, E and F). By plotting the convolutional features maps, we observed that the convolution primarily focuses on streak-like features in RHEED images, which correspond to different reconstruction streaks, demonstrating high interpretability (see Fig. 3, G and H). When analyzing the attention heat map of the model with these two typical RHEED frames as inputs, it is evident that non-streak features appear dark, indicating that the model did not focus on these regions (see Fig. 3, I and J). Conversely, the brighter regions highlight streak-shaped features. Feature maps were also combined from multiple images to identify focal regions across the model. These plots highlight streak features on the left side (see Fig. 3K). In contrast, the right side, consisting of non-streak features, is mainly flat. This indicates the model effectively focuses on regions with distinct features. Additionally, we used Gradient-weighted Class Activation Mapping (Grad-CAM)—a technique for producing visual explanations by identifying regions high importance to the classification—to analyze the contribution of each region to the classification results (see Fig. 3L) (*54*). The Grad-CAM results highlight multiple streak features, which align well with the streak feature locations in the input image, demonstrating that the model has high sensitivity in data processing.

#### Data labeling

The dataset used to train the “Initialization Model” was collected through real-time RHEED imaging of a GaAs substrate during its deoxidation process, as well as from various reconstruction states observed at different temperatures during the heating of the smooth GaAs surface, including the “Oxidation”, “Deoxidation”, As cap, c(4 × 4), (2 × 4), and (n × 6) phases. These stages were recorded to create the dataset for training the model and labeled accordingly. Data corresponding to intermediate surface reconstruction stages were excluded from the dataset due to the challenges in assigning clear labels to these transitional states. As a result, the model recognizes a successful transformation of the material’s surface when it consistently identifies a newly reconstructed state multiple times in succession.

We utilized data from InAs QDs growth under various conditions to train the “Temperature Model” and “Shutter Model”. By characterizing the QD density using AFM, we analyzed how different growth temperatures affected its density, enabling us to design temperature regulation strategies for achieving the desired density of QDs. The analysis revealed that higher substrate temperatures during growth facilitate the formation of low-density QDs, whereas lower temperatures favor high-density QDs. To enhance the generalization capability of our classification model, particularly for samples near the decision boundary where label overlap may occur, we adopt a broader QD density range. This expanded range encompasses the density values predicted by the PIML model and targeted during material growth. Moreover, using a wider range of density labels better reflects the natural fluctuations observed in practice and helps reduce validation errors in QD density measurements obtained from AFM, thereby improving the overall robustness of the model. We observed that the density of InAs QDs for a deposition amount of 2.6 ML ranges between 4 × 10^10^ cm^−2^ and 6 × 10^10^ cm^−2^ (*55*). Therefore, within the “Temperature Model”, RHEED data from samples with densities within this density range were categorized as “Suitable”. Data from samples with densities below 4 × 10^10^ cm^−2^ were categorized as “High”. If the model output shows “High”, the substrate temperature is too high and should be decreased. Conversely, data from samples with densities above 6 × 10^10^ cm^−2^ were labeled “Low”. If the model output indicates “Low”, this indicates the substrate temperature is too low and should be increased. This mapping of model-generated labels to temperatures enables SemiEpi to provide optimized guidance throughout the growth process.

To achieve the desired QD density accurately, precise control of the growth temperature and timely completion of the growth process is essential. RHEED images captured facilitate the identification of the growth stages both before and after the InAs QD formation, which provided the necessary labels for the “Shutter Model”. The RHEED patterns before and 10 seconds after the end of growth of samples with QD densities in the range of 4–6 × 10^10^ cm^−2^ were categorized as “Yes”, while those outside this range were categorized as “No”. This approach ensures the timely closure of the In shutter when the QD density reaches 4–6 × 10^10^ cm^−2^.

The “Initialization Model” and the “Shutter Model” are discriminative models that evaluate the material’s state to identify and optimize the InAs QD growth conditions in real time. By determining the optimal closure timing, the “Shutter Model” essentially achieves precise control over the growth duration and In deposition amount. In contrast, the “Temperature Model” is a predictive model that forecasts the performance of samples upon growth completion. This model facilitates real-time predictions of QD density during growth, enabling a bivariate control system with timely parameter adjustments and ensuring high-quality material growth.

### Experiment validation

#### Parameter initialization

In our study, thermocouples are used to measure temperatures; however, temperature readings from multiple reconstruction transition points exhibit a non-linear relationship with the corresponding theoretical temperatures. A curve fitting approach is necessary to achieve a more reliable correlation (*56*). We compared different curve-fitting methods and found that the quadratic curve-fitting results were better than those obtained with linear or higher-order polynomial fits (see note S10) (*57*).

The parameter initialization curve extracts the thermocouple temperatures corresponding to the growth conditions to achieve the desired QD density, which can be achieved at a theoretical growth temperature of 490°C with a low growth rate. While it is possible to optimize the growth temperature for GaAs during the process, x-ray diffraction (XRD) and AFM results indicate variations around the theoretical temperature of 600°C have minimal impact on the quality of the material (see note S11). Therefore, with the parameter initialization curve, the thermocouple temperatures corresponding to the theoretical temperature of 490°C and 600°C were established for the growth of InAs QDs and GaAs.

**Fig. 4.**
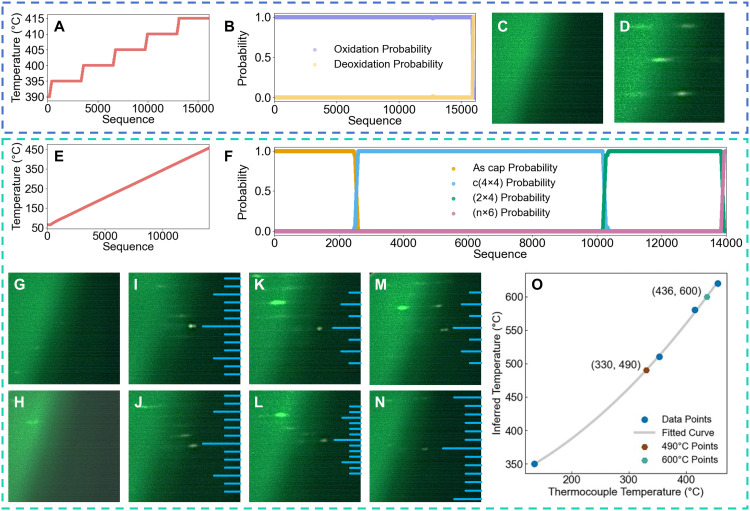
The parameter initialization. (**A**) Substrate temperature and (**B**) the running average results of “Initialization Model” output during substrate deoxidation. The RHEED image captured at around (**C**) 13,000th and (**D**) 16,000th sequence of (a). (**E**) Substrate temperature and (**F**) the running average results of “Initialization Model” output during parameter initialization. The RHEED image captured at around (**G** and **H**) 2000th, (**I** and **J**) 3000th, (**K** and **L**) 11,000th, and (**M** and **N**) 14,000th sequence of (E). RHEED images were captured from two angles. The blue lines on the images represent diffraction features: the longest lines mark the specular spots, medium-length lines indicate integer order streaks, and the shortest lines denote half-integer order streaks. (**O**) Parameter initialization results. Source data are provided as data S1.

The main objective of SemiEpi is to generate parameter initialization curves and optimize growth parameters for InAs QDs with desired wavelength. Real-time data recorded by SemiEpi around different transition points was analyzed (see Fig. 4). During the automatic deoxidation process, SemiEpi heated up in increments of 5°C, from the thermocouple temperature of 390°C to 415°C through five increments (see Fig. 4A). The RHEED screen showed no distinctive features until the thermocouple temperature of 415°C, when distinct bright spot features appeared (see Fig. 4, C and D). Analysis of the “Initialization Model” output during deoxidation revealed that, from the 0th to around the 16,000th sequence, the oxidation probability remained close to 1, indicating that the model did not identify the deoxidation state. After the 16,000th sequence, the deoxidation probability rapidly increased to nearly 1, confirming the model’s accurate identification of the deoxidation state (see Fig. 4B).

After growing a layer of GaAs on the deoxidized sample and cooling, the substrate was gradually heated at a rate of 15°C per minute (see Fig. 4E). Real-time RHEED data collected during this process was analyzed using the “Initialization Model” to monitor reconstruction states (see Fig. 4F). Initially, the model identified only “As cap” labels, with RHEED patterns showing diffuse features from the beginning to around the 2,000th sequence (see Fig. 4, G and H). By around the 3,000th sequence, at a thermocouple temperature of 135°C, a marked shift occurred as the probability of “As cap” labels dropped sharply and “c(4 × 4)” labels became dominant. The RHEED patterns began exhibiting periodic features consistent with the emergence of a × 4 reconstruction (see Fig. 4, I and J). At around the 11,000th sequence, as the substrate heating reached 353°C, the probability of “(2 × 4)” labels increased significantly. This shift indicated a structural change on the surface, supported by RHEED observations of features combining ×2 and ×4 (see Fig. 4, K and L). Finally, by the 14,000th sequence, the model output showed an increased probability for the “(n × 6)” label. At this stage, the thermocouple temperature reached 455°C, and the RHEED patterns displayed a distinct ×6 periodicity alongside residual ×2 features (see Fig. 4, M and N). The results demonstrate the model’s high sensitivity in distinguishing between different structural states.

Finally, SemiEpi used a quadratic curve to fit the collected thermocouple temperature data to inferred temperature data. This sets the initial thermocouple temperatures for InAs growth at 330°C and for GaAs growth at 436°C (see Fig. 4O).

#### QD growth

**Fig. 5.**
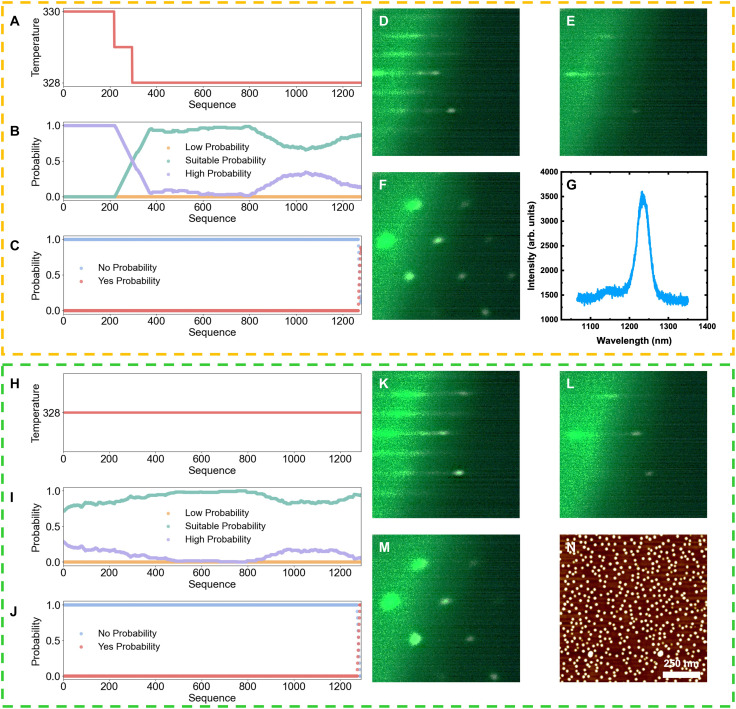
The growth of the InAs QDs. (**A**) Substrate temperature, and the running average results of (**B**) “Temperature Model” and (**C**) “Shutter Model” output during growth buried InAs QDs. The RHEED image captured at around (**D**) 100th, (**E**) 700th, and (**F**) 1300th sequence of (A). (**G**) PL spectrum of the buried QDs. (**H**) Substrate temperature, and the running average results of (**I**) “Temperature Model” and (**J**) “Shutter Model” output during growth surface InAs QDs. The RHEED image captured at around (**K**) 100th, (**L**) 700th, and (**M**) 1300th sequence of (H). (**N**) The 1 μm × 1 μm AFM image of the surface QDs. Source data are provided as data S1.

The density of InAs QDs is highly sensitive to temperatures. Based on the output of the PIML model, SemiEpi uses RHEED to analyze the material surface and optimize the temperature in real-time to ensure that the samples achieve the desired density range of 4–6 × 10^10^ cm^−2^ (see Fig. 5) (see movie S1 for the experiment of QD growth). During the growth of buried InAs QDs, the thermocouple temperature analysis revealed a 2°C decrease, consistent with the trend of QD density change relative to thermocouple temperature (see Fig. 5A and note S12). This minor adjustment specifically highlights the high precision of the parameter initialization module, as a perfectly initialized system requires only minimal fine-tuning to remain within the optimal growth window, rather than non-monotonic fluctuations that would suggest system instability. Convergence to the target state is achieved through continuous, real-time feedback during growth. Using RHEED input and growth models, the system dynamically compensates for fluctuations and steers itself toward the desired density window. This dynamic adjustment is essential, as even small deviations during growth can lead to notable variations in the final QD properties. Statistical analysis of RHEED data using the “Temperature Model” reveals that, in the early stages, the model predominantly outputs the “High” label (see Fig. 5B). As growth progresses, the probability of the model outputting the “Suitable” label increases around the 200th sequence. Subsequently, the model mainly outputs “Suitable” labels until the end of the growth. The predominance of the “Suitable” label indicates that the growth process is operating within the optimal window. The probability of the “Low” label remains consistently low, indicating the initial growth temperature was too high and required adjustment to achieve the desired density.

The “Shutter Model” analysis indicates that from the 0th to approximately the 1200th sequence, the model primarily outputs “No”, suggesting the InAs QD growth has not yet achieved the desired density (see Fig. 5C). However, starting from the 1272th sequence, the probability of outputting “Yes” increases significantly, and the system executes the shutter closure at the 1280th sequence, indicating the desired QD density is achieved. Once the Shutter Model identifies a “Yes” trigger, growth and inference terminate immediately. Analysis of RHEED data reveals that from the 100th and 700th sequences, RHEED patterns exhibit evident streak characteristics with no QDs formed (see Fig. 5, D and E). In contrast, RHEED patterns from the 1300th sequence reveal well-formed, rounded, and neatly aligned spots, indicating successful QD formation (see Fig. 5F).

Additionally, the sample prepared using SemiEpi exhibited a photoluminescence (PL) intensity of 3605 and a full width at half maximum (FWHM) of 29.13 meV at the wavelength of 1235 nm. The FWHM of our SemiEpi-grown sample is narrower than typically reported values for single-layer InAs QDs, which often exceed 35–40 meV (*58*, *59*). This performance significantly surpassed that of the reference sample prepared using conventional methods, which exhibited a PL intensity of 2270 and an FWHM of 34.70 meV at the wavelength of 1229 nm. Additionally, it outperformed the reference sample prepared using parameter initialization, which exhibited a PL intensity of 2531 and an FWHM of 30.79 meV at the wavelength of 1225 nm (see Fig. 5G and notes S13 and S14). The obtained effective lifetimes of 2.06 ns from time-resolved photoluminescence (TRPL) measurements demonstrate the high quality of the QDs prepared by SemiEpi, providing strong evidence for the reliability of the platform (see note S15). It is worth noting that the emission wavelengths of samples prepared with the SemiEpi closely match the desired wavelength of 1240 nm. In contrast, the two reference samples exhibit greater deviations from the desired emission wavelengths.

SemiEpi also grew surface InAs QDs to observe their morphology. The initial growth temperature for these QDs was set to the thermocouple temperature of 328°C, which was the temperature for buried InAs QDs (see Fig. 5H). During growth, the thermocouple temperature remained stable at 328°C. This highlights that adjusting the temperature during the growth of buried InAs QDs allowed achieving the desired QD density without the need for additional temperature adjustments. The probabilistic statistical analysis of the “Temperature Model” output during the process shows that initially, the model predominantly outputs “Suitable” labels, with only a few “High” labels (see Fig. 5I). This indicates that the initial growth temperature is appropriate. The analysis of the “Shutter Model” output reveals a significant increase in the probability of the “Yes” label starting from the 1280th sequence, with the shutter closure executed at the 1291th sequence, indicating that the QDs have achieved the desired QD density (see Fig. 5J).

In the RHEED patterns obtained at the 100th, 700th, and 1300th sequences, we observe a transition from streak to spot features as the growth progresses (see Fig. 5, K to M). The spot pattern at the 1300th sequence appears well-ordered and rounded. AFM characterization of the surface InAs QDs indicates a high QD density of approximately 5 × 10^10^ cm^−2^, with a relatively uniform distribution (see Fig. 5N). In addition, the reference sample prepared using parameter initialization achieved a density of 5.4 × 10^10^ cm^−2^. However, the reference sample prepared using conventional methods only achieved a QD density of 3.7 × 10^10^ cm^−2^, falling below the range designated as “Suitable”. This highlights the effectiveness of parameter initialization in improving growth outcomes. The high reproducibility of the system was further demonstrated through five supplementary growth experiments conducted with SemiEpi, with all resulting samples consistent with the intended design parameters (see note S16). The desired outcomes were successfully achieved in an additional experiment conducted on a different MBE reactor, validating the hardware independence of the approach (see note S17).

## DISCUSSION

In this work, we present a self-driving approach for on-demand heterostructure growth. We introduce SemiEpi, a system that designs heterostructures using PIML models while leveraging the substrate’s intrinsic properties to establish optimal initial conditions. These conditions are proposed for experimental validation by aligning the thermocouple temperature with theoretical values through parameter initialization. It continuously monitors and analyzes these conditions using ML algorithms to predict outcomes, dynamically adjusting them throughout the InAs QDs growth process. This method effectively maps the relationships between material status and their outcomes, enhancing material quality and ensuring consistent and reliable results.

The QDs’ performance using SemiEpi aligns with the expected characteristics of QD lasers. However, our experiments are facing some challenges, including a failure rate associated with temperature change rates and the similarity of data characteristics. The similarity in RHEED patterns between the As cap layer and the oxidation state complicates the identification process and impacts the dataset construction. This emphasizes the need for more accurate labeling and clearer differentiation between these states.

In summary, SemiEpi integrates PIML models, prior semiconductor knowledge, and real-time feedback control during experiments, offering a robust framework to address hardware disparities between systems-a key source of unpredictable results in semiconductor research. The field faces inherent data limitations due to the high cost and prolonged turnaround. SemiEpi overcomes the limitations of traditional methods, which rely on extensive datasets and fixed programs while being highly sensitive to reactor geometry, operational status, and sample inconsistencies. The advantage of SemiEpi is not solely reflected in the magnitude of performance improvement, but in the paradigm of optimization. Conventional approaches rely on open-loop, recipe-based tuning guided by empirical experience, whereas SemiEpi performs closed-loop optimization within a single growth process. By identifying growth stages and minimizing cross-reactor variability through parameter initialization, the platform ensures reproducible growth conditions. We utilized RHEED for analysis, but the system is readily adaptable to other in situ techniques, such as absorption spectroscopy and mass spectrometry. By dynamic parameter adjustments, SemiEpi effectively explores and optimizes the parameter space linking in situ data and material growth results, ensuring that growth conditions remain stable and reproducible, regardless of external fluctuations.

We utilize existing physical laws to design sizes that achieve the desired emission wavelengths. Additionally, we apply ML to uncover high-dimensional, nonlinear relationships to establish correlations between size and density when the physical laws for these variables are not available. Beyond its applicability to InAs/GaAs and other III-V systems (e.g., InAs/InP), the framework is readily extended to integrate various in situ diagnostics beyond RHEED—such as ellipsometry or absorption spectroscopy—broadening its utility across material platforms and monitoring techniques. Moreover, our method is scalable for large-scale material production, significantly reducing optimization cycles and improving yield quality. Moving forward, integrating portability, multimodality, and reinforcement learning into smart MBE systems could transform the future of precision material growth.

## MATERIALS AND METHODS

### Material growth

The InAs QD samples were grown on GaAs substrates using a Riber 32P MBE reactor. The system is equipped with an arsenic (As) valved cracker, as well as indium (In) and gallium (Ga) effusion cells. During the growth process, the As_2_ source was used, and the cracker temperature was maintained at over 900°C. Beam Equivalent Pressure (BEP) measurements were used to assess the fluxes and calibrate the ratios of group III and V elements. Substrate temperatures were monitored using Type-C thermocouples, and the growth rates were calibrated by observing RHEED oscillations from additional layers grown on the GaAs substrate. The BEP values for the cells were as follows: 6.8 × 10^−9^ Torr for In, 1.5 × 10^−7^ Torr and 9 × 10^−8^ Torr for Ga, and 2.5 × 10^−6^ Torr, 1.5 × 10^−6^ Torr, and 1.0 × 10^−6^ Torr for As used in GaAs, and InAs, respectively. Before the growth process, the n-GaAs substrates were outgassed in a buffer chamber at 350°C. The growth was managed by SemiEpi, which included steps for substrate deoxidation, parameter initialization, and material growth. The growth rates were 0.6 μm/h and 0.36 μm/h for GaAs, while the InAs growth rate was approximately 0.016 ML/s.

### Material characterization

RHEED was set up in the MBE growth chamber with a power supply at 12 kV and 1.49 A to generate an electron beam (RHEED 12 from STAIB Instruments). The electron beam interacts with the surface of the epitaxial layer, producing diffraction patterns that are then projected onto a fluorescent screen. These patterns are captured in real-time via a camera mounted outside the chamber in a dark room. Throughout the growth process, the substrate was rotated at one revolution every 3 seconds. The camera had an exposure time of 100 ms and a sampling rate of 8 frames per second, meaning that every 24 frames represented one full revolution of the substrate. After the growth was completed, the samples were characterized using a custom-built PL system (iHR 550 spectrometers from HORIBA). This system consists of an optical beam splitter, reflector, attenuator, and a 532 nm continuous wave excitation laser. An InGaAs detector within the spectrometer collected the light emitted by the samples. The surface morphology of the InAs QDs was also characterized using AFM (Dimension Icon from Burker). Time-resolved PL (TRPL) measurements were conducted at 130 K using a short-pulse laser (Chameleon Ultra from Coherent). The excitation power density was set at 0.76 W/cm^2^. The emitted PL was analyzed with a spectrometer (FHR 1000 from Horiba) and detected using an infrared single-photon detector (ID230 Infrared Single-Photon Detector from Picoquant).

## Supplementary Materials

### The PDF file includes:

Notes S1 to S17 Figs. S1 to S28 Tables S1 and S2 Legends for movies S1 to S3 Legend for data S1 References

## References

[1] Y. Suematsu, Dynamic Single-Mode Lasers. J. Light. Technol. 32, 1144–1158 (2014).

[2] M. Lee, J.-W. Jo, Y.-J. Kim, S. Choi, S. M. Kwon, S. P. Jeon, A. Facchetti, Y.-H. Kim, S. K. Park, Corrugated heterojunction metal-oxide thin-film transistors with high electron mobility via vertical interface manipulation. Adv Mater. 30, 1804120 (2018). 10.1002/adma.201804120 30152085

[3] J. Kwoen, Y. Arakawa, Multiclass classification of reflection high-energy electron diffraction patterns using deep learning. J. Cryst. Growth 593, 126780 (2022).

[4] T. Wu, S. Kheiri, R. J. Hickman, H. Tao, T. C. Wu, Z.-B. Yang, X. Ge, W. Zhang, M. Abolhasani, K. Liu, A. Aspuru-Guzik, E. Kumacheva, Self-driving lab for the photochemical synthesis of plasmonic nanoparticles with targeted structural and optical properties. Nat. Commun. 16, 1473 (2025). 39922810 10.1038/s41467-025-56788-9 PMC11807174

[5] A. Slattery, Z. Wen, P. Tenblad, J. Sanjosé-Orduna, D. Pintossi, T. den Hartog, T. Noël, Automated self-optimization, intensification, and scale-up of photocatalysis in flow. Science 383, eadj1817 (2024). 38271529 10.1126/science.adj1817

[6] K. Choudhary, B. DeCost, C. Chen, A. Jain, F. Tavazza, R. Cohn, C. W. Park, A. Choudhary, A. Agrawal, S. J. L. Billinge, E. Holm, S. P. Ong, C. Wolverton, Recent advances and applications of deep learning methods in materials science. npj Comput. Mater. 8, 59 (2022).

[7] G. E. Karniadakis, I. G. Kevrekidis, L. Lu, P. Perdikaris, S. Wang, L. Yang, Physics-informed machine learning. Nat. Rev. Phys. 3, 422–440 (2021).

[8] D. Bimberg, M. Grundmann, N. N. Ledentsov, *Quantum Dot Heterostructures*. (John Wiley & Sons, 1999).

[9] J. Liu, Y. Nie, W. Xue, L. Wu, H. Jin, G. Jin, Z. Zhai, C. Fu, Size effects on structural and optical properties of tin oxide quantum dots with enhanced quantum confinement. J. Mater. Res. Technol. 9, 8020–8028 (2020).

[10] P. B. Joyce, T. J. Krzyzewski, G. R. Bell, T. S. Jones, E. C. Le Ru, R. Murray, Optimizing the growth of 1.3 μm InAs/GaAs quantum dots. Phys. Rev. B 64, 235317 (2001).

[11] R. Heitz, I. Mukhametzhanov, A. Madhukar, A. Hoffmann, D. Bimberg, Temperature dependent optical properties of self-organized InAs/GaAs quantum dots. J. Electron. Mater. 28, 520–527 (1999).

[12] R. Schlereth, J. Hajer, L. Fürst, S. Schreyeck, H. Buhmann, L. W. Molenkamp, Band edge thermometry for the MBE growth of (Hg,Cd)Te-based materials. J. Cryst. Growth 537, 125602 (2020).

[13] M. F. Vilela, G. K. Pribil, K. R. Olsson, D. D. Lofgreen, HgCdTe molecular beam epitaxy growth temperature calibration using spectroscopic ellipsometry. J. Electron. Mater. 41, 2937–2942 (2012).

[14] S. E. Aleksandrov, G. A. Gavrilov, A. A. Kapralov, G. Y. Sotnikova, D. F. Chernykh, A. N. Alekseev, A. L. Dudin, I. V. Kogan, A. P. Shkurko, Pyrometer unit for GaAs substrate temperature control in an MBE system. Tech. Phys. 49, 123–127 (2004).

[15] G. R. Bell, J. G. Belk, C. F. McConville, T. S. Jones, Species intermixing and phase transitions on the reconstructed (001) surfaces of GaAs and InAs. Phys. Rev. B 59, 2947–2955 (1999).

[16] C. Shen, W. Zhan, K. Xin, M. Li, Z. Sun, H. Cong, C. Xu, J. Tang, Z. Wu, B. Xu, Z. Wei, C. Xue, C. Zhao, Z. Wang, Machine-learning-assisted and real-time-feedback-controlled growth of InAs/GaAs quantum dots. Nat. Commun. 15, 2724 (2024). 38553435 10.1038/s41467-024-47087-w PMC10980817

[17] C. Shen, W. Zhan, S. Pan, H. Hao, N. Zhuo, K. Xin, H. Cong, C. Xu, B. Xu, T. K. Ng, S. Chen, C. Xue, Z. Wang, C. Zhao, Real-time self-optimization of quantum dot laser emissions during machine learning-assisted epitaxy. Adv. Mater 12, 2503059 (2025). 10.1002/advs.202503059 PMC12279205 40317655

[18] C. Shen, W. Zhan, J. Tang, Z. Wu, B. Xu, C. Zhao, Z. Wang, Universal deoxidation of semiconductor substrates assisted by machine learning and real-time feedback control. ACS Appl. Mater. Interfaces 16, 18213–18221 (2024). 38554077 10.1021/acsami.4c01765

[19] S. Tao, M. Zhang, Z. Zhao, H. Li, R. Ma, Y. Che, X. Sun, L. Su, C. Sun, X. Chen, H. Chang, S. Zhou, Z. Li, H. Lin, Y. Liu, W. Yu, Z. Xu, H. Hao, S. Moura, X. Zhang, Y. Li, X. Hu, G. Zhou, Non-destructive degradation pattern decoupling for early battery trajectory prediction via physics-informed learning. Energy Environ. Sci. 18, 1544–1559 (2025).

[20] P. Nikolaev, D. Hooper, F. Webber, R. Rao, K. Decker, M. Krein, J. Poleski, R. Barto, B. Maruyama, Autonomy in materials research: A case study in carbon nanotube growth. npj Comput. Mater. 2, 16031 (2016).

[21] B. P. MacLeod, F. G. L. Parlane, T. D. Morrissey, F. Häse, L. M. Roch, K. E. Dettelbach, R. Moreira, L. P. E. Yunker, M. B. Rooney, J. R. Deeth, V. Lai, G. J. Ng, H. Situ, R. H. Zhang, M. S. Elliott, T. H. Haley, D. J. Dvorak, A. Aspuru-Guzik, J. E. Hein, C. P. Berlinguette, Self-driving laboratory for accelerated discovery of thin-film materials. Sci. Adv. 6, eaaz8867 (2020). 32426501 10.1126/sciadv.aaz8867 PMC7220369

[22] R. Shimizu, S. Kobayashi, Y. Watanabe, Y. Ando, T. Hitosugi, Autonomous materials synthesis by machine learning and robotics. APL Mater. 8, 111110 (2020).

[23] A. A. Volk, R. W. Epps, D. T. Yonemoto, B. S. Masters, F. N. Castellano, K. G. Reyes, M. Abolhasani, AlphaFlow: Autonomous discovery and optimization of multi-step chemistry using a self-driven fluidic lab guided by reinforcement learning. Nat. Commun. 14, 1403 (2023). 36918561 10.1038/s41467-023-37139-y PMC10015005

[24] S. B. Harris, A. Biswas, S. J. Yun, K. M. Roccapriore, C. M. Rouleau, A. A. Puretzky, R. K. Vasudevan, D. B. Geohegan, K. Xiao, Autonomous synthesis of thin film materials with pulsed laser deposition enabled by in situ spectroscopy and automation. Small Methods 8, 2301763 (2024). 10.1002/smtd.202301763 38678523

[25] D. M. Fébba, K. R. Talley, K. Johnson, S. Schaefer, S. R. Bauers, J. S. Mangum, R. W. Smaha, A. Zakutayev, Autonomous sputter synthesis of thin film nitrides with composition controlled by Bayesian optimization of optical plasma emission. APL Mater. 11, 071119 (2023).

[26] J. W. Lee, D. Schuh, M. Bichler, G. Abstreiter, Size and density estimation of self-assembled InAs quantum dots on GaAs(001) substrate through the analysis of RHEED patterns. Phys. Status Solidi C 0, 1121–1124 (2003).

[27] A. Freundlich, C. Rajapaksha, M. Gunasekera, in *37th IEEE Photovoltaic Specialists Conference* (2011), pp. 003483–003485.

[28] C. F. Schuck, R. A. McCown, A. Hush, A. Mello, S. Roy, J. W. Spinuzzi, B. Liang, D. L. Huffaker, P. J. Simmonds, Self-assembly of (111)-oriented tensile-strained quantum dots by molecular beam epitaxy. J. Vac. Sci. Technol. B 36, 031803 (2018).

[29] H. Z. Song, T. Usuki, Y. Nakata, N. Yokoyama, H. Sasakura, S. Muto, Formation of InAs/GaAs quantum dots from a subcritical InAs wetting layer: A reflection high-energy electron diffraction and theoretical study. Phys. Rev. B 73, 115327 (2006).

[30] M. Rei Vilar, J. El Beghdadi, F. Debontridder, R. Artzi, R. Naaman, A. M. Ferraria, A. M. Botelho do Rego, Characterization of wet-etched GaAs (100) surfaces. Surf. Interface Anal. 37, 673–682 (2005). 10.1021/la050682+ 16142959

[31] A. Ohtake, Surface reconstructions on GaAs(001). Surf. Sci. Rep. 63, 295–327 (2008).

[32] I. Karpov, N. Venkateswaran, G. Bratina, W. Gladfelter, A. Franciosi, L. Sorba, Arsenic cap layer desorption and the formation of GaAs(001)c(4×4) surfaces. J. Vac. Sci. Technol. B. Microelectron. 13, 2041–2048 (1995).

[33] T. T. Chiang, W. E. Spicer, Arsenic on GaAs: Fermi-level pinning and thermal desorption studies. J. Vac. Sci. Technol. C 7, 724–730 (1989).

[34] R. W. Bernstein, A. Borg, H. Husby, B. O. Fimland, J. K. Grepstad, Capping and decapping of MBE grown GaAs(001), Al_0.5_Ga_0.5_As(001), and AlAs(001) investigated with ASP, PES, LEED, and RHEED. Appl. Surf. Sci. 56, 74–80 (1992).

[35] U. Resch, N. Esser, Y. S. Raptis, W. Richter, J. Wasserfall, A. Förster, D. I. Westwood, Arsenic passivation of MBE grown GaAs(100): Structural and electronic properties of the decapped surfaces. Surf. Sci. 269, 797–803 (1992).

[36] A. Ohtake, M. Ozeki, T. Yasuda, T. Hanada, Atomic structure of the GaAs(001)-(2×4) surface under As flux. Phys. Rev. B 65, 165315 (2002).

[37] A. Ohtake, Structure and composition of Ga-rich (6×6) reconstructions on GaAs(001). Phys. Rev. B 75, 153302 (2007).

[38] T. Ritari, J. Tuominen, H. Ludvigsen, J. Petersen, T. Sørensen, T. P. Hansen, H. R. Simonsen, Gas sensing using air-guiding photonic bandgap fibers. Opt. Exp. 12, 4080–4087 (2004). 10.1364/opex.12.004080 19483949

[39] S. Kabi, A. G. U. Perera, Effect of quantum dot size and size distribution on the intersublevel transitions and absorption coefficients of III-V semiconductor quantum dot. J. Appl. Phys. 117, 124303 (2015).

[40] C. Y. Ngo, S. F. Yoon, W. J. Fan, S. J. Chua, Effects of size and shape on electronic states of quantum dots. Phys. Rev. B 74, 245331 (2006).

[41] L. Aderras, E. Feddi, A. Bah, F. Dujardin, C. A. Duque, On the electronic states in lens-shaped quantum dots. Phys. Status Solidi C 254, 1700144 (2017).

[42] Y. Berdnikov, P. Holewa, S. Kadkhodazadeh, J. M. Śmigiel, A. Sakanas, A. Frackowiak, K. Yvind, M. Syperek, E. Semenova, Near-critical Stranski-Krastanov growth of InAs/InP quantum dots. Sci. Rep. 14, 23697 (2024). 39390005 10.1038/s41598-024-70451-1 PMC11467334

[43] N. Bart, C. Dangel, P. Zajac, N. Spitzer, J. Ritzmann, M. Schmidt, H. G. Babin, R. Schott, S. R. Valentin, S. Scholz, Y. Wang, R. Uppu, D. Najer, M. C. Löbl, N. Tomm, A. Javadi, N. O. Antoniadis, L. Midolo, K. Müller, R. J. Warburton, P. Lodahl, A. D. Wieck, J. J. Finley, A. Ludwig, Wafer-scale epitaxial modulation of quantum dot density. Nat. Commun. 13, 1633 (2022). 35347120 10.1038/s41467-022-29116-8 PMC8960873

[44] Y. Nohara, K. Matsumoto, H. Soejima, N. Nakashima, Explanation of machine learning models using shapley additive explanation and application for real data in hospital. Comput. Methods Programs Biomed. 214, 106584 (2022). 34942412 10.1016/j.cmpb.2021.106584

[45] J. T. Hancock, T. M. Khoshgoftaar, CatBoost for big data: An interdisciplinary review. J. Big Data 7, 94 (2020). 33169094 10.1186/s40537-020-00369-8 PMC7610170

[46] A. Y. Cho, Growth of III–V semiconductors by molecular beam epitaxy and their properties. Thin Solid Films 100, 291–317 (1983).

[47] U. Resch, S. M. Scholz, U. Rossow, A. B. Müller, W. Richter, A. Förster, Thermal desorption of amorphous arsenic caps from GaAs(100) monitored by reflection anisotropy spectroscopy. Appl. Surf. Sci. 63, 106–110 (1993).

[48] Y. Qi, Z. Yang, W. Sun, M. Lou, J. Lian, W. Zhao, X. Deng, Y. Ma, A comprehensive overview of image enhancement techniques. Arch. Comput. Methods Eng. 29, 583–607 (2022).

[49] H. J. Kim, M. Chong, T. G. Rhee, Y. G. Khim, M.-H. Jung, Y.-M. Kim, H. Y. Jeong, B. K. Choi, Y. J. Chang, Machine-learning-assisted analysis of transition metal dichalcogenide thin-film growth. Nano Converg. 10, 10 (2023). 36806667 10.1186/s40580-023-00359-5 PMC9941396

[50] W. Xu, F. Gao, J. Zhang, X. Tao, A. Alkhateeb, Deep learning based channel covariance matrix estimation with user location and scene images. IEEE Trans. Commun. 69, 8145–8158 (2021).

[51] N. C. F. Codella, Q. B. Nguyen, S. Pankanti, D. A. Gutman, B. Helba, A. C. Halpern, J. R. Smith, Deep learning ensembles for melanoma recognition in dermoscopy images. IBM J. Res. Dev. 61, 5:1–5:15 (2017).

[52] U. Ruby, V. Yendapalli, Binary cross entropy with deep learning technique for image classification. Int. J. Adv. Trends Comput. Sci. Eng 9, 5393–5397 (2020).

[53] C. P. Roca, O. T. Burton, J. Neumann, S. Tareen, C. E. Whyte, V. Gergelits, R. V. Veiga, S. Humblet-Baron, A. Liston, A cross entropy test allows quantitative statistical comparison of t-SNE and UMAP representations. Cell Rep. Methods 3, 100390 (2023). 36814837 10.1016/j.crmeth.2022.100390 PMC9939422

[54] C. van Zyl, X. Ye, R. Naidoo, Harnessing eXplainable artificial intelligence for feature selection in time series energy forecasting: A comparative analysis of Grad-CAM and SHAP. Appl. Energy 353, 122079 (2024).

[55] S.-K. Park, J. Tatebayashi, Y. Arakawa, Formation of ultrahigh-density InAs/AlAs quantum dots by metalorganic chemical vapor deposition. Appl. Phys. Lett. 84, 1877–1879 (2004).

[56] Y. Ruan, J. Li, Q. Xiao, Y. Wu, M. Shi, High-temperature failure evolution analysis of k-type film thermocouples. Micromachines 14, 2070 (2023). 38004927 10.3390/mi14112070 PMC10672794

[57] V. A. Drebushchak, Thermocouples, their characteristic temperatures, and simple approximation of the emf vs. T. Thermochim. Acta 603, 218–226 (2015).

[58] L. Chu, M. Arzberger, G. Böhm, G. Abstreiter, Influence of growth conditions on the photoluminescence of self-assembled InAs/GaAs quantum dots. J. Appl. Phys. 85, 2355–2362 (1999).

[59] M.-Y. Kong, X.-L. Wang, D. Pan, Y.-P. Zeng, J. Wang, W. Ge, A comparison of photoluminescence properties of InGaAs/GaAs quantum dots with a single quantum well. J. Appl. Phys. 86, 1456–1459 (1999).

[60] D. Lu, J. Ahn, S. Freisem, D. Gazula, D. G. Deppe, Lens-shaped all-epitaxial quantum dot microcavity. Appl. Phys. Lett. 87, 163105 (2005).

[61] Y. Li, Z. Song, Z. Li, G. Sun, C. S. Tan, W. Fan, Q. J. Wang, Theoretical design of mid-infrared interband cascade lasers in SiGeSn system. N. J. Phys. 22, 083061 (2020).

[62] A. Maltsi, T. Niermann, T. Streckenbach, K. Tabelow, T. Koprucki, Numerical simulation of TEM images for In(Ga)As/GaAs quantum dots with various shapes. Opt. Quant. Electron. 52, 257 (2020).

[63] J. K. Kim, T. A. Strand, R. L. Naone, L. A. Coldren, Design parameters for lateral carrier confinement in quantum-dot lasers. Appl. Phys. Lett. 74, 2752–2754 (1999).

[64] J. Shumway, A. J. Williamson, A. Zunger, A. Passaseo, M. DeGiorgi, R. Cingolani, M. Catalano, P. Crozier, Electronic structure consequences of In/Ga composition variations in self-assembled In_x_Ga_1-x_As/GaAs alloy quantum dots. Phys. Rev. B 64, 125302 (2001).

[65] A. N. Kosarev, V. V. Chaldyshev, Carrier localization by a quantum dot in a quantum well. Phys. Rev. Appl. 16, 044046 (2021).

[66] U. B. Singh, D. Singh, S. Kumar, R. Dhar, M. B. Pandey, The optical properties of quantum dots in anisotropic media. J. Mol. Liq. 241, 1009–1012 (2017).

[67] S. Ramanathan, G. Petersen, K. Wijesundara, R. Thota, E. A. Stinaff, M. L. Kerfoot, M. Scheibner, A. S. Bracker, D. Gammon, Quantum-confined Stark effects in coupled InAs/GaAs quantum dots. Appl. Phys. Lett. 102, 213101 (2013).

[68] H. Ye, P. Lu, Z. Yu, B. Jia, H. Feng, Y. Liu, Equilibrium critical size of coherent InSb/GaSb quantum dot. Phys. E 42, 2402–2405 (2010).

[69] N. Baer, S. Schulz, P. Gartner, S. Schumacher, G. Czycholl, F. Jahnke, Influence of symmetry and Coulomb correlation effects on the optical properties of nitride quantum dots. Phys. Rev. B 76, 075310 (2007).

[70] X. Zhang, P. Sharma, Size dependency of strain in arbitrary shaped anisotropic embedded quantum dots due to nonlocal dispersive effects. Phys. Rev. B 72, 195345 (2005).

[71] G. Bastard, *Wave mechanics applied to semiconductor heterostructures*. (1990).

[72] D. L. Aronstein, C. R. Stroud Jr., General series solution for finite square-well energy levels for use in wave-packet studies. Am. J. Phys. 68, 943–949 (2000).

[73] A. L. Efros, L. E. Brus, Nanocrystal quantum dots: From discovery to modern development. ACS Nano 15, 6192–6210 (2021). 33830732 10.1021/acsnano.1c01399

[74] M. Wang, C. Li, F. Ke, Recurrent multi-level residual and global attention network for single image deraining. Neural Comput. Appl. 35, 3697–3708 (2023).

[75] S. Cheng, R. Chan, A. Du, CACFTNet: A hybrid cov-attention and cross-layer fusion transformer network for hyperspectral image classification. IEEE Trans. Geosci. Remote Sens. 62, 1–17 (2024).

[76] D. Hong, Z. Han, J. Yao, L. Gao, B. Zhang, A. Plaza, J. Chanussot, SpectralFormer: Rethinking hyperspectral image classification with transformers. IEEE Trans. Geosci. Remote Sens. 60, 1–15 (2022).

[77] D. Kohen, S. Bao, K. H. Lee, K. E. K. Lee, C. S. Tan, S. F. Yoon, E. A. Fitzgerald, The role of AsH3 partial pressure on anti-phase boundary in GaAs-on-Ge grown by MOCVD–Application to a 200mm GaAs virtual substrate. J. Cryst. Growth 421, 58–65 (2015).

[78] S. Lazzari, M. Abolhasani, K. F. Jensen, Modeling of the formation kinetics and size distribution evolution of II–VI quantum dots. React. Chem. Eng. 2, 567–576 (2017).

