# Supplementary material for: On-demand growth of semiconductor heterostructures guided by physics-informed machine learning
Source: Sci Adv. 2026 Jul 15;12(29):eaeb8867. doi: 10.1126/sciadv.aeb8867 (PMC13371887; doi:10.1126/sciadv.aeb8867)
Supplement: Supplementary file 1 — Notes S1 to S17 Figs. S1 to S28 Tables S1 and S2 Legends for movies S1 to S3 Legend for data S1 References [file sciadv.aeb8867_sm.pdf]

Science Advances
AAAS

Supplementary Materials for

On-demand growth of semiconductor heterostructures guided by physics-informed machine learning

Chao Shen *et al.*

Corresponding author: Chao Zhao, zhaochao@semi.ac.cn

Sci. Adv. **12**, eaeb8867 (2026)
DOI: 10.1126/sciadv.aeb8867

The PDF file includes:

Notes S1 to S17
Figs. S1 to S28
Tables S1 and S2
Legends for movies S1 to S3
Legend for data S1
References

Other Supplementary Material for this manuscript includes the following:

Movies S1 to S3
Data S1

Supplementary Text

Supplementary Note S1. The platform environment and program interface

As shown in Fig. S1, SemiEpi's basic functionalities include monitoring vacuum levels, controlling the cell shutter, and managing cell and substrate temperatures through the "Vacuum", "Cell Shutter", and "Cell and Substrate Temperatures" modules. It captures real-time RHEED data from the fluorescent screen using the "Camera Switch" module. The main features of SemiEpi include acquiring the substrate parameter initialization curve and self-optimizing QD growth parameters. During growth, the "Model Switch" module manages the ML model to process data, while the "Reminder Information" module displays the growth status.

In the "Functional Interface Switch" module, each operation is represented by a button. The interface provides continuous prompts and real-time processing results during model execution. During operation, data such as vacuum levels, temperatures, cell shutter states, model outputs, and setup parameters are stored in the cache as fixed-interval strings. This data is converted to Comma-Separated Values (.csv) files at least once per second, named based on the platform's start time, and is accessible for offline analysis. The high quality of these datasets is attributed to using variable-parameter material growth instead of traditional fixed-parameter methods.

Before operating SemiEpi, essential parameters such as thickness and growth rate are required for heterostructure design. The equipment control module executes commands from the parameter setting module, managing connected equipment and performing the multi-step process, including temperature initialization, material growth, and in-situ characterization. The material growth module monitors material status throughout the growth process.

Supplementary Note S2. The functional interface switch in the program

As shown in Fig. S2, when running SemiEpi, the first step is to obtain the parameter initialization curve using the theoretical and thermocouple temperature by the "Growth Temperature Initialization" module. This involves running sub-modules to find the deoxidation temperature automatically, the growth of GaAs, and then gather additional initialization temperature points, using "Deoxidation module", "GaAs growth module", and "Reconstruction module". The deoxidation temperature and the additional initialization points are analyzed using the "Initialization Model", whose output is shown in the "Initialization Model Output" module. Based on this data, curves are fitted to determine the initial growth temperatures for InAs and GaAs in the "Parameter initialization module".

SemiEpi performs growth automatically based on the structure design using the "InAs/GaAs QDs on GaAs Substrate" module. When executing the "Buried InAs QDs Growth" and "Surface InAs QDs Growth" modules, the substrate temperature is adjusted and optimized in real time based on the results shown in the "Temperature Model Output". If the display shows "Suitable", the current substrate temperature is maintained; if "High" is displayed, the temperature is lowered; and if "Low" is displayed, the temperature is raised. The completion of QD growth is assessed by the "Shutter Model Output". If "Yes" is displayed, the In shutter is closed immediately, signaling the completion of InAs QDs growth. With the sequential growth of buried InAs QDs, GaAs, and surface InAs QDs, the growth is completed.

Supplementary Note S3. The software and hardware configuration

The software and hardware configuration of the system are shown in Fig. S3. The heart of the process is software control that governs a multi-step growth recipe. This recipe executes critical phases sequentially, including substrate deoxidation, parameter initialization, and the actual

material growth. Throughout the deposition process, the system performs comprehensive data preprocessing. This primarily involves analyzing real-time RHEED patterns using a machine learning model. All acquired process data is logged and stored in a versatile Comma-Separated Values file format. The fundamental operation of the system is hardware control, which involves the precise orchestration of the MBE apparatus. This is accomplished using legacy communication protocols, such as RS232, to manage critical components, including shutters, cell temperatures, and substrate temperature. Additionally, it acquired the integrity of two separate vacuum environments: the growth vacuum and the buffer vacuum. A camera, connected via a high-speed USB 3.0 interface, is used to capture images of the fluorescent screen displaying the RHEED patterns.

Supplementary Note S4. The construction of the physics model

Conventional excitonic energy models typically simplify QDs as simple geometric shapes such as cubes or cylinders. However, these simplifications fail to accurately reflect the lens-shaped morphology that naturally arises during self-organized growth (*60*). More critically, such models neglect the anisotropy of the carrier wavefunction induced by curvature variations at the QD-substrate interface. To describe the QD more precisely, we construct an efficient model dealing with the ground-state transitions based on the eight-band envelope-function model in our earlier work, which incorporates dimension-coupled corrections, carrier-specific sensitivity, and surface scaling effects. Within this framework, we extract carrier-specific wavefunction responses and optimize excitonic energy predictions using effective size parameters, vertical-to-lateral coupling factors, and a universal size-dependent energy shift correction (*61*).

To better capture the geometry and size-dependent scaling of excitonic properties of lens-shaped QDs, we introduce a dual-parameter system that utilizes the lateral diameter $L_x = L_y = D$ and the vertical height $L_z = H$ of the QDs. This allows us to define the fundamental aspect ratio (AR) as (*62*):

$$AR = \frac{L_z}{L_x} = \frac{H}{D} \tag{S1}$$

However, employing only L_x, L_z, and AR as geometric parameters for predicting excitonic energies still results in notable discrepancies from the actual QD morphology. Traditional models that rely solely on the lateral diameter L_x tend to overestimate lateral confinement, primarily because they neglect the attenuation of the carrier wavefunction at the QD-substrate interface (*63*). This overestimation becomes increasingly significant as the QD dimensions shrink. To correct for this lateral confinement error, we introduce an exponential correction factor to define an effective lateral size, $L_{\text{eff},xy}$, as (*64*):

$$L_{\text{eff},xy} = L_x[1 - 0.2\exp(-\alpha_k \cdot \text{AR})] \tag{S2}$$

where α_k is the interface attenuation factor, characterizing wavefunction penetration depth. Similarly, conventional potential well models using the nominal height L_z significantly underestimate the curvature-enhanced localization of carriers (*65*). Therefore, to correct for this vertical confinement underestimation, we introduce a linear correction to obtain the effective vertical size, $L_{\text{eff},z}$, as (*66*):

$$L_{\text{eff},z} = L_z(\beta_0 + \beta_1 \cdot \text{AR}) \tag{S3}$$

where β_0 and β_1 represent the planar confinement baseline and the curvature enhancement coefficient.

Critically, the curvature of the lens-shaped QD disrupts the spatial symmetry assumed in simple geometries, leading to inter-dimensional coupling that is often overlooked by conventional

decoupled-dimension methods. To quantify this coupling, particularly the effect of vertical confinement on lateral wavefunctions, we introduce the strain transfer efficiency γ, which is the proportion of vertical strain that induces lateral deformation through Poisson's effect, to construct a vertical-to-lateral coupling factor f_z as follows:

$$f_z = 1 + \gamma \cdot \mathrm{AR} \tag{S4}$$

Additionally, this curvature-induced dimensional coupling affects electrons and holes differently due to their distinct effective masses. Treating both types of charge carriers with identical parameterization obscures the mass-dependent divergence in wavefunction responses to curvature (*67*). To address this, we adopt the sensitivity parameter approach proposed by Kabi et al (*39*)., incorporating wavevector magnitudes and mass-dependent sensitivity factors. This enables carrier-specific modeling of vertical confinement through the parameters $P_{e,z}$ and $P_{h,z}$, which represent the vertical sensitivity for electrons and holes, respectively:

$$P_{e,z} = \frac{\sqrt{2m_0 m_e^* V_{0e} e}}{\hbar} \cdot \frac{L_{\mathrm{eff},z}}{2}(1 + 0.3 \cdot \mathrm{AR}) \tag{S5}$$

$$P_{h,z} = \frac{\sqrt{2m_0 m_h^* V_{0h} e}}{\hbar} \cdot \frac{L_{\mathrm{eff},z}}{2}(1 + 0.25 \cdot \mathrm{AR}) \tag{S6}$$

Having established carrier-specific parameters for vertical confinement, we next address how this vertical influence couples into the lateral confinement. Implementing such a carrier-specific treatment effectively requires maintaining lateral rotational symmetry. However, cylindrical coordinates struggle to accommodate the lens-shaped geometry and the introduced coupling. Therefore, within a Cartesian framework, we extend the vertical-to-lateral coupling factor f_z into the lateral directions for both electrons and holes (*68-70*):

$$P_{e,x} = P_{e,y} = \frac{\sqrt{2m_0 m_e^* V_{0e} e}}{\hbar} \cdot \frac{L_{\mathrm{eff},xy}}{2} \cdot f_z \tag{S7}$$

$$P_{h,x} = P_{h,y} = \frac{\sqrt{2m_0 m_h^* V_{0h} e}}{\hbar} \cdot \frac{L_{\mathrm{eff},xy}}{2} \cdot f_z \tag{S8}$$

Having defined $L_{\mathrm{eff},xy}$, $L_{\mathrm{eff},z}$, f_z, and the carrier-specific sensitivity parameters $P_{e,i}$ and $P_{h,i}$ across all three spatial dimensions, we are now equipped to calculate the confinement energies. At the nanoscale, quantum tunneling through the finite potential barriers becomes significant. Models based on infinite potential wells tend to greatly overestimate confinement energies, while first-order perturbation theory is often insufficiently accurate. To achieve a balance between accuracy and computational efficiency, and to account for this finite barrier effect within our established parameter framework, we adopt a third-order expansion of the confinement energies defined by Kabi et al (*39*). for each confinement direction and carrier type (*39,71,72*):

$$E_{e,i} = \frac{2P_{e,i}^2}{(P_{e,i}+1)^2}\left[\left(\frac{\pi}{2}\right)^2 - \frac{1}{3(P_{e,i}+1)^3}\left(\frac{\pi}{2}\right)^4 - \frac{27P_{e,i}-8}{180(P_{e,i}+1)^6}\left(\frac{\pi}{2}\right)^6\right]\frac{\hbar^2}{m_e^* m_0 L_{\mathrm{eff},i}^2 e} \tag{S9}$$

$$E_{h,i} = \frac{2P_{h,i}^2}{\left(P_{h,i}+1\right)^2}\left[\left(\frac{\pi}{2}\right)^2 - \frac{1}{3\left(P_{h,i}+1\right)^3}\left(\frac{\pi}{2}\right)^4 - \frac{27P_{h,i}-8}{180\left(P_{h,i}+1\right)^6}\left(\frac{\pi}{2}\right)^6\right]\frac{\hbar^2}{m_h^* m_0 L_{\mathrm{eff},i}^2 e} \tag{S10}$$

Thus, the total quantum confinement energy E_{conf} is obtained by summing the contributions from all three spatial dimensions and both carrier types:

$$E_{\mathrm{conf}} = \sum_{i=x,y,z}\left(E_{e,i} + E_{h,i}\right) \tag{S11}$$

For smaller QDs, contributions from surface atoms become dominant, rendering purely geometric confinement models inadequate. To address this surface-dominated regime, we introduce a universal correction term, ΔE_{size}, based on volume-equivalent scaling. This correction begins by calculating the equivalent side length L_{eq} of a cube with the same volume as the lens-shaped QD:

$$L_{\text{eq}} = (L_x L_y L_z)^{1/3} \tag{S12}$$

The size correction energy is then given by:

$$\Delta E_{\text{size}} = \frac{\alpha}{L_{\text{eq}}^{\beta}} \tag{S13}$$

where α and β represent the surface energy density and the scaling exponent.
Ultimately, the total exciton energy E_{total} for the InAs QD system, integrates all the contributions described above (*73*):

$$E_{\text{total}} = E_g + \delta + E_{\text{conf}} + \Delta E_{\text{size}} \tag{S14}$$

where E_g is the bulk band gap of InAs. δ is an empirical offset accounting for other minor effects.

Supplementary Note S5. The performance of the CatBoost model

We conducted a performance evaluation of the trained CatBoost model. In Fig. S4A, we present a scatter plot comparing the actual values to the predicted values. The data points are closely clustered along the diagonal line, which demonstrates the accuracy of the model's inference. Fig. S4B shows the distribution of prediction errors through a residual histogram. The residuals range from -2×10^{10} to 2×10^{10}, with the majority concentrated between -5×10^{9} and 5×10^{9}.
We further analyzed the impact of various inputs on density inference. Partial Dependence Plots (PDPs) were employed to visualize the marginal effect of individual features on the density inference results. To ensure features with different scales were treated fairly by the model, feature standardization was performed. Consequently, negative values on the x-axis do not represent physically negative dimensions; instead, they signify that the parameter value is lower than the average value across the entire training set. Fig. S5A illustrates the relationship between the "Height mean" and density inference. The model outputs show an overall trend of increasing and then decreasing as the "mean height" increases.
As the "Height std" increases, the model output exhibits an oscillating downward trend, as shown in Fig. S5B. This indicates that the density inference results are most accurate when the height distribution is moderate. Conversely, excessive dispersion or concentration in height adversely impacts the model's inference results.
As the "Diameter mean" increases, the inference values exhibit a decreasing trend, as shown in Fig. S5C. When the "Diameter mean" reaches a high value, the model's inference value falls into the negative region, suggesting that QDs with smaller diameters may yield higher inference density values.
Fig. S5D shows the impact of "Diameter Std" on the density inference results. As the "Diameter Std" increases, the model's inference results display an upward trend. Moreover, when the standard deviation is close to zero, the inference results show the least fluctuation in density inference, indicating that a more centralized size distribution of QDs leads to more accurate model inferences.

Supplementary Note S6. The comparison results of different regression models

The performance of different regression models, including R-squared value (R^2), mean absolute error (MAE), training time, and inference time, is detailed in Fig. S6. The Random Forest model achieved optimal prediction performance, with an R^2 of 0.83 and an MAE of 6.7×10^9. However, its inference latency reached 6.3 ms. In contrast, simpler models, such as Linear Regression, demonstrated rapid training and inference but lacked sufficient accuracy.
CatBoost has slightly less accuracy than the best model, but it has a higher R^2 of 0.78 and a lower MAE of 7.0×10^9. Although each epoch takes 1.65 seconds to train, the inference time is 1.15 ms, which is longer than some other models but significantly shorter than the material growth cycle. Therefore, CatBoost is an ideal solution for real-time decision-making systems.

Supplementary Note S7. Detailed information of 30 samples
The 30 InAs/GaAs QD samples were grown under a wide range of substrate temperatures spanning from 453 °C to 501 °C. The photoluminescence (PL) intensity ranges from lower values up to a peak of 3724 arb. units, while the full width at half maximum (FWHM) spans from a highly uniform 27.3 meV to a broader 80.6 meV. Additionally, the corresponding QD densities span an order of magnitude, from 1.28×10^{10} cm^{-2} to 1.30×10^{11} cm^{-2}, as shown in Table S2.

Supplementary Note S8. The structure of the GARN and CAF blocks
The Initialization Model and Growth Models are constructed using GARN block, CAF block, and multilayer perceptron (MLP). GARN block first maps input features to a reduced number of channels through convolutional layers, as shown in Fig. S7A (*74*). Several generalized attention residual blocks follow this, each comprising two convolutional layers and a channel attention mechanism which adjusts the contribution of each channel by globally pooling the feature map and calculating importance weights. Finally, a downsampling layer and an additional convolutional layer further process the feature map, reducing its spatial resolution to generate the final output, which is a three-dimensional array. The CAF block leverages a transformer-based architecture to process image data, as shown in Fig. S7B (*75*). The input of a three-dimensional array from GARN is divided into small blocks, and their features are embedded through linear transformations. The transformer's attention mechanism captures global dependencies between image blocks and processes cross-layer associations. Traditional CNN can lead to the loss of vital angle-dependent diffraction details. In contrast, the CAF mechanism excels at processing RHEED images by dynamically adjusting feature channel weights, where essential diffraction information shifts at various angles due to substrate rotation and wobbling. This mechanism allows the CAF to effectively model contextual relationships between different image blocks, enhancing the recognition of complex patterns (*76*). The model's feed-forward network further processes these features, which are finally classified using a MLP. This adjustment enables the GARN and CAF blocks to identify and extract critical features more accurately, a crucial capability for detecting subtle feature changes in images captured at varying angles.

Supplementary Note S9. The comparison of GARN-CAF architecture with other models
To verify the necessity of the model architecture, we compared the performance of GARN-CAF with Vision Transformer (ViT) as well as three-dimensional Convolutional Neural Network (3D-CNN) and Convolutional Long Short-Term Memory (ConvLSTM) models. The experimental results in Fig. S8 show that GARN-CAF achieves the highest accuracy across all growth stages. In the reconstruction task, the validation accuracy of GARN-CAF reached 92.37%, which is

significantly better than the 71.44% of ConvLSTM and 59.96% of 3D-CNN. For the temperature model, our architecture achieved an accuracy of 92.43% while other models remained below 77%. The validation accuracy of the shutter model also remained at a high level of 91.43%.

Supplementary Note S10. The comparison results from different function fits

Selecting an appropriate fitting function is crucial to achieving better curve-fitting results. The thermocouple temperatures of 134 °C, 360 °C, 435 °C, and 484 °C used for curve fitting corresponded to the theoretical temperatures of 350 °C, 510 °C, 580 °C, and 620 °C. The fitting results using primary, secondary, and tertiary functions were plotted, as shown in Fig. S9, and the mean squared error (MSE) values were 35.2, 4.6, and 0.0, respectively. Although the cubic function provided the lowest MSE, it exhibited significant fluctuations between data points due to the limited number of data points or their uneven distribution, which could lead to overfitting. Therefore, considering the balance between fit accuracy and stability, the quadratic function was chosen as the optimal fitting function.

Supplementary Note S11. The surface morphology and crystal quality of GaAs grown at different temperatures

Research indicates that the optimal growth temperature for GaAs is 600 °C in MBE. Fig. S10 shows the surface morphology of GaAs grown at different temperatures and their corresponding X-ray diffraction (XRD) results. AFM and XRD analysis of GaAs grown at temperatures of 570 °C, 600 °C, and 615 °C consistently suggests a high epitaxial quality of GaAs on the substrate. At the same scale bar, as the GaAs growth temperature increases, the sample morphology transitions from showing distinct bright and dark variations, indicating an uneven surface with characteristic anisotropic mounds, to a progressively flatter surface. It should be noted that these elongated surface features are intrinsic physical characteristics of GaAs (001) homoepitaxy rather than imaging or scan artifacts; they arise from the inherent anisotropy in adatom diffusion lengths along different crystallographic directions during the MBE process (*77*).

By analyzing the full width at half maximum (FWHM) from X-ray diffraction (XRD) data, it is observed that the FWHM values are lower and exhibit only minor fluctuations from 570 °C to 615 °C, as shown in Fig. S11. The sample grown at 600 °C shows the narrowest FWHM, indicating an optimal lattice match between the epitaxial GaAs layer and the substrate.

Supplementary Note S12. The variation in QD density with thermocouple temperature

The observed inverse proportion between QD density and temperature is a direct manifestation of temperature-dependent surface kinetics. In the Stranski-Krastanov (S-K) growth mode, the initial nucleation site density is determined by the surface strain field and temperature: lower temperatures restrict adatom diffusion, favoring high nucleation rates that yield high-density, small-sized QDs, whereas enhanced surface mobility at higher temperatures suppresses nucleation, producing low-density, large-sized QDs. Following QD formation, temperature-driven Ostwald ripening process occurs, where enhanced adatom diffusion facilitates island coalescence and the dissolution of smaller islands to feed larger ones, resulting in a decrease in density and an increase in size—a distinct inverse proportion. This aligns with aggregation dynamics models such as the Smoluchowski equation, which describes the time evolution of island size distributions (*78*). Specially, Fig. S12 shows the QD density variation with changes from -5 °C to +5 °C around the target thermocouple temperature. Specifically, Figs. S12A–S12G

show that the QD density decreases from 8.5×10^{10} cm^{-2} at 5 °C below the target temperature to 2.39×10^{10} cm^{-2} at 5 °C above the target temperature. Fitting this temperature-dependent trend, it is evident that for every 1°C decrease in thermocouple temperature, the QD density increases by approximately 8×10^{9} cm^{-2}, as shown in Fig. S12H.

To achieve a QD density range of 4×10^{10} cm^{-2} to 6×10^{10} cm$^{-2,}$ the thermocouple temperature needs to be reduced by approximately 3.73 °C. Therefore, precisely controlling the thermocouple temperature within a suitable range, specifically within ±2 °C, is critical for ensuring consistent QD density within the desired range.

Supplementary Note S13. The reference sample prepared using conventional methods

To effectively demonstrate the advantages of SemiEpi, we manually grew a sample with the same structure as the one used in SemiEpi. The sample was deoxidized at 410 °C, the GaAs growth temperature was set to 421 °C, and the InAs QDs were grown at a reference temperature of 340 °C. The growth results were analyzed using AFM and PL characterization. The QD density was 3.7×10^{10} cm^{-2}, as shown in Fig. S13A. The sample also achieved a relative intensity of 2270 and a full width at half maximum (FWHM) of 34.70 meV, as shown in Fig. S13B.

Supplementary Note S14. The reference sample prepared using parameter initialization

This study includes an additional experiment to effectively highlight the advantages of the SemiEpi platform: growing a sample using the parameter initialization result and then performing the subsequent growth process manually.

Real-time data recorded by SemiEpi around different transition points was analyzed. During the automatic deoxidation process, SemiEpi heated up in increments of 5 °C, from 390 °C to 415 °C through five increments, as shown in Fig. S14A. The RHEED screen showed no distinctive features until the thermocouple temperature of 415 °C, when distinct bright spot features appeared, as shown in Fig. S14C and S14D. Analysis of the "Initialization Model" output during deoxidation revealed that, from the 0th to around the 16,000th sequence, the oxidation probability remained close to 1, indicating that the model did not identify the deoxidation state. After the 16,000th sequence, the deoxidation probability rapidly increased to nearly 1, confirming the model's accurate identification of the deoxidation state, as shown in Fig. S14B. After growing a layer of GaAs on the deoxidized sample and cooling, the substrate was gradually heated at a rate of 15 °C per minute, as shown in Fig. S14E. Real-time RHEED data collected during this process was analyzed using the "Initialization Model" to monitor reconstruction states, as shown in Fig. S14F. Initially, the model identified only "As cap" labels, with RHEED patterns showing diffuse features from the beginning to around the 2,000th sequence, as shown in Fig. S14G and S14H. By around the 3,000th sequence, at a thermocouple temperature at 135 °C, a marked shift occurred as the probability of "As cap" labels dropped sharply and "c(4×4)" labels became dominant. The RHEED patterns began exhibiting periodic features consistent with the emergence of a ×4 reconstruction, as shown in Fig. S14I and S14J. At around the 11,000th sequence, as the substrate heating reached 354°C, the probability of "(2×4)" labels increased significantly. This shift indicated a structural change on the surface, supported by RHEED observations of features combining ×2 and ×4, as shown in Fig. S14K and S14L. Finally, by the 15,000th sequence, the model output showed an increased probability for the "(n×6)" label. At this stage, the thermocouple temperature reached 476 °C, and the RHEED patterns displayed a distinct ×6 periodicity alongside residual ×2 features, as shown in Fig.

S14M and S14N. The results demonstrate the model's high sensitivity in distinguishing between different structural states.
Finally, SemiEpi used a quadratic curve to fit the collected thermocouple temperature data to inferred temperature data. This set the initial thermocouple temperatures for InAs growth at 322 °C and for GaAs growth at 449 °C, as shown in Fig. S14O.
After manually growing a sample at the above temperature, the growth results were analyzed by AFM. The density of the sample was 5.4×10^{10} cm^{-2}, as shown in Fig. S15A. Additionally, the sample achieved a relative intensity of 2531 and FWHM of 30.79 meV, as shown in Fig. S15B.

Supplementary Note S15. The time-resolved photoluminescence results

Time-resolved photoluminescence (TRPL) measurements were conducted at 130 K to assess the quantity of InAs QDs. Carrier lifetimes were extracted by fitting the TRPL data using a bi-exponential decay model. As illustrated in Fig. S16, the sample prepared using SemiEpi exhibits a lifetime of 2.06 ns, representing a significant improvement over the reference sample prepared using conventional methods, which has a lifetime of 1.77 ns. Additionally, the reference sample prepared using parameter initialization shows a lifetime of 2.04 ns. Our results indicate that the optimized QDs are of high quality, demonstrating the effectiveness of SemiEpi for material growth.

Supplementary Note S16. The five additional samples prepared with SemiEpi

To further validate the robustness of the SemiEpi platform, we conducted five additional growth experiments, the detailed results of which are shown in Fig. S17-S26. Movie S2 provides a real-time recording of the experimental growth process corresponding to the data presented in Figures S25–S26. Although the initialization module suggested varying starting temperatures of 331 °C, 309 °C, 291 °C, 299 °C, and 313 °C, respectively, the Growth Model continuously refined these temperatures during growth. Specifically, the substrate temperatures were optimized to 330 °C, 319 °C, 296 °C, 310 °C, and 317 °C for the growth of buried QDs, and further tuned to 330 °C, 321 °C, 296 °C, 309 °C, and 319 °C for the surface QDs. Additionally, the data clearly demonstrates that the shutter closure timing is highly dynamic: for the buried QDs, the termination sequences occurred at 1224, 1385, 1432, 1506, and 1395, while for the surface QDs, the timings were 1232, 1416, 1393, 1507, and 1408, respectively. The measured quantum dot (QD) densities for these five samples were 4.57×10^{10} cm^{-2}, 5.63×10^{10} cm^{-2}, 4.29×10^{10} cm^{-2}, 4.37×10^{10} cm^{-2}, and 5.88×10^{10} cm^{-2}, respectively. Their corresponding PL emission wavelengths were 1236 nm, 1245 nm, 1235 nm, 1241 nm, and 1242 nm. Notably, all prepared samples exhibited emission wavelengths closely aligned with the 1240 nm target, and the QD densities remained strictly within the prescribed target range of $4\text{-}6\times10^{10}$ cm^{-2}. These results demonstrate that SemiEpi effectively compensates for initial parameter fluctuations, ensuring highly reproducible and on-demand growth outcomes.

Supplementary Note S17. The additional samples prepared with SemiEpi on another MBE reactor

We also conducted additional growth experiments on another MBE reactor, the detailed results of which are shown in Fig. S27-S28 and the corresponding Movie S3. The initialization module accurately identified the critical surface transition temperatures for this specific equipment: deoxidation at 575 °C, the As-cap to c(4×4) at 122°C, the c(4×4) to (2×4) transition at 497 °C, and the (2×4) to (n×6) transition at 634°C. Based on these calibrated benchmarks, the system automatically established the initial growth temperatures at 462 °C for InAs QDs and 607 °C for the GaAs layer. Throughout the deposition process, the Temperature Model continuously analyzed the

surface kinetics and dynamically adjusted the substrate temperature; consequently, the buried QD growth was optimized to 470 °C, and the surface QDs were also regulated to a final temperature of 470°C. The resulting sample achieved a PL wavelength of 1241 nm and a surface QD density of 4.88 $\times 10^{10}$ cm^{-2}, both of which are in excellent agreement with the target specifications.

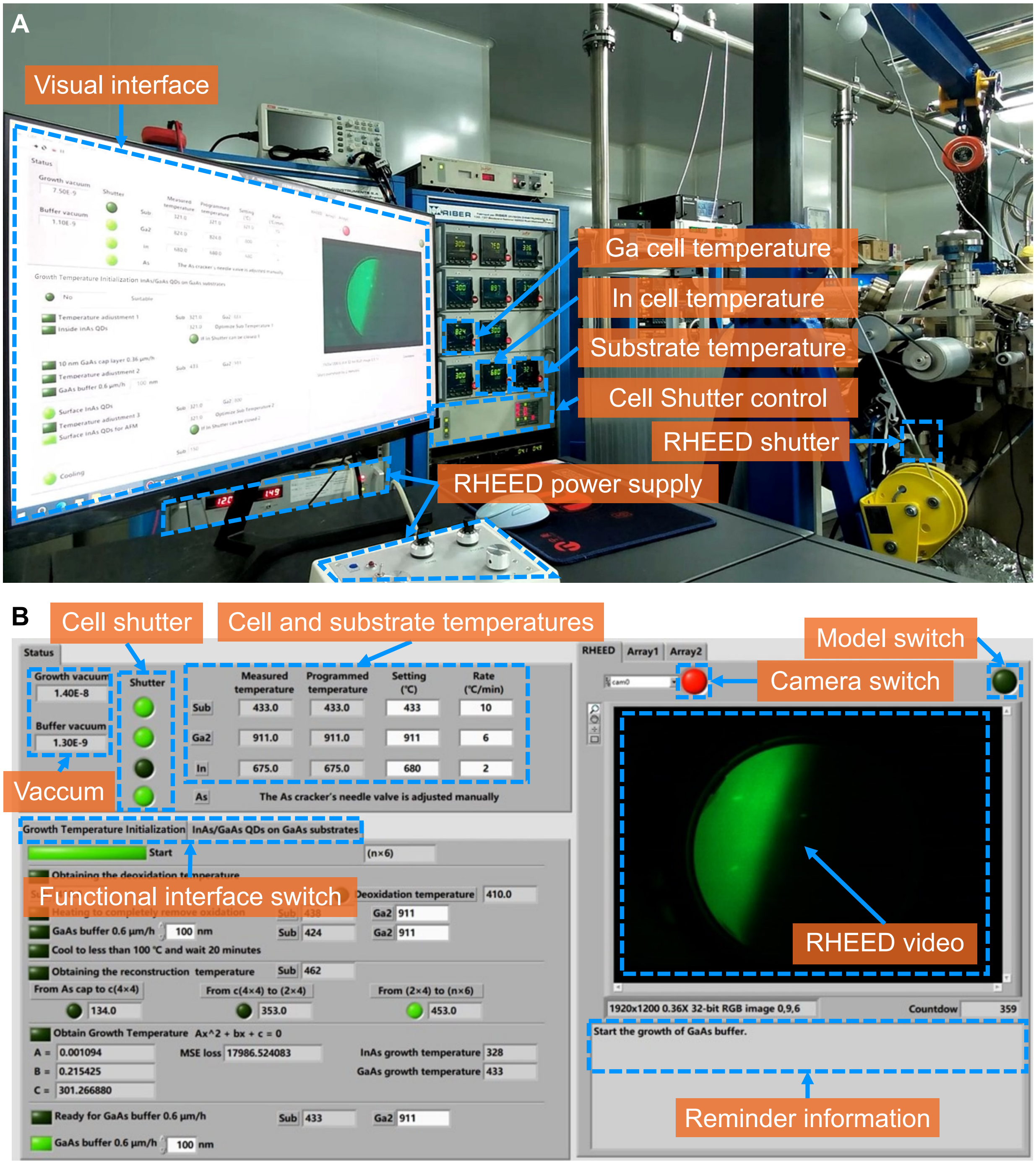

Fig. S1.
Platform environment and program interface. (A) Platform environment and (B) program interface.

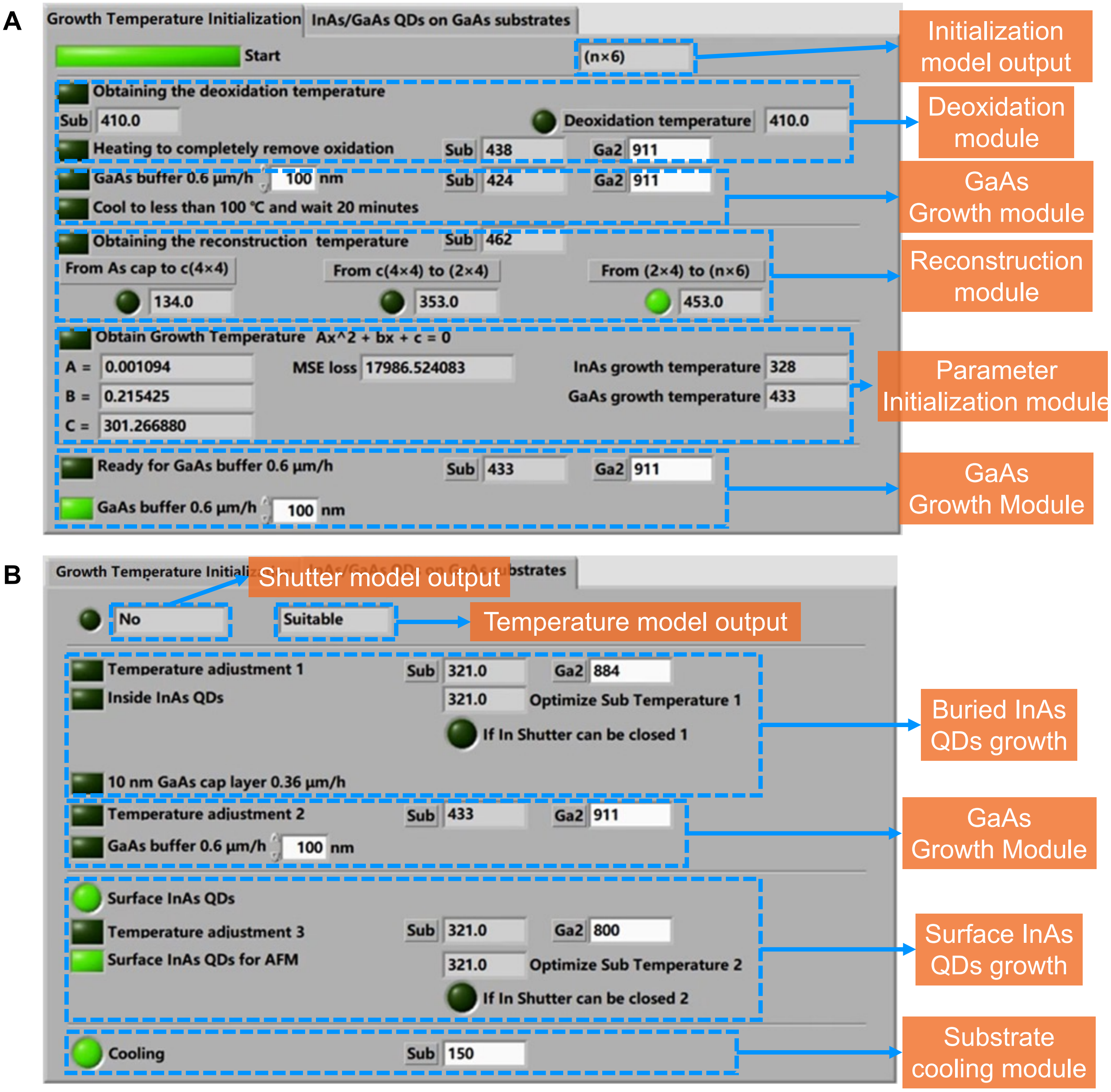

Fig. S2.

The functional interface switch in the program. (A) Parameter initialization. (B) InAs QDs growth.

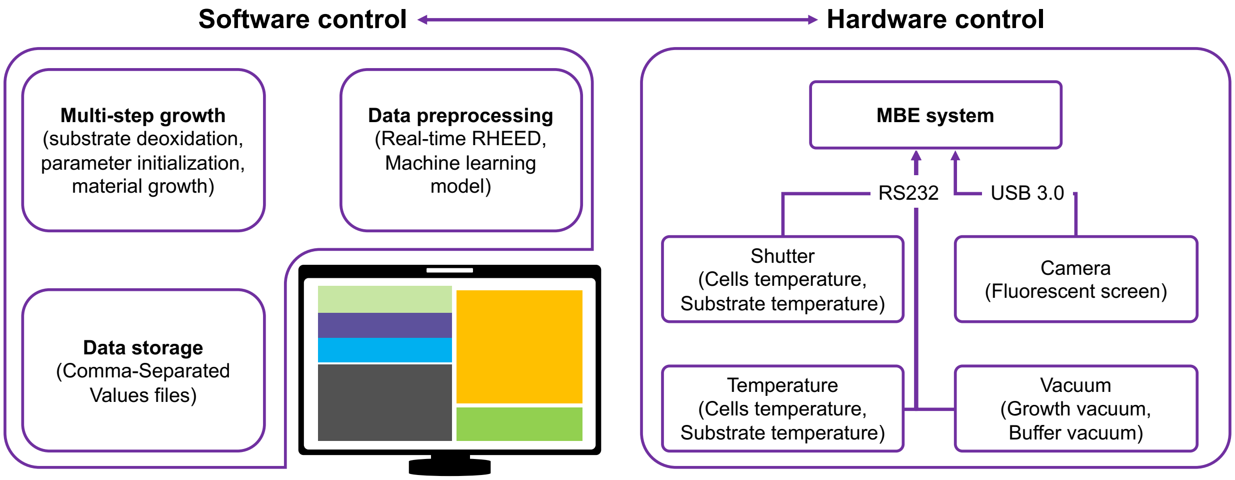

Fig. S3.

The software and hardware configuration of the system.

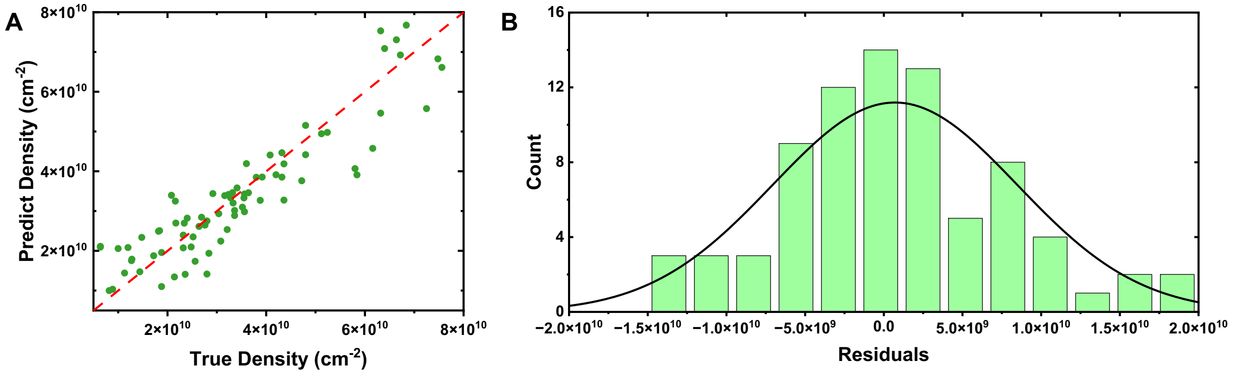

Fig. S4.

The performance of CatBoost model. (A) The actual and predicted plot. (B) The normal distribution of residuals.

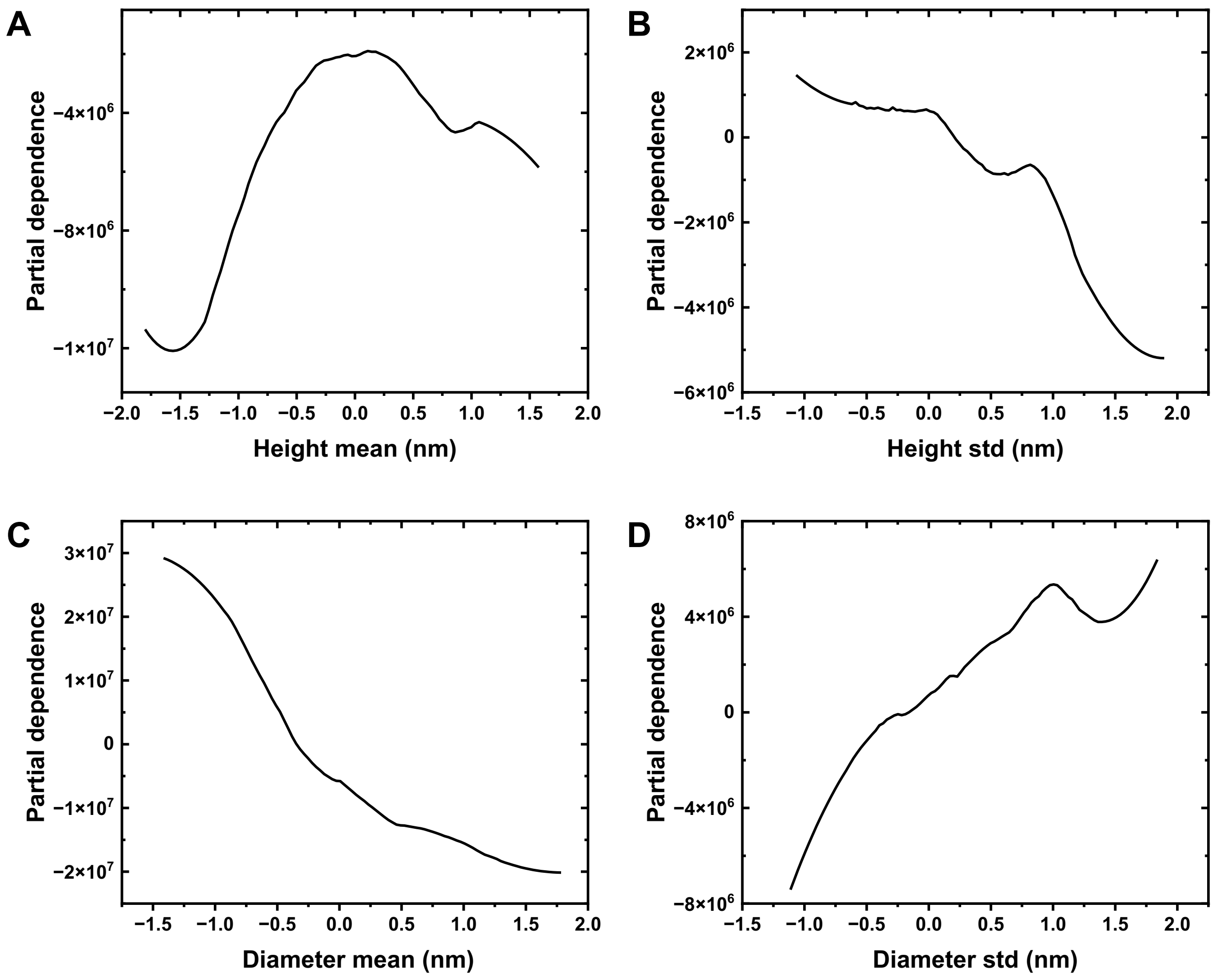

Fig. S5.

The partial dependence of the QDs size. (A) Height mean. (B) Height standard deviation (std). (C) Diameter mean. (D) Diameter std.

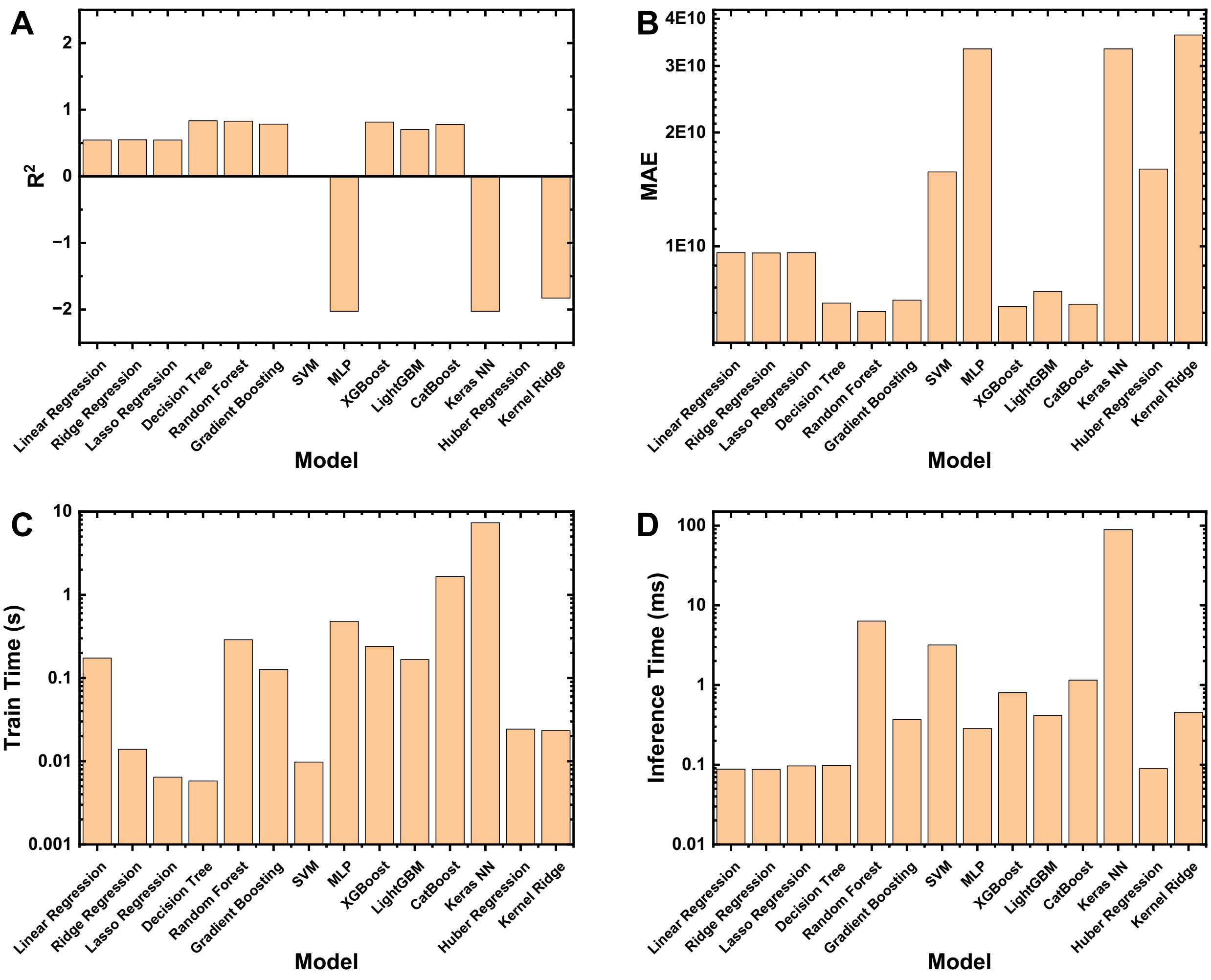

Fig. S6.

Performance comparison of different regression models. (A) R-squared (R2). (B) Mean absolute error (MAE). (C) Training time. (D) Inference time.

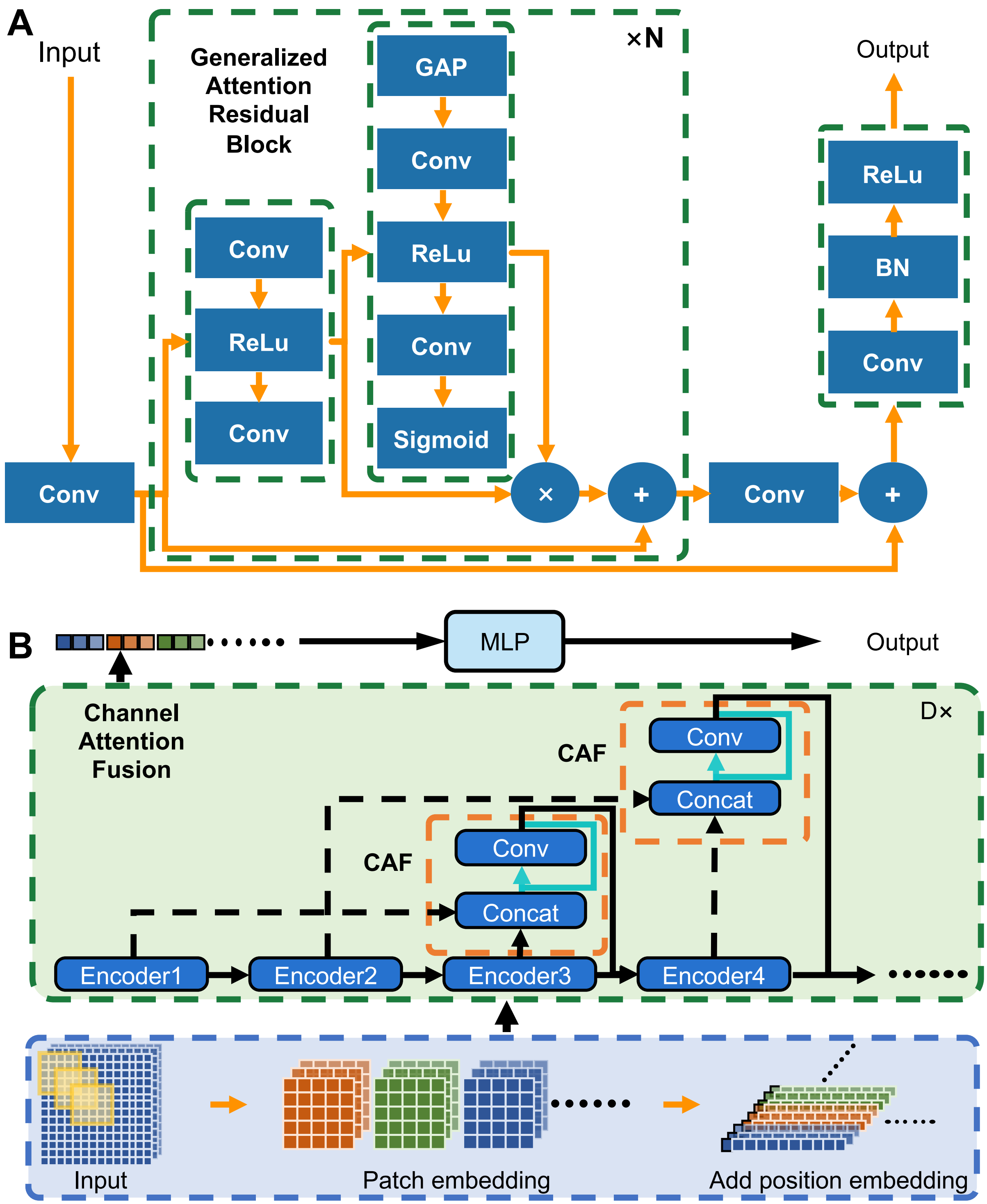

Fig. S7.

The structure of the GARN and CFA blocks. (A) GARN block. (B) CFA block. Conv: convolutional layer. ReLu: rectified linear unit activation layer. GAP: global average pooling layer. Sigmoid: sigmoid activation layer. BN: batch normalization layer. Encoder: transformer encoder. MLP: multi-layer perceptron.

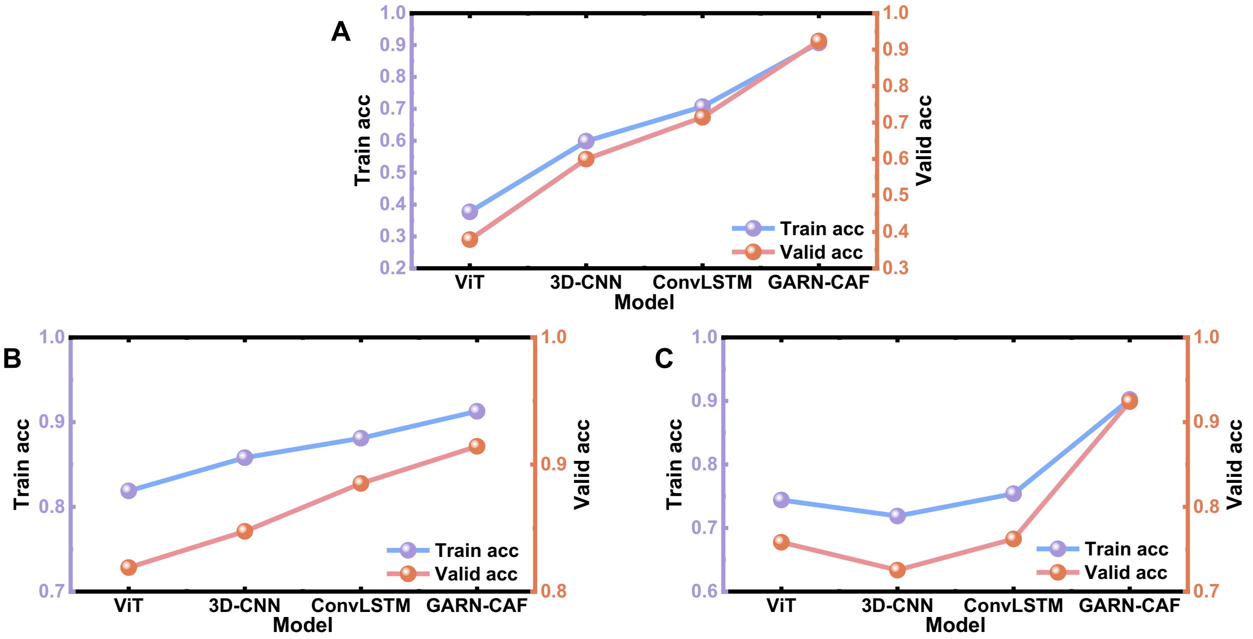

Fig. S8.

Comparison of the proposed GARN-CAF architecture with other models. (A) Reconstruction model task, (B) Shutter model task, and (C) Temperature model task.

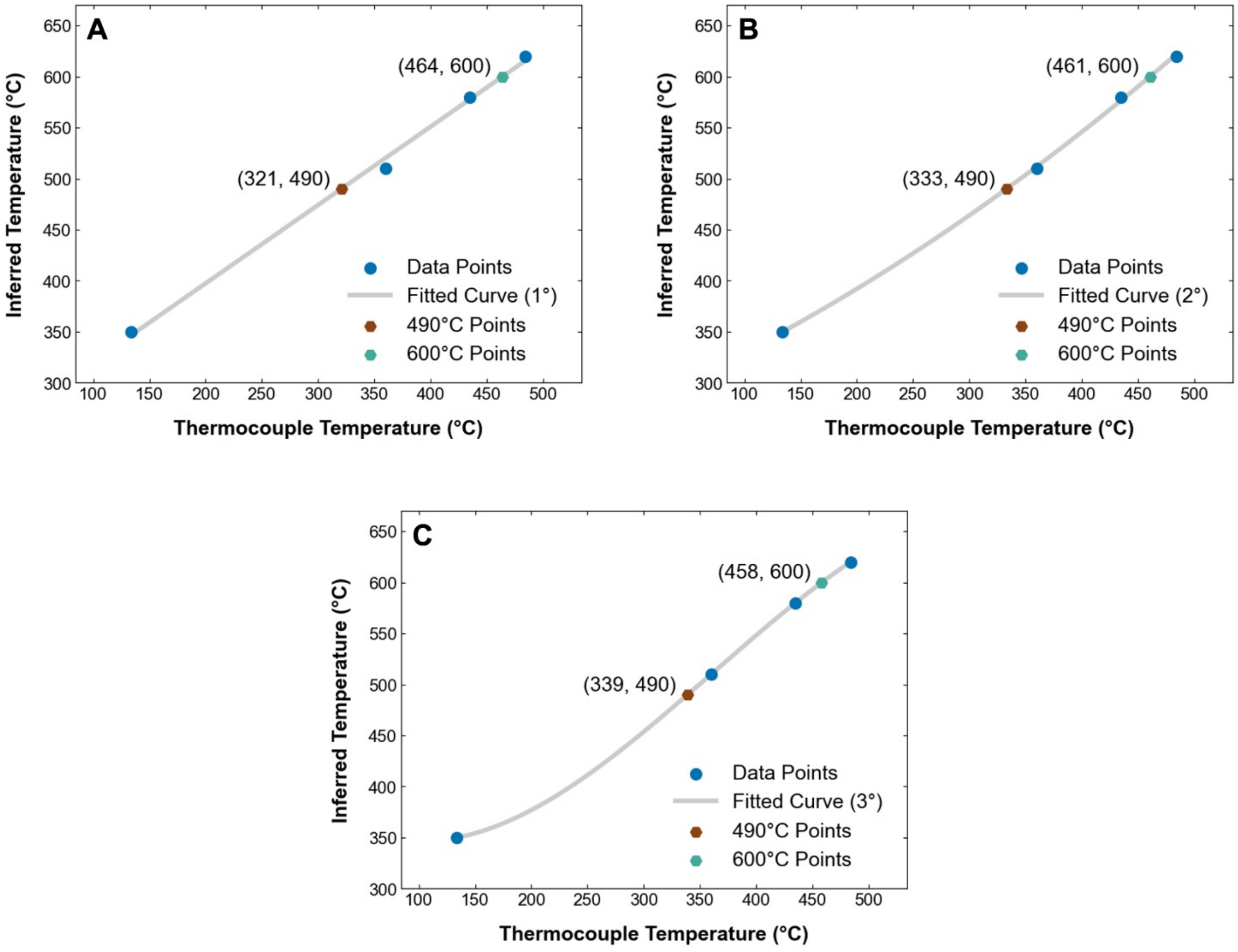

Fig. S9.

Comparison Results from Different Function Fits. (A) Primary function, (B) Quadratic function, (C) Cubic function.

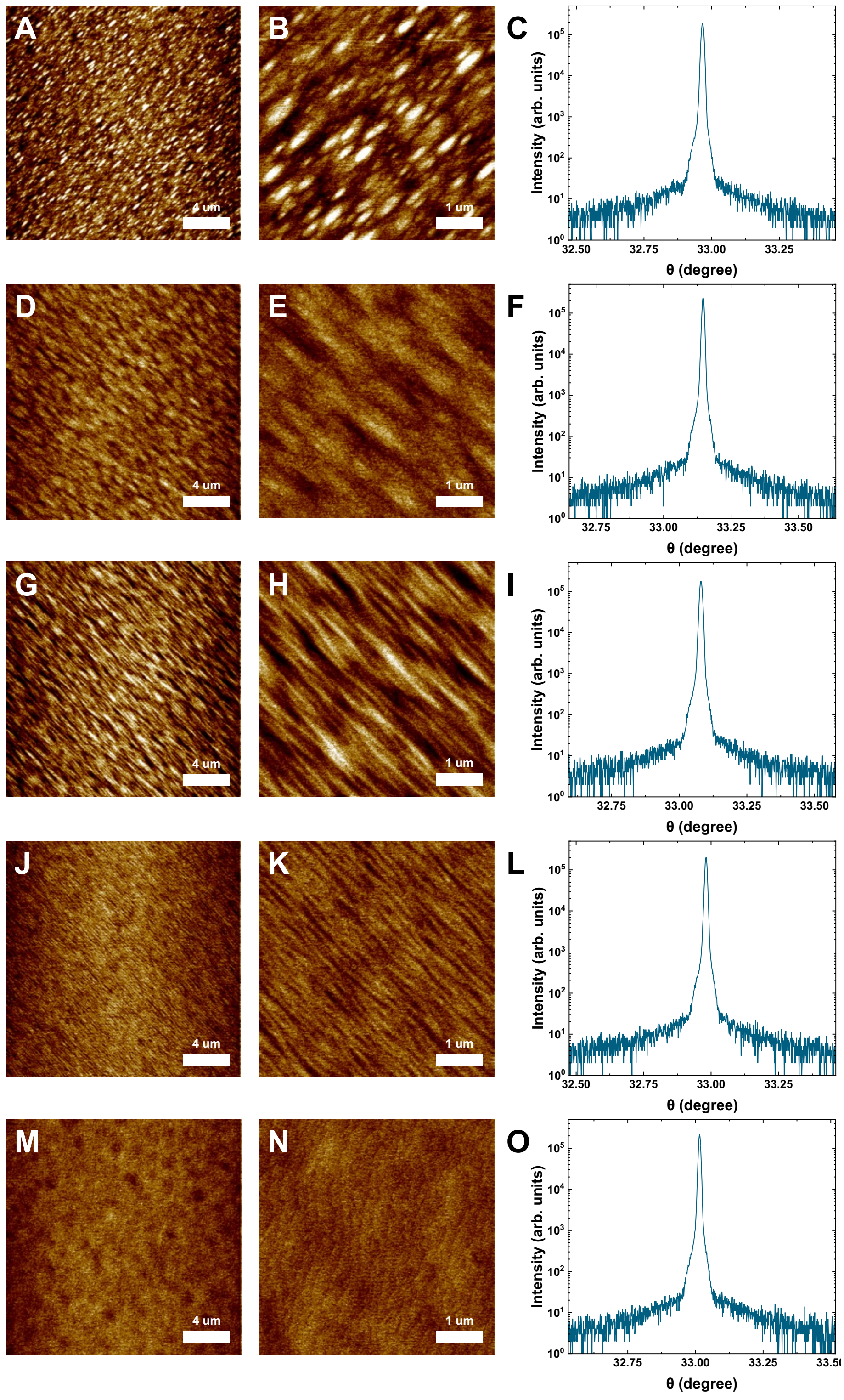

Fig. S10.

Surface morphology and crystal quality of GaAs grown at different temperatures. 20 μm and 5 μm AFM and XRD results of GaAs grown at (A-C) 540 °C, (D-F) 570 °C, (G-I) 600 °C, (J-L) 615 °C, (M-O) 630 °C.

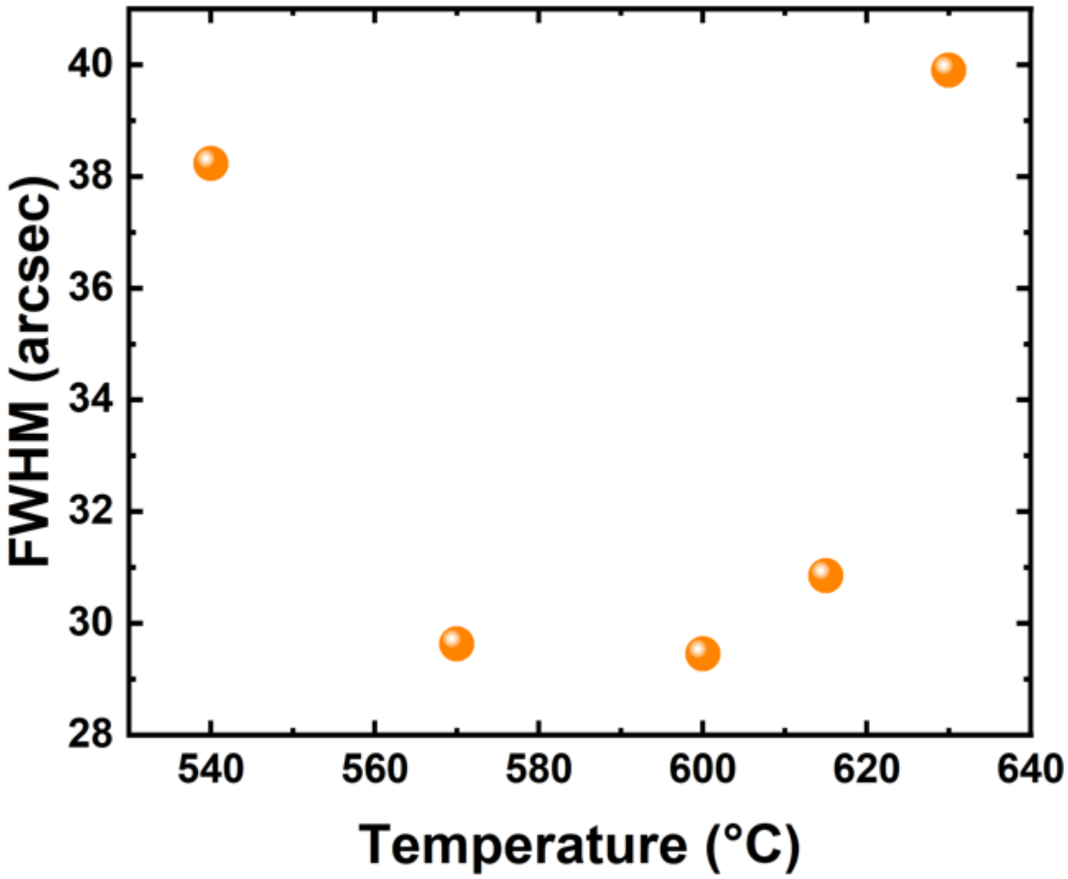

Fig. S11.
Statistical results of XRD FWHM for GaAs grown at different temperatures.

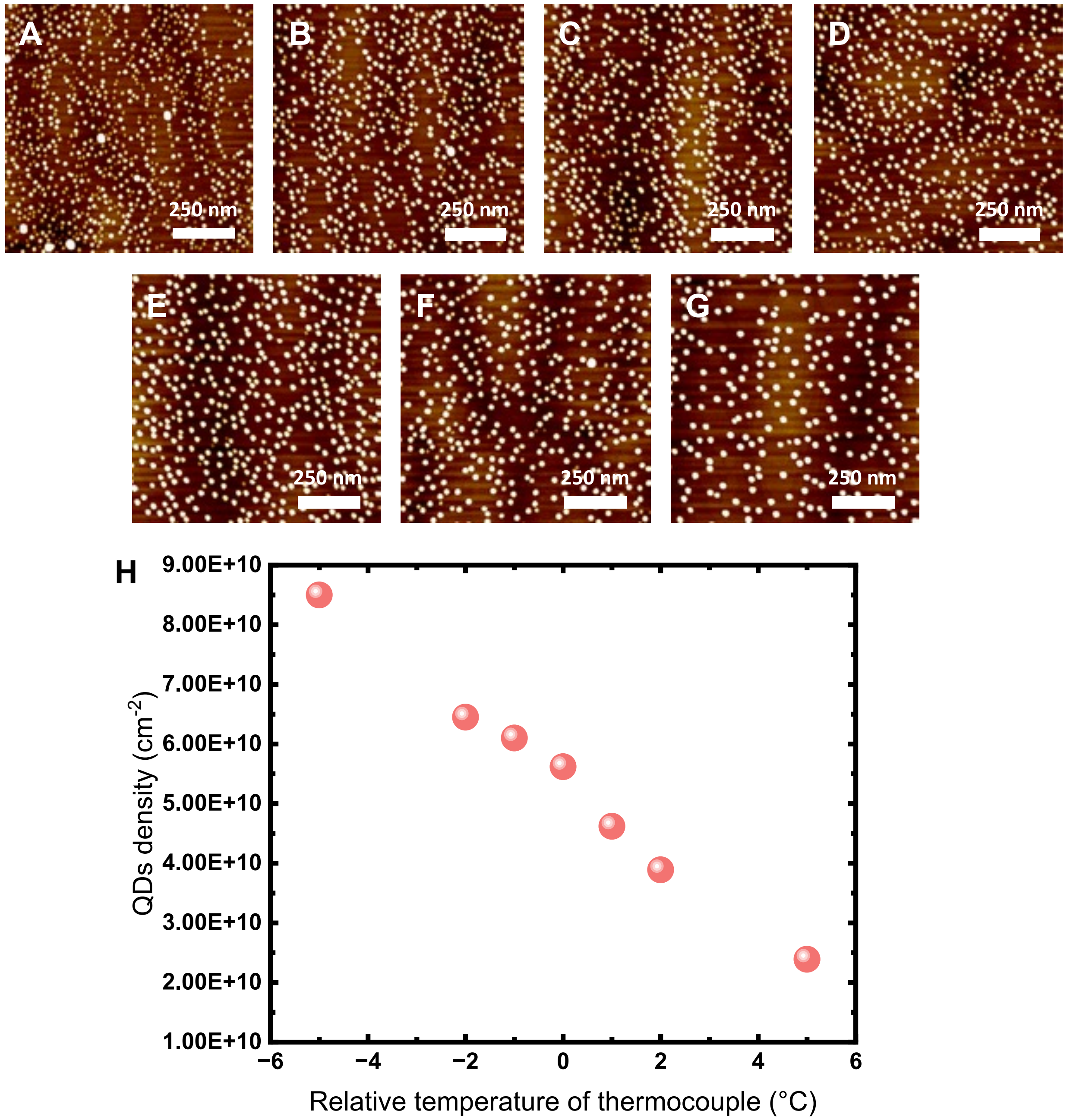

Fig. S12.

Variation of QD density with thermocouple temperature. (A-G) AFM images of QDs at different thermocouple temperatures, (H) a statistical graph showing the variation in QD density.

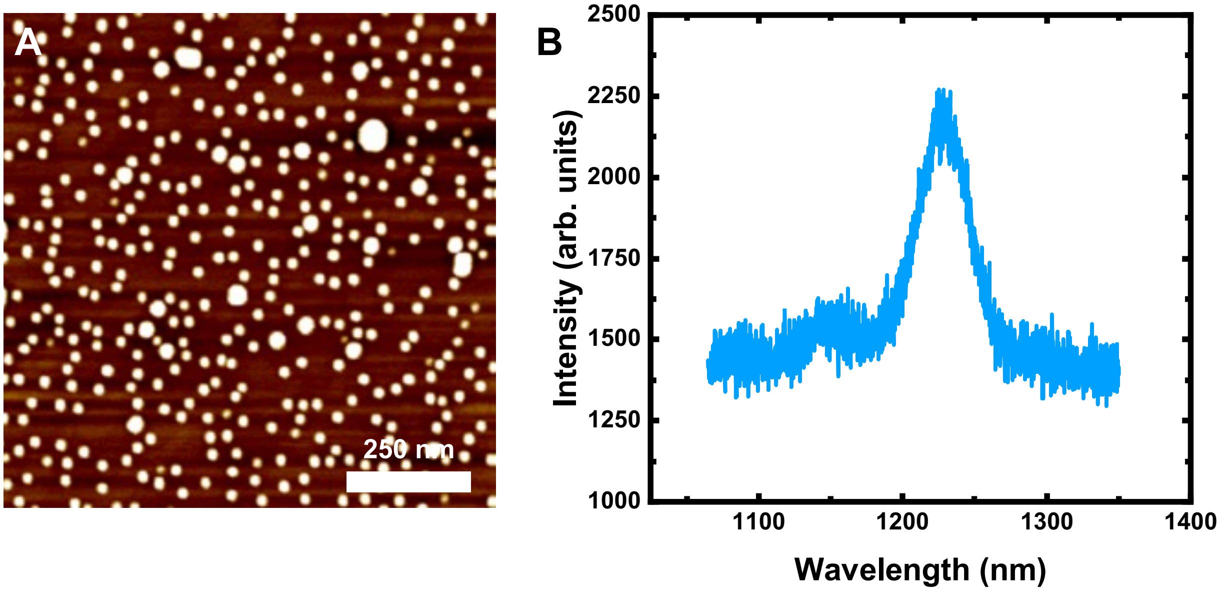

Fig. S13.

The reference sample prepared using conventional methods. (A) The 1 µm × 1 µm AFM image of the reference sample. (B) Photoluminescence spectrum of QDs.

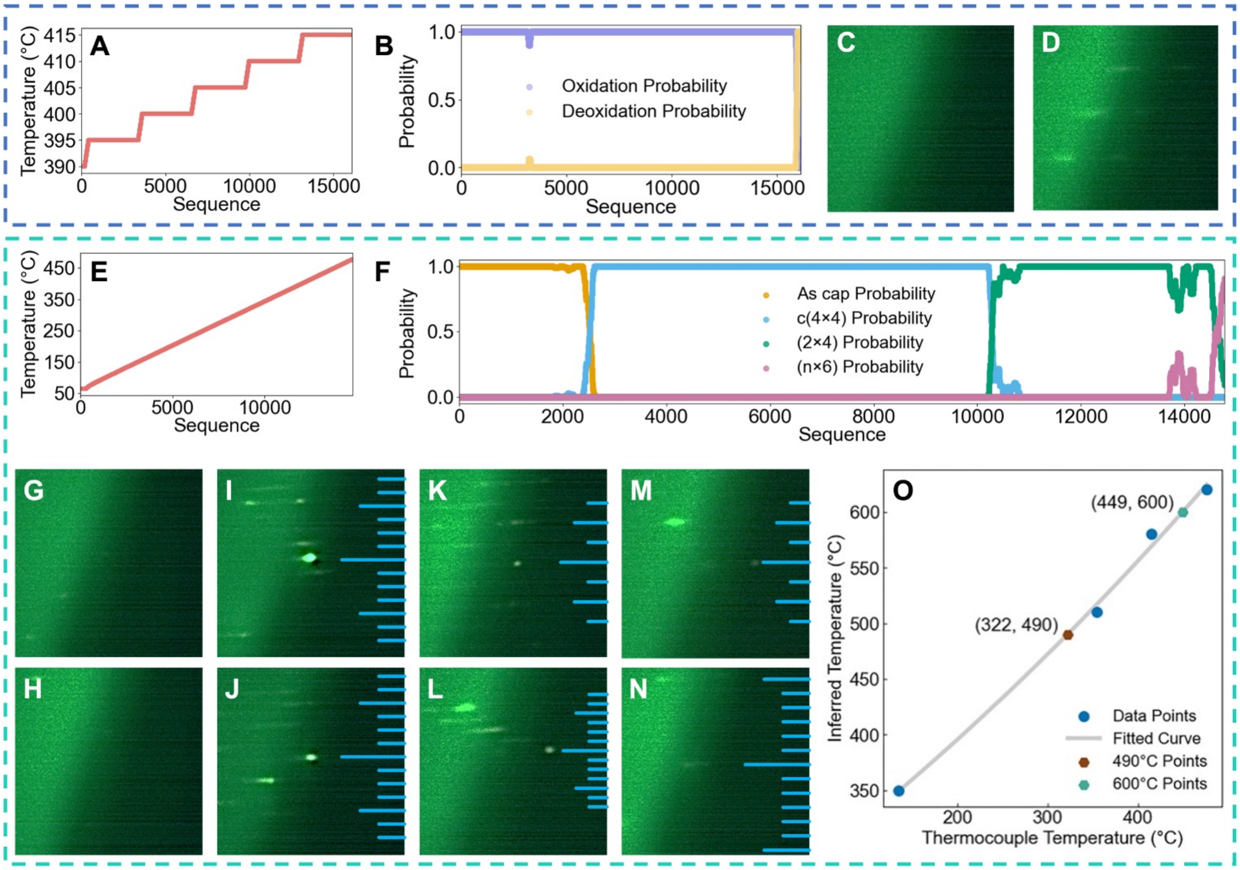

Fig. S14.

The parameter initialization experiment of the reference sample. (A) Substrate temperature, and (B) the running average results of "Initialization Model" output during substrate deoxidation. The RHEED image captured at around (C) 13,000th and (D) 16,000th sequence of (A). (E) Substrate temperature, and (F) the running average results of "Initialization Model" output during parameter initialization. The RHEED image captured at around (G-H) 2,000th, (I-J) 3,000th, (K-L) 11,000th, and (M-N) 15,000th sequence of (E). RHEED images were captured from two angles. (O) Parameter initialization results. Source data are provided as a Source Data file.

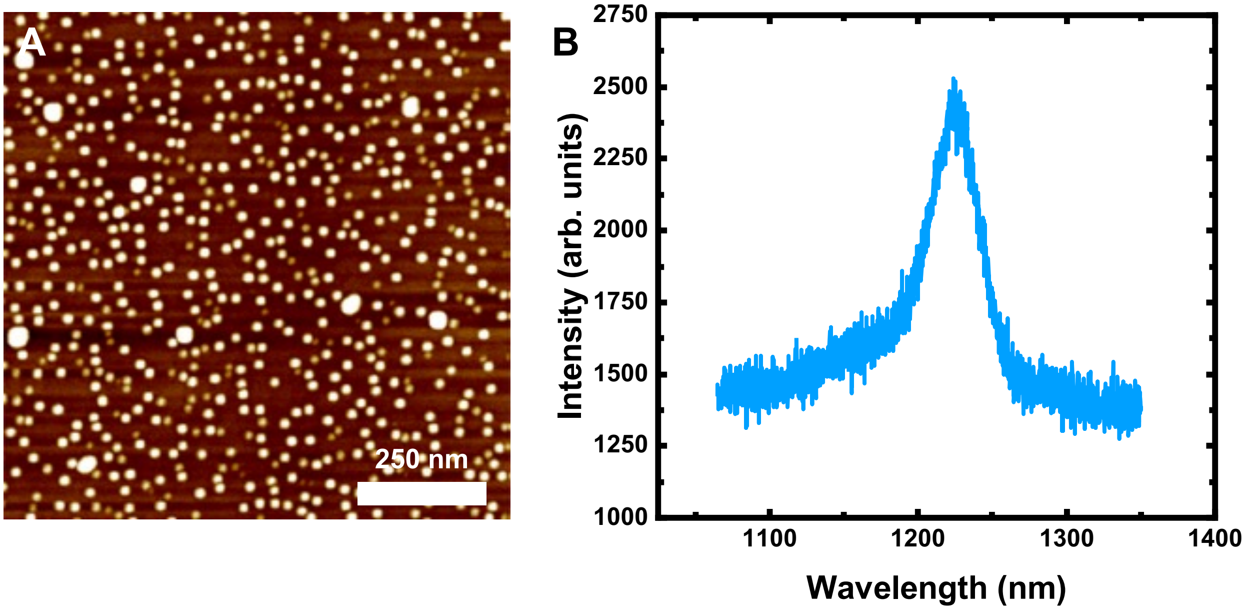

Fig. S15.

The reference sample prepared using parameter initialization. (A) The 1 μm × 1 μm AFM image of the reference sample. (B) Photoluminescence spectrum of QDs.

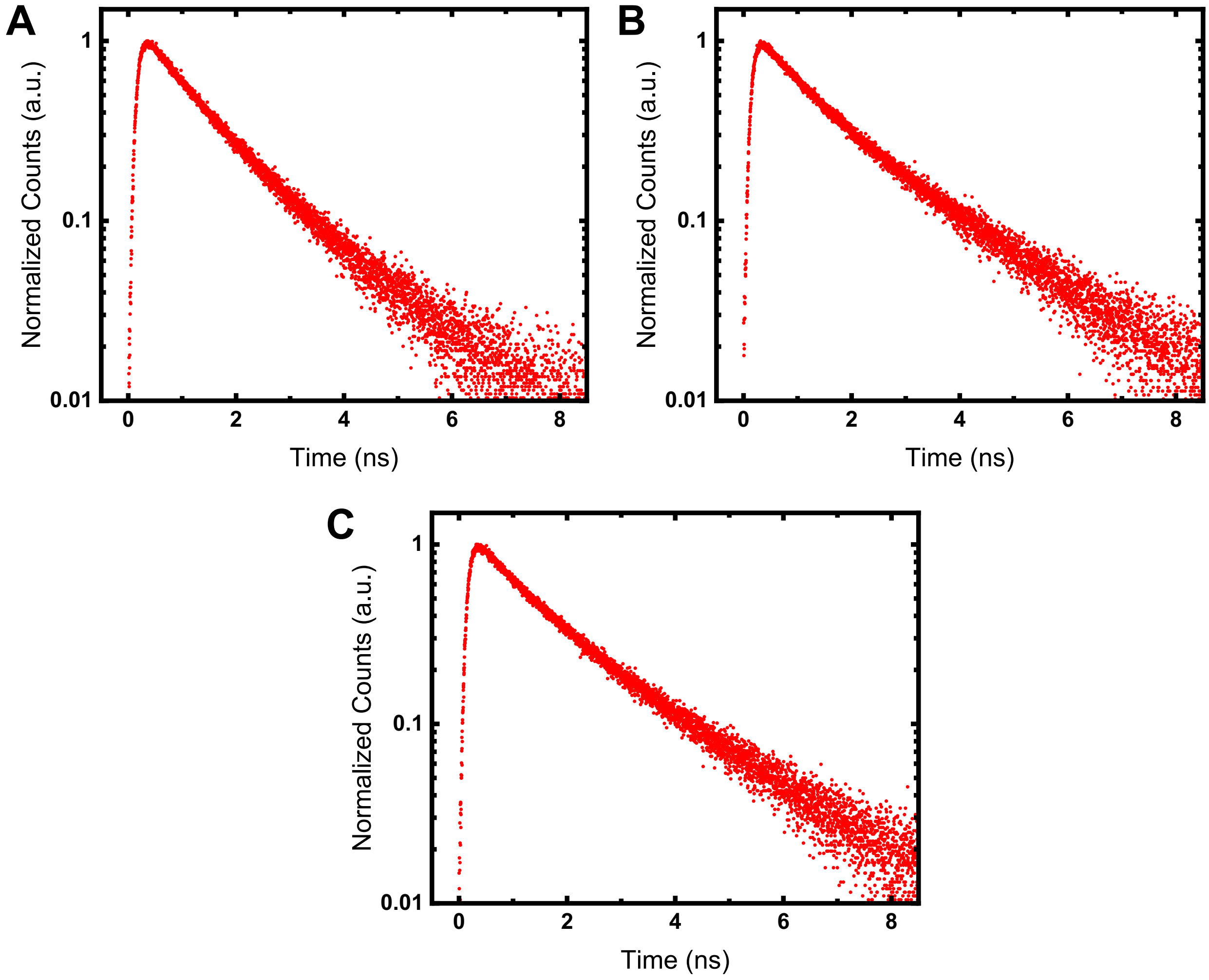

Fig. S16.

Time-resolved PL results. (A) The reference sample prepared using conventional methods, (B) the reference sample prepared using parameter initialization, and (C) the sample prepared using SemiEpi.

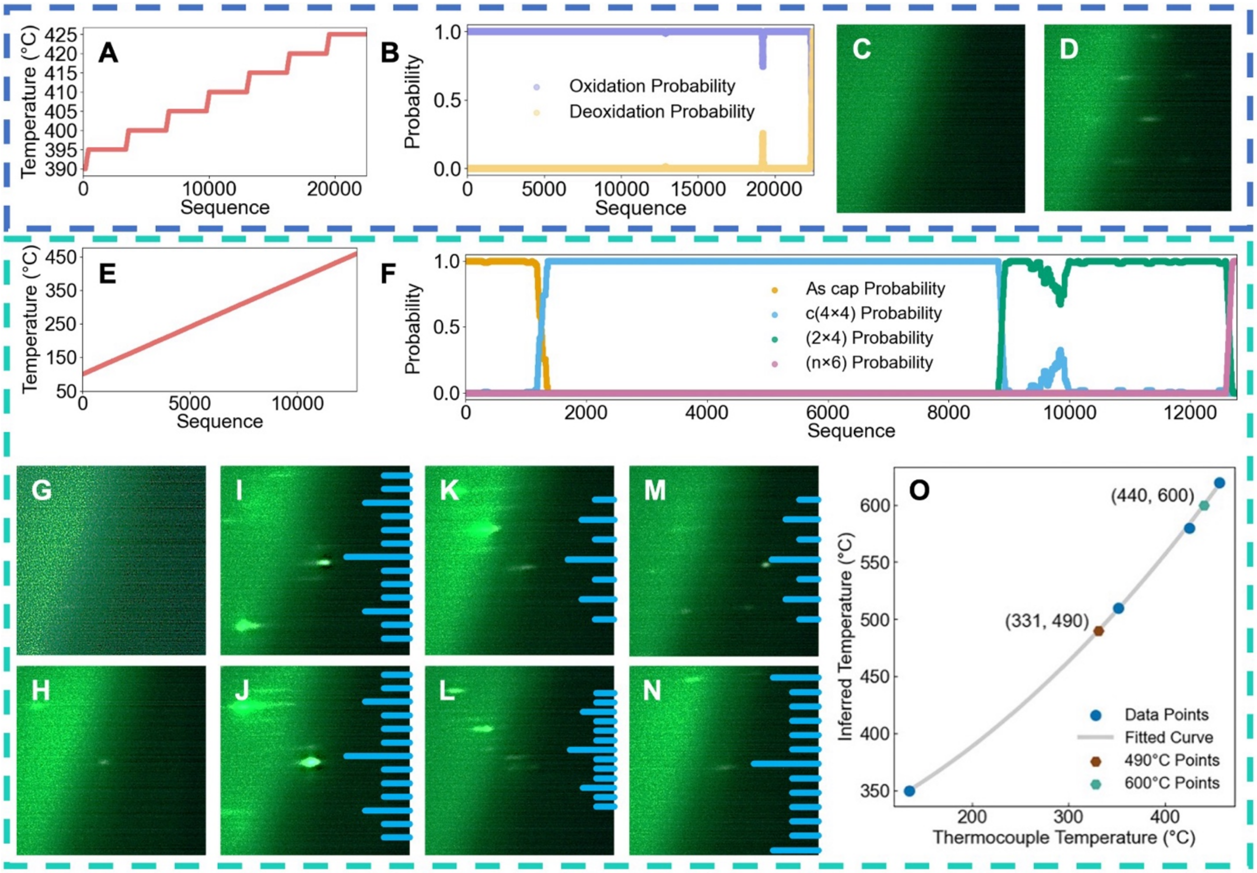

Fig. S17.
The parameter initialization for Sample 1 prepared with SemiEpi. (A) Substrate temperature and (B) the running average results of "Initialization Model" output during substrate deoxidation. The RHEED image captured at around (C) 19,000th and (d) 22,500th sequence of (A). (E) Substrate temperature and (F) the running average results of "Initialization Model" output during parameter initialization. The RHEED image captured at around (G-H) 1,000th, (I-J) 1,200th, (K-L) 8,800th, and (M-N) 12,600th sequence of (E). RHEED images were captured from two angles. The blue lines on the images represent diffraction features: the longest lines mark the specular spots, medium-length lines indicate integer order streaks, and the shortest lines denote half-integer order streaks. (O) Parameter initialization results. Source data are provided as a Source Data file.

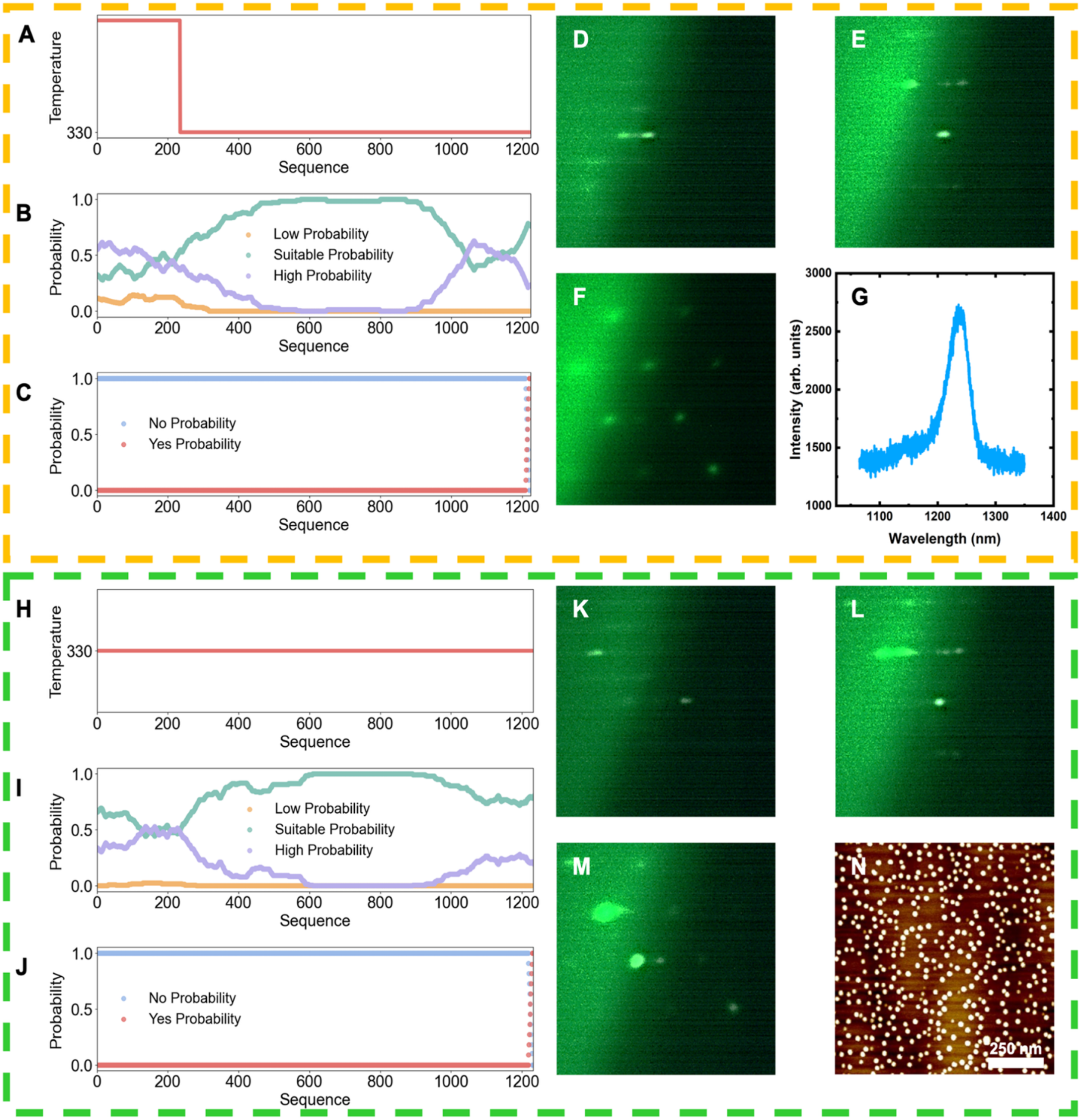

Fig. S18.
The growth of the InAs QDs for Sample 1 prepared with SemiEpi. (A) Substrate temperature, and the running average results of (B) "Temperature Model" and (C) "Shutter Model" output during growth buried InAs QDs. The RHEED image captured at around (d) 100th, (E) 700th, and (f) 1200th sequence of (A). (G) PL spectrum of the buried QDs. (H) Substrate temperature, and the running average results of (I) "Temperature Model" and (J) "Shutter Model" output during growth surface InAs QDs. The RHEED image captured at around (K)100th, (L) 700th, and (M) 1200th sequence of (H). (N) The 1 µm × 1 µm AFM image of the surface QDs. Source data are provided as a Source Data file.

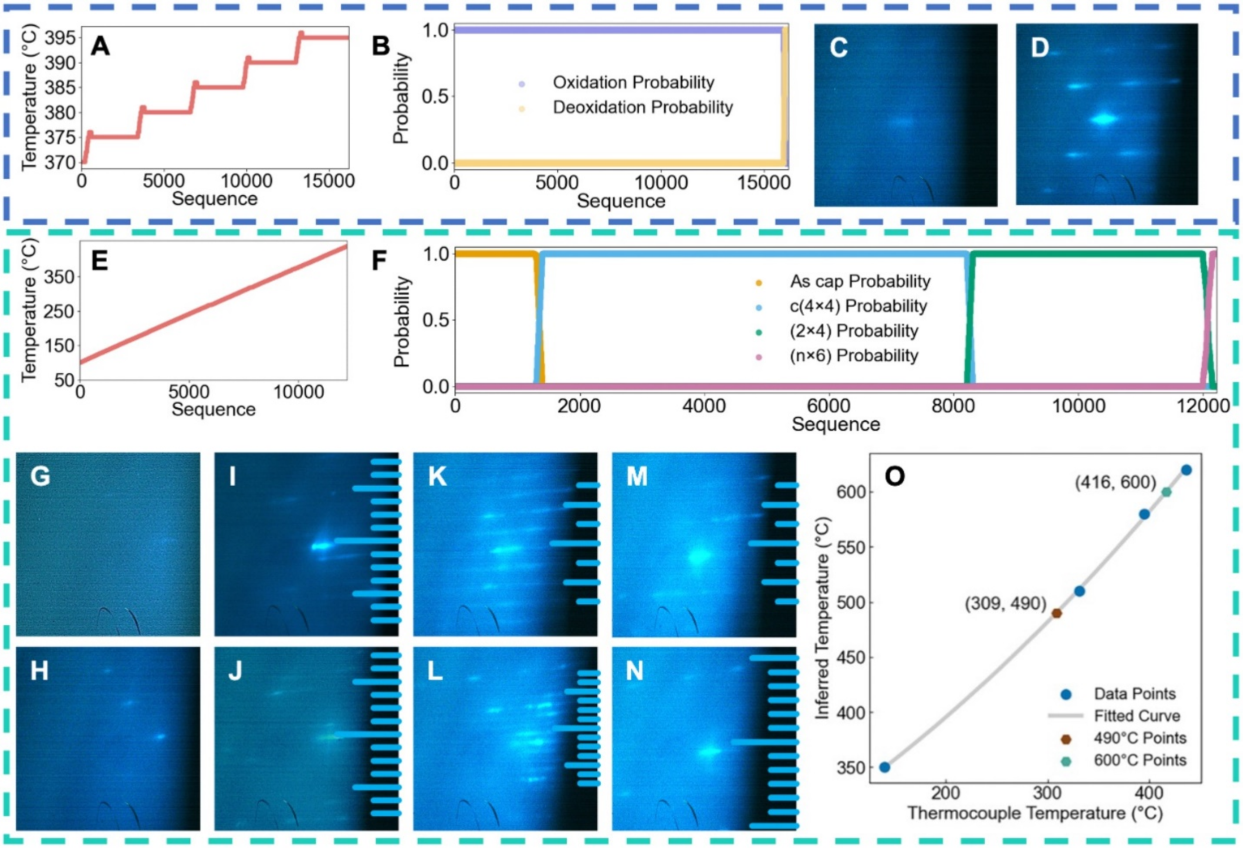

Fig. S19.
The parameter initialization for Sample 2 prepared with SemiEpi. (A) Substrate temperature and (B) the running average results of "Initialization Model" output during substrate deoxidation. The RHEED image captured at around (C) 13,000th and (d) 16,000th sequence of (A). (E) Substrate temperature and (F) the running average results of "Initialization Model" output during parameter initialization. The RHEED image captured at around (G-H) 1,200th, (I-J) 1,400th, (K-L) 8,300th, and (M-N) 12,200th sequence of (E). RHEED images were captured from two angles. The blue lines on the images represent diffraction features: the longest lines mark the specular spots, medium-length lines indicate integer order streaks, and the shortest lines denote half-integer order streaks. (O) Parameter initialization results. Source data are provided as a Source Data file.

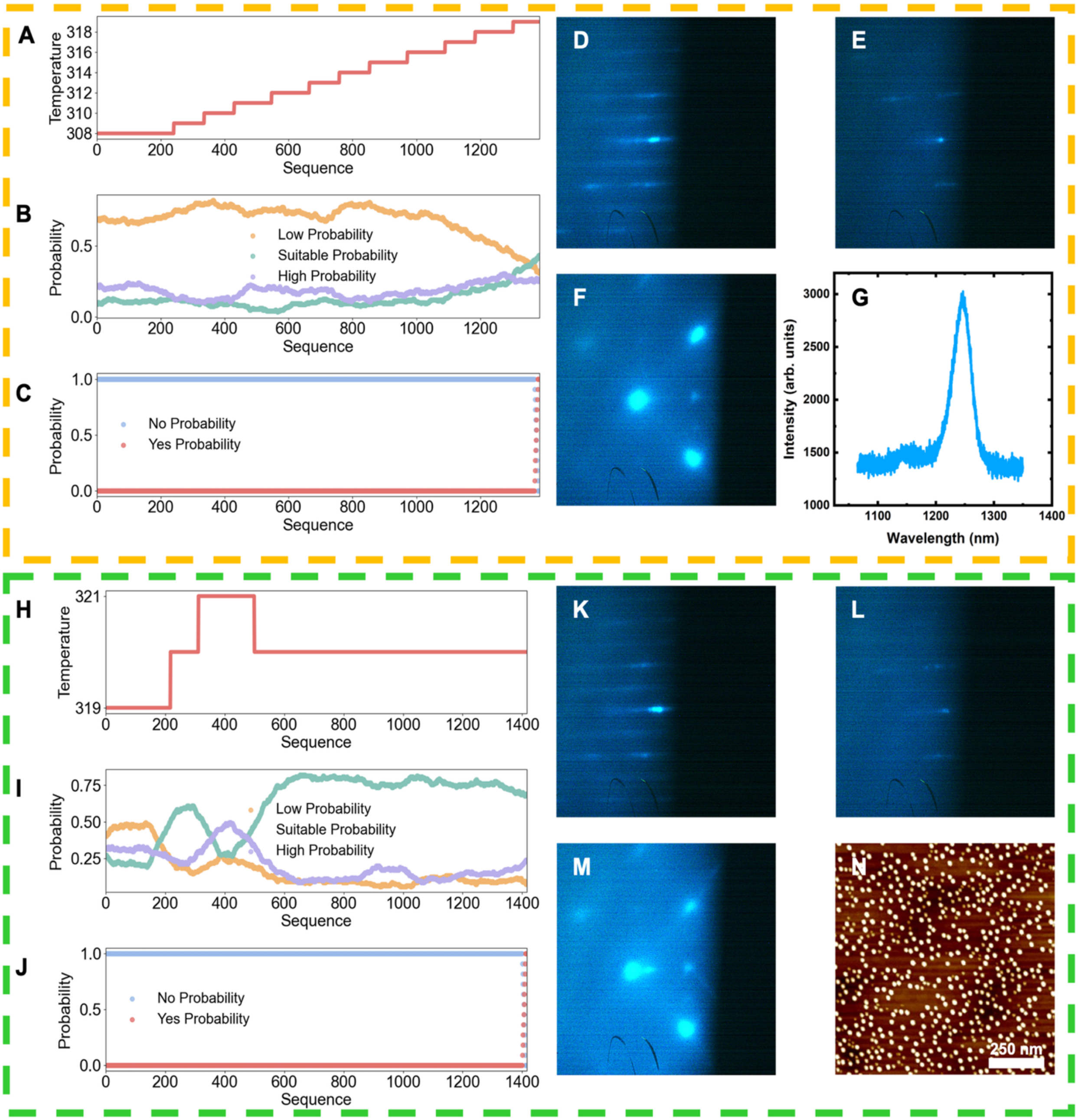

Fig. S20.

The growth of the InAs QDs for Sample 2 prepared with SemiEpi. (A) Substrate temperature, and the running average results of (B) "Temperature Model" and (C) "Shutter Model" output during growth buried InAs QDs. The RHEED image captured at around (D) 100th, (E) 700th, and (F) 1400th sequence of (A). (G) PL spectrum of the buried QDs. (H) Substrate temperature, and the running average results of (I) "Temperature Model" and (J) "Shutter Model" output during growth surface InAs QDs. The RHEED image captured at around (K)100th, (L) 700th, and (M) 1400th sequence of (H). (N) The 1 µm × 1 µm AFM image of the surface QDs. Source data are provided as a Source Data file.

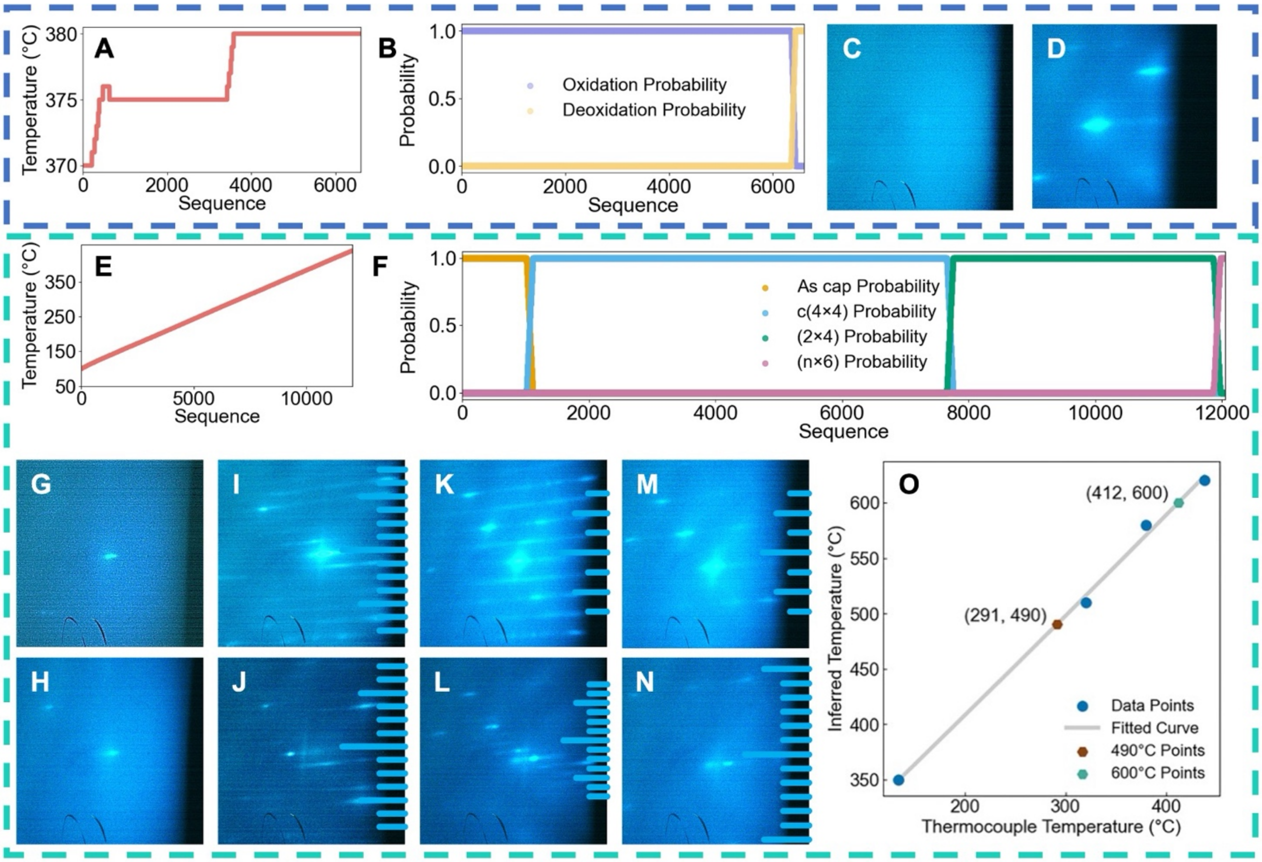

Fig. S21.
The parameter initialization for Sample 3 prepared with SemiEpi. (A) Substrate temperature and (B) the running average results of "Initialization Model" output during substrate deoxidation. The RHEED image captured at around (C) 3,400th and (D) 6,600th sequence of (A). (E) Substrate temperature and (F) the running average results of "Initialization Model" output during parameter initialization. The RHEED image captured at around (G-H) 900th, (I-J) 1,100th, (K-L) 7,800th, and (M-N) 12,000th sequence of (E). RHEED images were captured from two angles. The blue lines on the images represent diffraction features: the longest lines mark the specular spots, medium-length lines indicate integer order streaks, and the shortest lines denote half-integer order streaks. (O) Parameter initialization results. Source data are provided as a Source Data file.

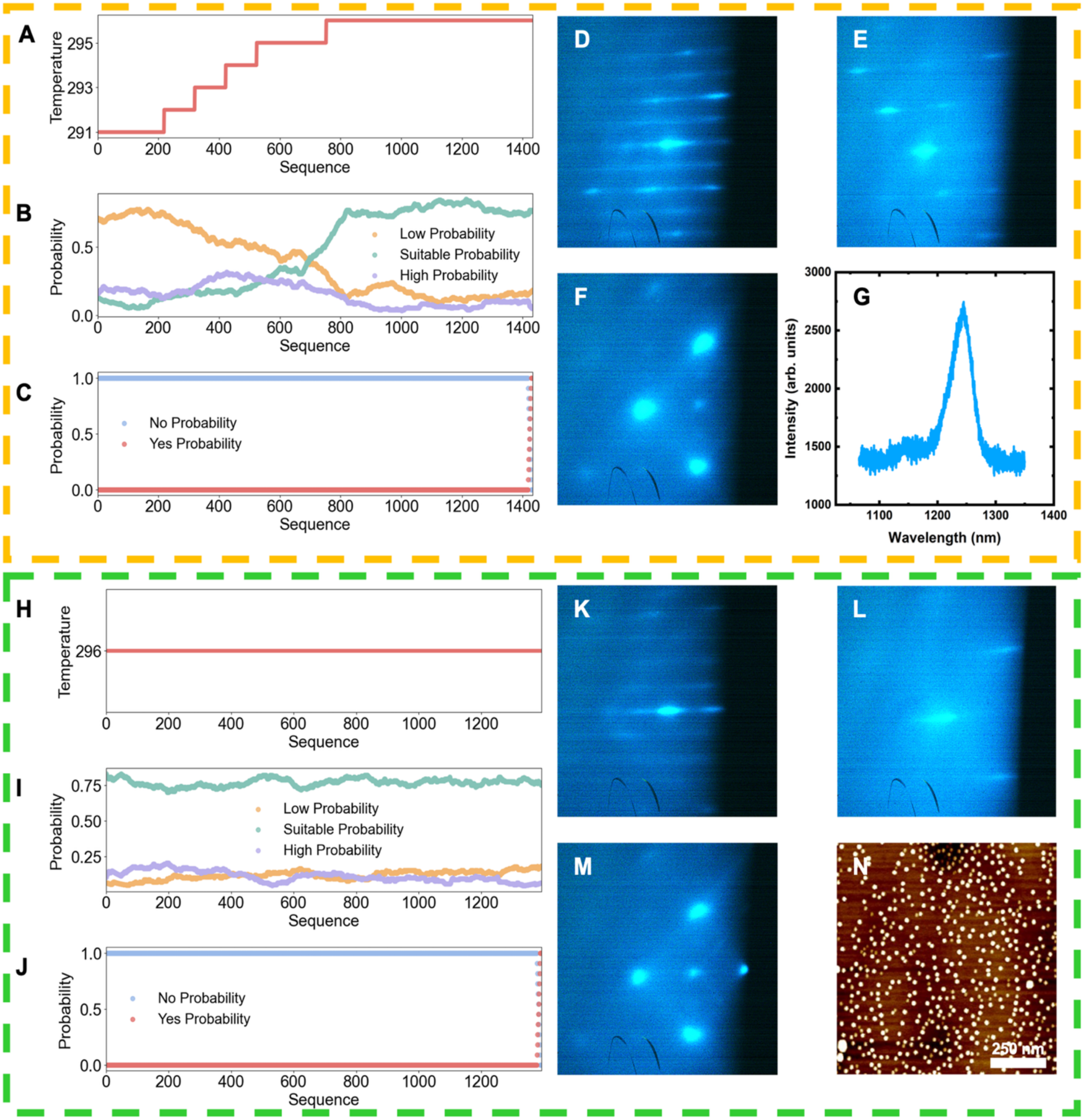

Fig. S22.
The growth of the InAs QDs for Sample 3 prepared with SemiEpi. (A) Substrate temperature, and the running average results of (B) "Temperature Model" and (C) "Shutter Model" output during growth buried InAs QDs. The RHEED image captured at around (D) 100th, (E) 700th, and (F) 1400th sequence of (A). (G) PL spectrum of the buried QDs. (H) Substrate temperature, and the running average results of (I) "Temperature Model" and (J) "Shutter Model" output during growth surface InAs QDs. The RHEED image captured at around (K) 100th, (L) 700th, and (M) 1400th sequence of (H). (N) The 1 μm × 1 μm AFM image of the surface QDs. Source data are provided as a Source Data file.

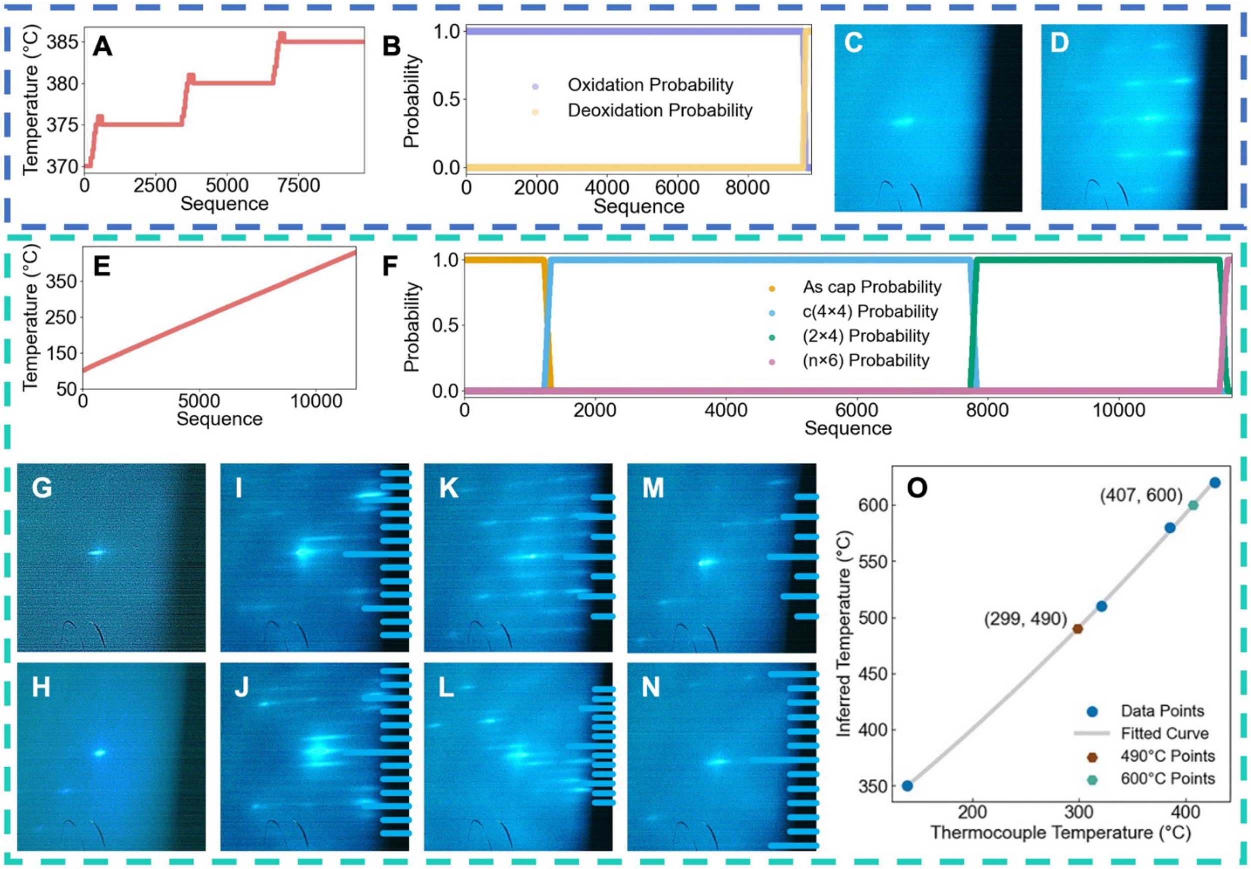

Fig. S23.
The parameter initialization for Sample 4 prepared with SemiEpi. (A) Substrate temperature and (B) the running average results of "Initialization Model" output during substrate deoxidation. The RHEED image captured at around (C) 6,500th and (D) 9,700th sequence of (A). (E) Substrate temperature and (F) the running average results of "Initialization Model" output during parameter initialization. The RHEED image captured at around (G-H) 1,100th, (I-J) 1,300th, (K-L) 8,000th, and (M-N) 11,700th sequence of (E). RHEED images were captured from two angles. The blue lines on the images represent diffraction features: the longest lines mark the specular spots, medium-length lines indicate integer order streaks, and the shortest lines denote half-integer order streaks. (O) Parameter initialization results. Source data are provided as a Source Data file.

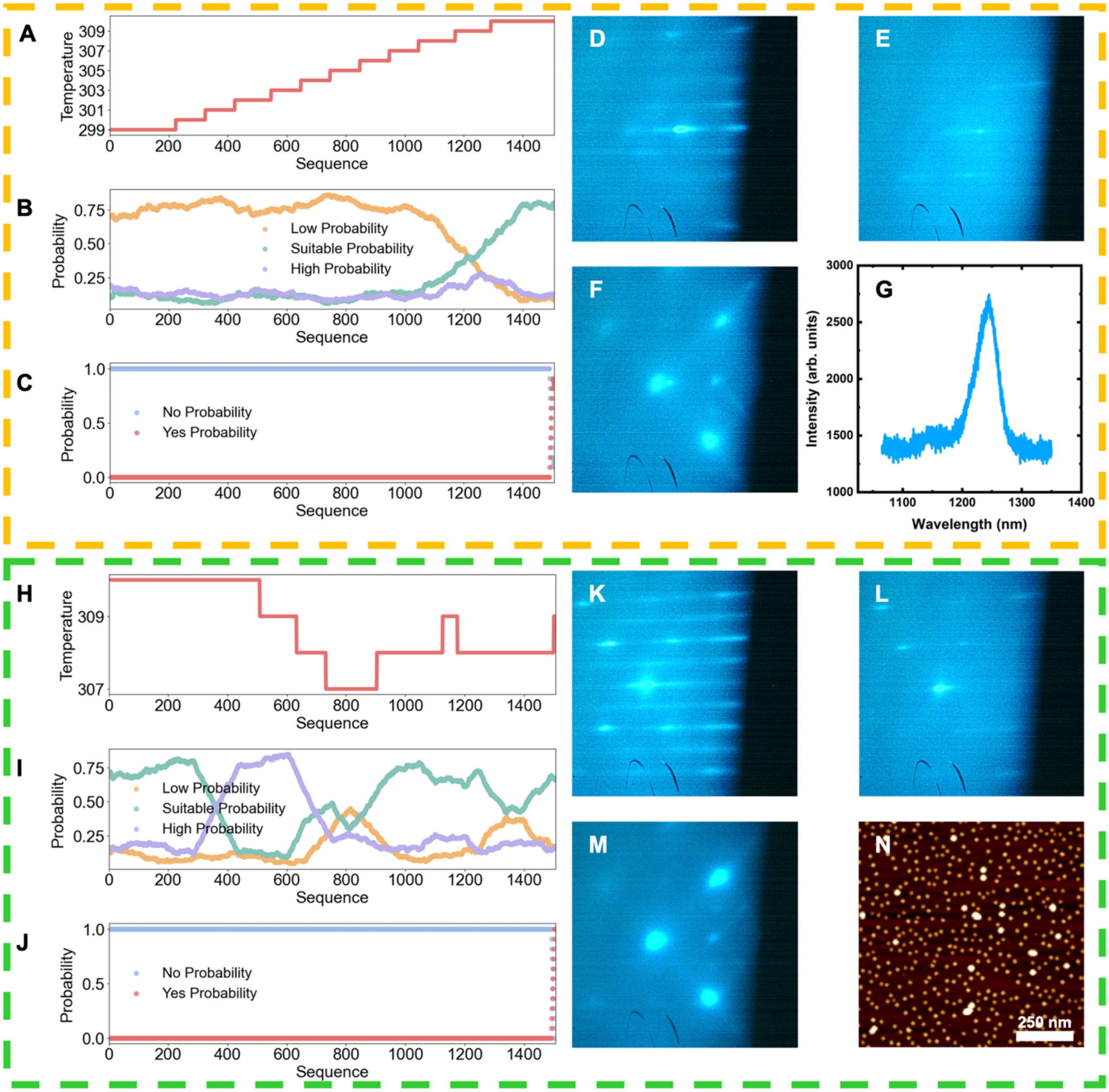

Fig. S24.
The growth of the InAs QDs for Sample 4 prepared with SemiEpi. (A) Substrate temperature, and the running average results of (B) "Temperature Model" and (C) "Shutter Model" output during growth buried InAs QDs. The RHEED image captured at around (D) 100th, (E) 700th, and (F) 1500th sequence of (A). (G) PL spectrum of the buried QDs. (H) Substrate temperature, and the running average results of (I) "Temperature Model" and (J) "Shutter Model" output during growth surface InAs QDs. The RHEED image captured at around (K) 100th, (L) 700th, and (M) 1500th sequence of (H). (N) The 1 μm × 1 μm AFM image of the surface QDs. Source data are provided as a Source Data file.

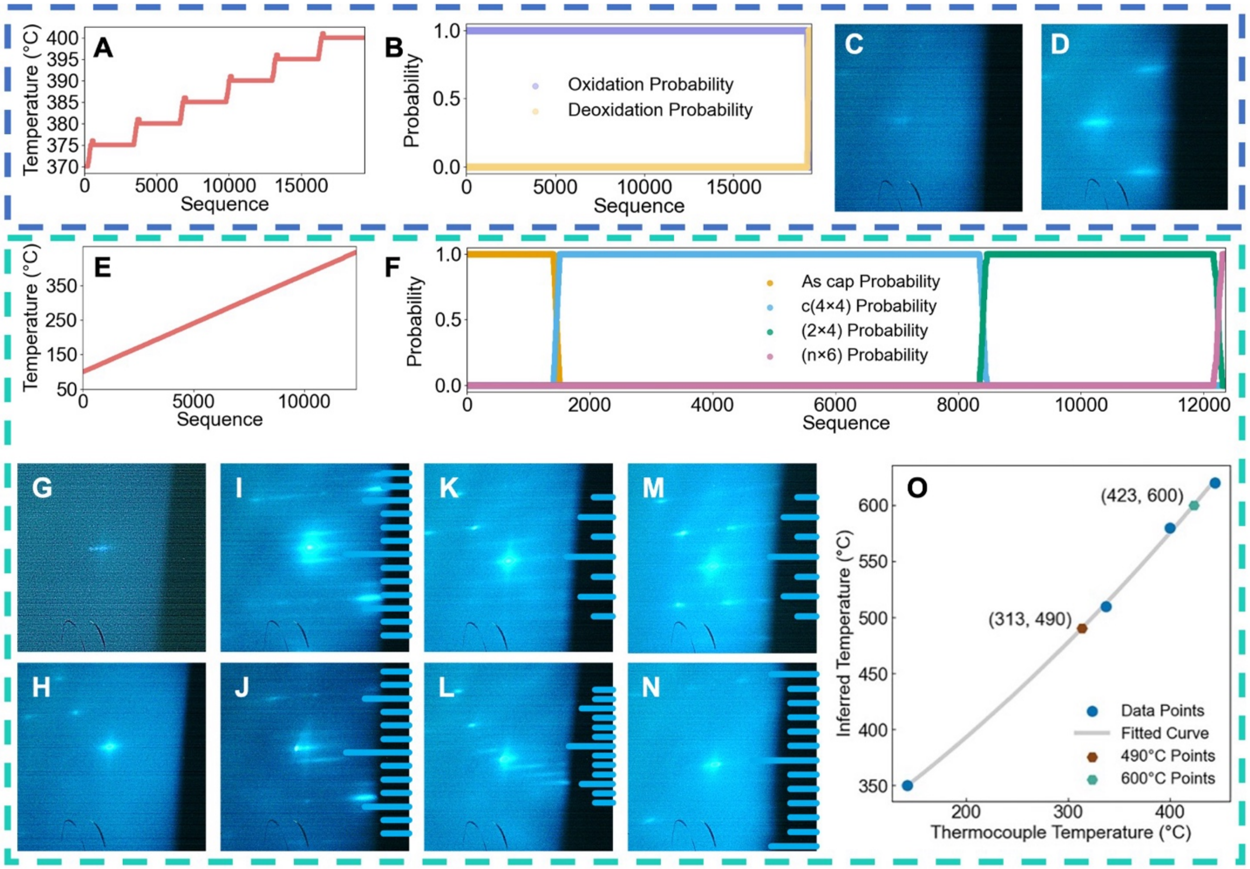

Fig. S25.

The parameter initialization for Sample 5 prepared with SemiEpi. (A) Substrate temperature and (B) the running average results of "Initialization Model" output during substrate deoxidation. The RHEED image captured at around (C) 16,100th and (D) 19,300th sequence of (A). (E) Substrate temperature and (F) the running average results of "Initialization Model" output during parameter initialization. The RHEED image captured at around (G-H) 1,300th, (I-J) 1,500th, (K-L) 8,500th, and (M-N) 12,300th sequence of (E). RHEED images were captured from two angles. The blue lines on the images represent diffraction features: the longest lines mark the specular spots, medium-length lines indicate integer order streaks, and the shortest lines denote half-integer order streaks. (O) Parameter initialization results. Source data are provided as a Source Data file.

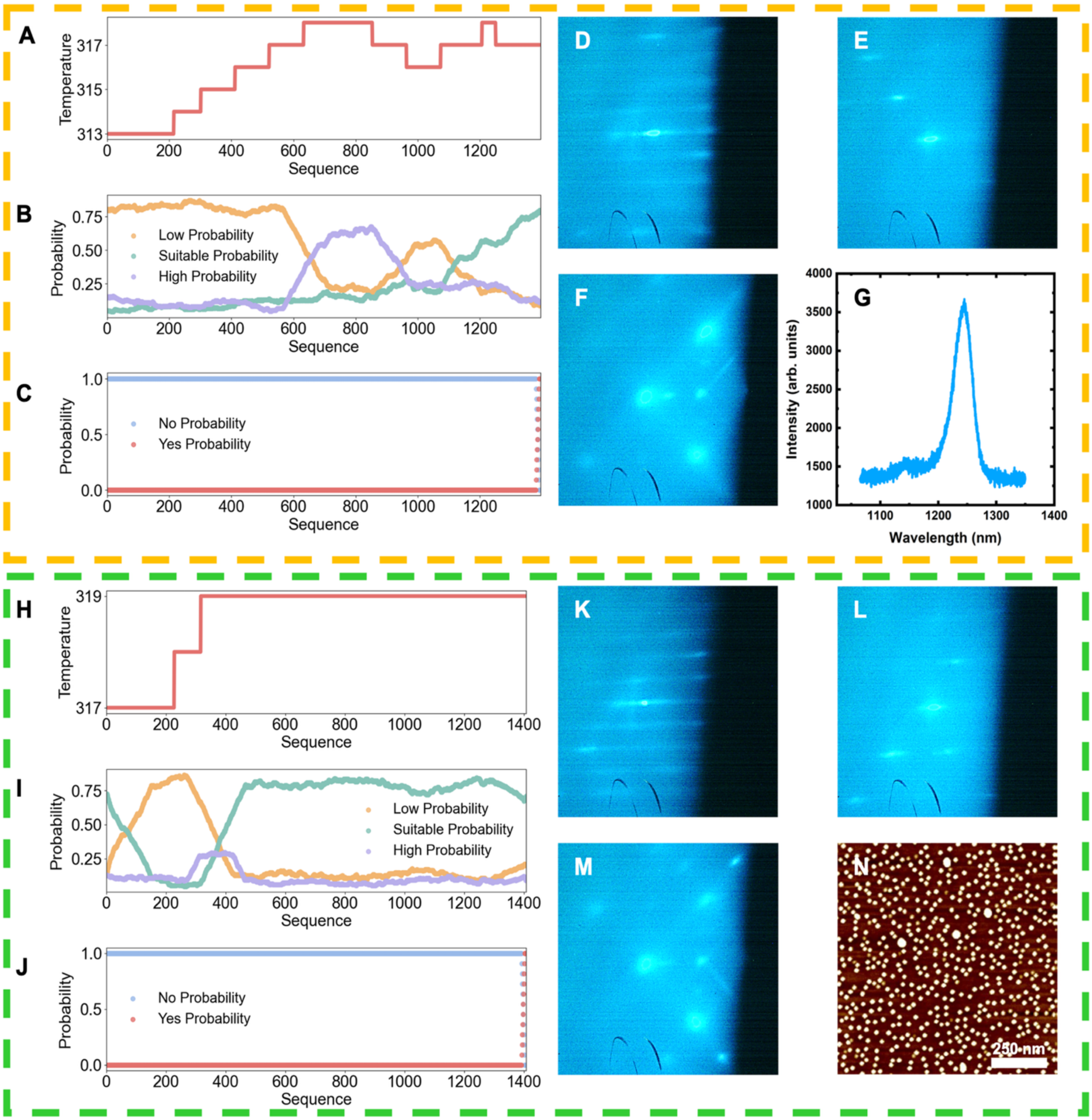

Fig. S26.

The growth of the InAs QDs for Sample 5 prepared with SemiEpi. (A) Substrate temperature, and the running average results of (B) "Temperature Model" and (C) "Shutter Model" output during growth buried InAs QDs. The RHEED image captured at around (D) 100th, (E) 700th, and (F) 1400th sequence of (A). (G) PL spectrum of the buried QDs. (H) Substrate temperature, and the running average results of (I) "Temperature Model" and (J) "Shutter Model" output during growth surface InAs QDs. The RHEED image captured at around (K) 100th, (L) 700th, and (M) 1400th sequence of (H). (N) The 1 μm × 1 μm AFM image of the surface QDs. Source data are provided as a Source Data file.

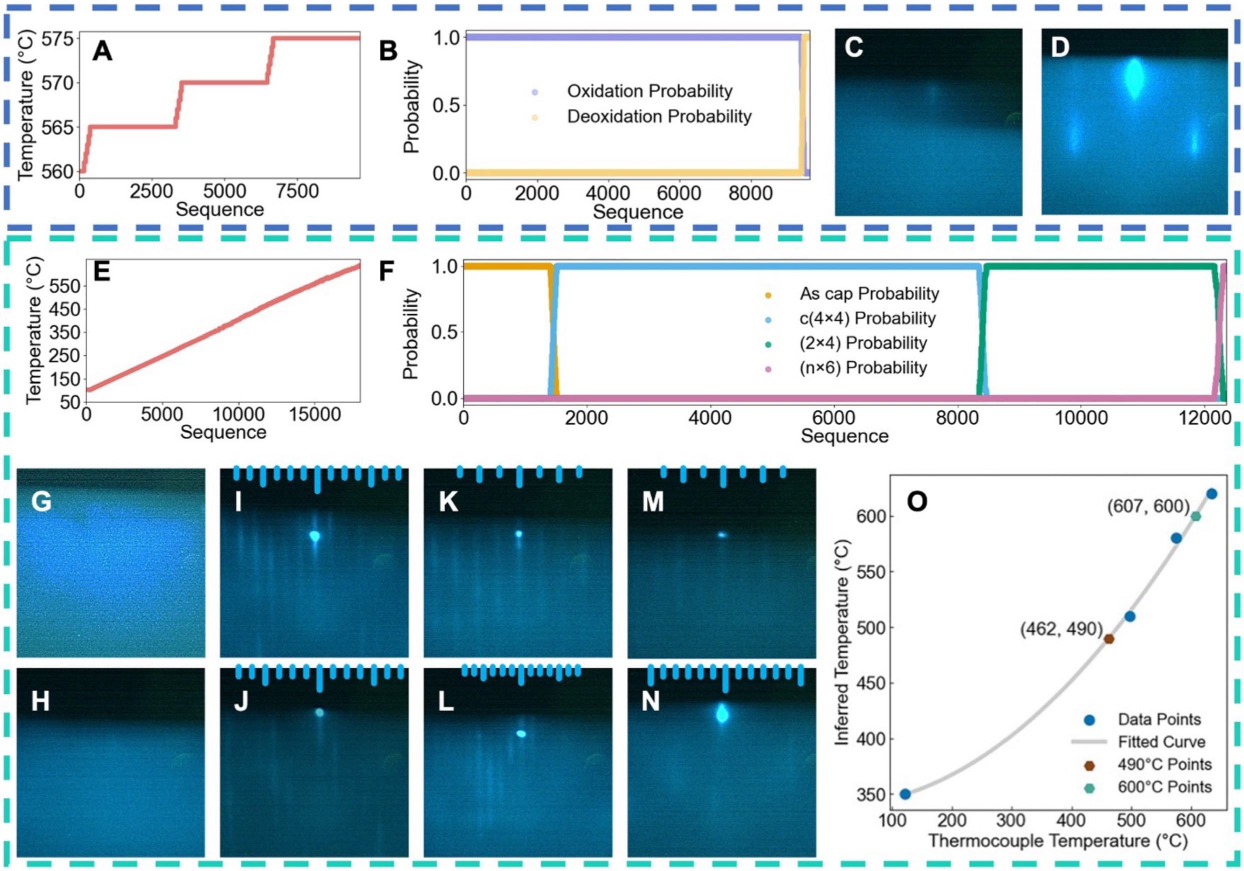

Fig. S27.

The parameter initialization for Sample 6 prepared with SemiEpi on another MBE reactor. (A) Substrate temperature and (B) the running average results of "Initialization Model" output during substrate deoxidation. The RHEED image captured at around (C) 6,200th and (D) 9,500th sequence of (A). (E) Substrate temperature and (F) the running average results of "Initialization Model" output during parameter initialization. The RHEED image captured at around (G-H) 800th, (I-J) 1000th, (K-L) 1,3000th, and (M-N) 17,900th sequence of (E). RHEED images were captured from two angles. The blue lines on the images represent diffraction features: the longest lines mark the specular spots, medium-length lines indicate integer order streaks, and the shortest lines denote half-integer order streaks. (O) Parameter initialization results. Source data are provided as a Source Data file.

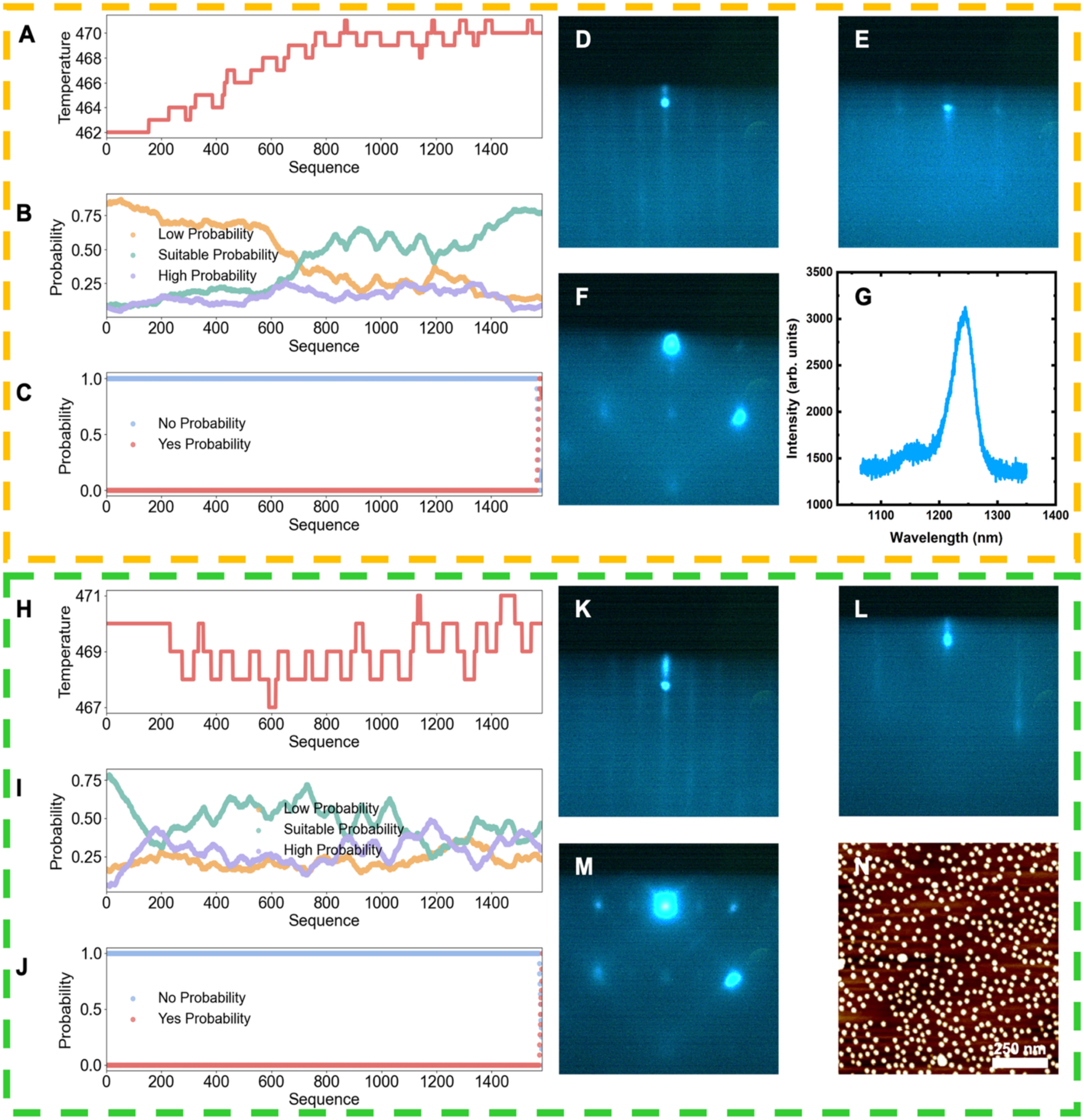

Fig. S28.

The growth of the InAs QDs for Sample 6 prepared with SemiEpi on another MBE reactor. (A) Substrate temperature, and the running average results of (B) "Temperature Model" and (C) "Shutter Model" output during growth buried InAs QDs. The RHEED image captured at around (D) 100th, (E) 800th, and (F) 1600th sequence of (A). (G) PL spectrum of the buried QDs. (H) Substrate temperature, and the running average results of (I) "Temperature Model" and (J) "Shutter Model" output during growth surface InAs QDs. The RHEED image captured at around (K) 100th, (L) 800th, and (M) 1600th sequence of (H). (N) The 1 μm × 1 μm AFM image of the surface QDs. Source data are provided as a Source Data file.

Table S1.
The parameters in the formula.

Symbol	Physical Meaning
m_0	Free electron mass
m_e^*	Electron effective mass
V_{0e}	Electron confinement barrier height
e	Elementary charge
$\hbar$	Reduced Planck constant
m_h^*	Hole effective mass
V_{0h}	Hole confinement barrier height

Table S2.

Growth parameters and characterization results for the training samples.

Temperature (°C)	Density (cm-2)	Intensity (arb. units)	Wavelength (nm)	FWHM (meV)
453	6.25E+10	1629	1225	36.98
458	8.32E+10	2591	1198	52.68
460	1.30E+11	3724	1219	38.85
462	3.24E+10	1575	1205	50.96
464	6.32E+10	1681	1236	42.81
465	1.12E+11	1847	1139	69.81
470	6.96E+10	3415	1215	34.09
470	6.84E+10	3381	1249	33.18
470	7.20E+10	1882	1226	35.99
472	7.32E+10	1812	1173	65.16
477	5.96E+10	2115	1239	35.62
480	5.56E+10	2035	1245	32.23
486	5.80E+10	1980	1231	33.45
486	4.57E+10	1821	1233	32.07
487	5.90E+10	2613	1234	27.30
488	3.88E+10	2627	1260	40.13
490	4.36E+10	2541	1243	29.27
492	4.27E+10	1962	1237	32.59
492	4.25E+10	1925	1235	32.97
493	3.92E+10	3383	1246	29.11
493	2.72E+10	2350	1226	45.05
494	3.70E+10	2250	1209	37.68
495	3.19E+10	2839	1230	38.91
495	1.84E+10	2109	1216	36.14
496	1.28E+10	2077	1213	36.01
497	3.04E+10	1712	1209	33.45
497	1.09E+11	1590	1142	80.61
499	2.32E+10	1876	1190	39.45
501	2.38E+10	2364	1238	34.87
501	2.20E+10	2034	1208	33.22

Movie S1.
The experiment of QD growth.

Movie S2.
The experiment of QD growth with another type of RHEED fluorescent screens.

Movie S3.
The experiment of QD growth on another MBE reactor.

Data S1.
Source data for the plotted results in the main text and Supplementary Materials.

REFERENCES

1. Y. Suematsu, Dynamic Single-Mode Lasers. *J. Light. Technol.* **32**, 1144–1158 (2014).

2. M. Lee, J.-W. Jo, Y.-J. Kim, S. Choi, S. M. Kwon, S. P. Jeon, A. Facchetti, Y.-H. Kim, S. K. Park, Corrugated heterojunction metal-oxide thin-film transistors with high electron mobility via vertical interface manipulation. *Adv Mater.* **30**, 1804120 (2018).

3. J. Kwoen, Y. Arakawa, Multiclass classification of reflection high-energy electron diffraction patterns using deep learning. *J. Cryst. Growth* **593**, 126780 (2022).

4. T. Wu, S. Kheiri, R. J. Hickman, H. Tao, T. C. Wu, Z.-B. Yang, X. Ge, W. Zhang, M. Abolhasani, K. Liu, A. Aspuru-Guzik, E. Kumacheva, Self-driving lab for the photochemical synthesis of plasmonic nanoparticles with targeted structural and optical properties. *Nat. Commun.* **16**, 1473 (2025).

5. A. Slattery, Z. Wen, P. Tenblad, J. Sanjosé-Orduna, D. Pintossi, T. den Hartog, T. Noël, Automated self-optimization, intensification, and scale-up of photocatalysis in flow. *Science* **383**, eadj1817 (2024).

6. K. Choudhary, B. DeCost, C. Chen, A. Jain, F. Tavazza, R. Cohn, C. W. Park, A. Choudhary, A. Agrawal, S. J. L. Billinge, E. Holm, S. P. Ong, C. Wolverton, Recent advances and applications of deep learning methods in materials science. *npj Comput. Mater.* **8**, 59 (2022).

7. G. E. Karniadakis, I. G. Kevrekidis, L. Lu, P. Perdikaris, S. Wang, L. Yang, Physics-informed machine learning. *Nat. Rev. Phys.* **3**, 422–440 (2021).

8. D. Bimberg, M. Grundmann, N. N. Ledentsov, *Quantum Dot Heterostructures*. (John Wiley & Sons, 1999).

9. J. Liu, Y. Nie, W. Xue, L. Wu, H. Jin, G. Jin, Z. Zhai, C. Fu, Size effects on structural and optical properties of tin oxide quantum dots with enhanced quantum confinement. *J. Mater. Res. Technol.* **9**, 8020–8028 (2020).

10. P. B. Joyce, T. J. Krzyzewski, G. R. Bell, T. S. Jones, E. C. Le Ru, R. Murray, Optimizing the growth of 1.3 μm InAs/GaAs quantum dots. *Phys. Rev. B* **64**, 235317 (2001).

11. R. Heitz, I. Mukhametzhanov, A. Madhukar, A. Hoffmann, D. Bimberg, Temperature dependent optical properties of self-organized InAs/GaAs quantum dots. *J. Electron. Mater.* **28**, 520–527 (1999).

12. R. Schlereth, J. Hajer, L. Fürst, S. Schreyeck, H. Buhmann, L. W. Molenkamp, Band edge thermometry for the MBE growth of (Hg,Cd)Te-based materials. *J. Cryst. Growth* **537**, 125602 (2020).

13. M. F. Vilela, G. K. Pribil, K. R. Olsson, D. D. Lofgreen, HgCdTe molecular beam epitaxy growth temperature calibration using spectroscopic ellipsometry. *J. Electron. Mater.* **41**, 2937–2942 (2012).

14. S. E. Aleksandrov, G. A. Gavrilov, A. A. Kapralov, G. Y. Sotnikova, D. F. Chernykh, A. N. Alekseev, A. L. Dudin, I. V. Kogan, A. P. Shkurko, Pyrometer unit for GaAs substrate temperature control in an MBE system. *Tech. Phys.* **49**, 123–127 (2004).

15. G. R. Bell, J. G. Belk, C. F. McConville, T. S. Jones, Species intermixing and phase transitions on the reconstructed (001) surfaces of GaAs and InAs. *Phys. Rev. B* **59**, 2947–2955 (1999).

16. C. Shen, W. Zhan, K. Xin, M. Li, Z. Sun, H. Cong, C. Xu, J. Tang, Z. Wu, B. Xu, Z. Wei, C. Xue, C. Zhao, Z. Wang, Machine-learning-assisted and real-time-feedback-controlled growth of InAs/GaAs quantum dots. *Nat. Commun.* **15**, 2724 (2024).

17. C. Shen, W. Zhan, S. Pan, H. Hao, N. Zhuo, K. Xin, H. Cong, C. Xu, B. Xu, T. K. Ng, S. Chen, C. Xue, Z. Wang, C. Zhao, Real-time self-optimization of quantum dot laser emissions during machine learning-assisted epitaxy. *Adv. Mater* **12**, 2503059 (2025).

18. C. Shen, W. Zhan, J. Tang, Z. Wu, B. Xu, C. Zhao, Z. Wang, Universal deoxidation of semiconductor substrates assisted by machine learning and real-time feedback control. *ACS Appl. Mater. Interfaces* **16**, 18213–18221 (2024).

19. S. Tao, M. Zhang, Z. Zhao, H. Li, R. Ma, Y. Che, X. Sun, L. Su, C. Sun, X. Chen, H. Chang, S. Zhou, Z. Li, H. Lin, Y. Liu, W. Yu, Z. Xu, H. Hao, S. Moura, X. Zhang, Y. Li, X. Hu, G. Zhou, Non-destructive degradation pattern decoupling for early battery trajectory prediction via physics-informed learning. *Energy Environ. Sci.* **18**, 1544–1559 (2025).

20. P. Nikolaev, D. Hooper, F. Webber, R. Rao, K. Decker, M. Krein, J. Poleski, R. Barto, B. Maruyama, Autonomy in materials research: A case study in carbon nanotube growth. *npj Comput. Mater.* **2**, 16031 (2016).

21. B. P. MacLeod, F. G. L. Parlane, T. D. Morrissey, F. Häse, L. M. Roch, K. E. Dettelbach, R. Moreira, L. P. E. Yunker, M. B. Rooney, J. R. Deeth, V. Lai, G. J. Ng, H. Situ, R. H. Zhang, M. S. Elliott, T. H. Haley, D. J. Dvorak, A. Aspuru-Guzik, J. E. Hein, C. P. Berlinguette, Self-driving laboratory for accelerated discovery of thin-film materials. *Sci. Adv.* **6**, eaaz8867 (2020).

22. R. Shimizu, S. Kobayashi, Y. Watanabe, Y. Ando, T. Hitosugi, Autonomous materials synthesis by machine learning and robotics. *APL Mater.* **8**, 111110 (2020).

23. A. A. Volk, R. W. Epps, D. T. Yonemoto, B. S. Masters, F. N. Castellano, K. G. Reyes, M. Abolhasani, AlphaFlow: Autonomous discovery and optimization of multi-step chemistry using a self-driven fluidic lab guided by reinforcement learning. *Nat. Commun.* **14**, 1403 (2023).

24. S. B. Harris, A. Biswas, S. J. Yun, K. M. Roccapriore, C. M. Rouleau, A. A. Puretzky, R. K. Vasudevan, D. B. Geohegan, K. Xiao, Autonomous synthesis of thin film materials with pulsed laser deposition enabled by in situ spectroscopy and automation. *Small Methods* **8**, 2301763 (2024).

25. D. M. Fébba, K. R. Talley, K. Johnson, S. Schaefer, S. R. Bauers, J. S. Mangum, R. W. Smaha, A. Zakutayev, Autonomous sputter synthesis of thin film nitrides with composition controlled by Bayesian optimization of optical plasma emission. *APL Mater.* **11**, 071119 (2023).

26. J. W. Lee, D. Schuh, M. Bichler, G. Abstreiter, Size and density estimation of self-assembled InAs quantum dots on GaAs(001) substrate through the analysis of RHEED patterns. *Phys. Status Solidi C* **0**, 1121–1124 (2003).

27. A. Freundlich, C. Rajapaksha, M. Gunasekera, in *37th IEEE Photovoltaic Specialists Conference* (2011), pp. 003483–003485.

28. C. F. Schuck, R. A. McCown, A. Hush, A. Mello, S. Roy, J. W. Spinuzzi, B. Liang, D. L. Huffaker, P. J. Simmonds, Self-assembly of (111)-oriented tensile-strained quantum dots by molecular beam epitaxy. *J. Vac. Sci. Technol. B* **36**, 031803 (2018).

29. H. Z. Song, T. Usuki, Y. Nakata, N. Yokoyama, H. Sasakura, S. Muto, Formation of InAs/GaAs quantum dots from a subcritical InAs wetting layer: A reflection high-energy electron diffraction and theoretical study. *Phys. Rev. B* **73**, 115327 (2006).

30. M. Rei Vilar, J. El Beghdadi, F. Debontridder, R. Artzi, R. Naaman, A. M. Ferraria, A. M. Botelho do Rego, Characterization of wet-etched GaAs (100) surfaces. *Surf. Interface Anal.* **37**, 673–682 (2005).

31. A. Ohtake, Surface reconstructions on GaAs(001). *Surf. Sci. Rep.* **63**, 295–327 (2008).

32. I. Karpov, N. Venkateswaran, G. Bratina, W. Gladfelter, A. Franciosi, L. Sorba, Arsenic cap layer desorption and the formation of GaAs(001)c(4×4) surfaces. *J. Vac. Sci. Technol. B. Microelectron.* **13**, 2041–2048 (1995).

33. T. T. Chiang, W. E. Spicer, Arsenic on GaAs: Fermi-level pinning and thermal desorption studies. *J. Vac. Sci. Technol. C* **7**, 724–730 (1989).

34. R. W. Bernstein, A. Borg, H. Husby, B. O. Fimland, J. K. Grepstad, Capping and decapping of MBE grown GaAs(001), $Al_{0.5}Ga_{0.5}As$(001), and AlAs(001) investigated with ASP, PES, LEED, and RHEED. *Appl. Surf. Sci.* **56**, 74–80 (1992).

35. U. Resch, N. Esser, Y. S. Raptis, W. Richter, J. Wasserfall, A. Förster, D. I. Westwood, Arsenic passivation of MBE grown GaAs(100): Structural and electronic properties of the decapped surfaces. *Surf. Sci.* **269**, 797–803 (1992).

36. A. Ohtake, M. Ozeki, T. Yasuda, T. Hanada, Atomic structure of the GaAs(001)-(2×4) surface under As flux. *Phys. Rev. B* **65**, 165315 (2002).

37. A. Ohtake, Structure and composition of Ga-rich (6×6) reconstructions on GaAs(001). *Phys. Rev. B* **75**, 153302 (2007).

38. T. Ritari, J. Tuominen, H. Ludvigsen, J. Petersen, T. Sørensen, T. P. Hansen, H. R. Simonsen, Gas sensing using air-guiding photonic bandgap fibers. *Opt. Exp.* **12**, 4080–4087 (2004).

39. S. Kabi, A. G. U. Perera, Effect of quantum dot size and size distribution on the intersublevel transitions and absorption coefficients of III-V semiconductor quantum dot. *J. Appl. Phys.* **117**, 124303 (2015).

40. C. Y. Ngo, S. F. Yoon, W. J. Fan, S. J. Chua, Effects of size and shape on electronic states of quantum dots. *Phys. Rev. B* **74**, 245331 (2006).

41. L. Aderras, E. Feddi, A. Bah, F. Dujardin, C. A. Duque, On the electronic states in lens-shaped quantum dots. *Phys. Status Solidi C* **254**, 1700144 (2017).

42. Y. Berdnikov, P. Holewa, S. Kadkhodazadeh, J. M. Śmigiel, A. Sakanas, A. Frackowiak, K. Yvind, M. Syperek, E. Semenova, Near-critical Stranski-Krastanov growth of InAs/InP quantum dots. *Sci. Rep.* **14**, 23697 (2024).

43. N. Bart, C. Dangel, P. Zajac, N. Spitzer, J. Ritzmann, M. Schmidt, H. G. Babin, R. Schott, S. R. Valentin, S. Scholz, Y. Wang, R. Uppu, D. Najer, M. C. Löbl, N. Tomm, A. Javadi, N. O. Antoniadis, L. Midolo, K. Müller, R. J. Warburton, P. Lodahl, A. D. Wieck, J. J. Finley, A. Ludwig, Wafer-scale epitaxial modulation of quantum dot density. *Nat. Commun.* **13**, 1633 (2022).

44. Y. Nohara, K. Matsumoto, H. Soejima, N. Nakashima, Explanation of machine learning models using shapley additive explanation and application for real data in hospital. *Comput. Methods Programs Biomed.* **214**, 106584 (2022).

45. J. T. Hancock, T. M. Khoshgoftaar, CatBoost for big data: An interdisciplinary review. *J. Big Data* **7**, 94 (2020).

46. A. Y. Cho, Growth of III–V semiconductors by molecular beam epitaxy and their properties. *Thin Solid Films* **100**, 291–317 (1983).

47. U. Resch, S. M. Scholz, U. Rossow, A. B. Müller, W. Richter, A. Förster, Thermal desorption of amorphous arsenic caps from GaAs(100) monitored by reflection anisotropy spectroscopy. *Appl. Surf. Sci.* **63**, 106–110 (1993).

48. Y. Qi, Z. Yang, W. Sun, M. Lou, J. Lian, W. Zhao, X. Deng, Y. Ma, A comprehensive overview of image enhancement techniques. *Arch. Comput. Methods Eng.* **29**, 583–607 (2022).

49. H. J. Kim, M. Chong, T. G. Rhee, Y. G. Khim, M.-H. Jung, Y.-M. Kim, H. Y. Jeong, B. K. Choi, Y. J. Chang, Machine-learning-assisted analysis of transition metal dichalcogenide thin-film growth. *Nano Converg.* **10**, 10 (2023).

50. W. Xu, F. Gao, J. Zhang, X. Tao, A. Alkhateeb, Deep learning based channel covariance matrix estimation with user location and scene images. *IEEE Trans. Commun.* **69**, 8145–8158 (2021).

51. N. C. F. Codella, Q. B. Nguyen, S. Pankanti, D. A. Gutman, B. Helba, A. C. Halpern, J. R. Smith, Deep learning ensembles for melanoma recognition in dermoscopy images. *IBM J. Res. Dev.* **61**, 5:1–5:15 (2017).

52. U. Ruby, V. Yendapalli, Binary cross entropy with deep learning technique for image classification. *Int. J. Adv. Trends Comput. Sci. Eng* **9**, 5393–5397 (2020).

53. C. P. Roca, O. T. Burton, J. Neumann, S. Tareen, C. E. Whyte, V. Gergelits, R. V. Veiga, S. Humblet-Baron, A. Liston, A cross entropy test allows quantitative statistical comparison of t-SNE and UMAP representations. *Cell Rep. Methods* **3**, 100390 (2023).

54. C. van Zyl, X. Ye, R. Naidoo, Harnessing eXplainable artificial intelligence for feature selection in time series energy forecasting: A comparative analysis of Grad-CAM and SHAP. *Appl. Energy* **353**, 122079 (2024).

55. S.-K. Park, J. Tatebayashi, Y. Arakawa, Formation of ultrahigh-density InAs/AlAs quantum dots by metalorganic chemical vapor deposition. *Appl. Phys. Lett.* **84**, 1877–1879 (2004).

56. Y. Ruan, J. Li, Q. Xiao, Y. Wu, M. Shi, High-temperature failure evolution analysis of k-type film thermocouples. *Micromachines* **14**, 2070 (2023).

57. V. A. Drebushchak, Thermocouples, their characteristic temperatures, and simple approximation of the emf vs. T. *Thermochim. Acta* **603**, 218–226 (2015).

58. L. Chu, M. Arzberger, G. Böhm, G. Abstreiter, Influence of growth conditions on the photoluminescence of self-assembled InAs/GaAs quantum dots. *J. Appl. Phys.* **85**, 2355–2362 (1999).

59. M.-Y. Kong, X.-L. Wang, D. Pan, Y.-P. Zeng, J. Wang, W. Ge, A comparison of photoluminescence properties of InGaAs/GaAs quantum dots with a single quantum well. *J. Appl. Phys.* **86**, 1456–1459 (1999).

60. D. Lu, J. Ahn, S. Freisem, D. Gazula, D. G. Deppe, Lens-shaped all-epitaxial quantum dot microcavity. *Appl. Phys. Lett.* **87**, 163105 (2005).

61. Y. Li, Z. Song, Z. Li, G. Sun, C. S. Tan, W. Fan, Q. J. Wang, Theoretical design of mid-infrared interband cascade lasers in SiGeSn system. *N. J. Phys.* **22**, 083061 (2020).

62. A. Maltsi, T. Niermann, T. Streckenbach, K. Tabelow, T. Koprucki, Numerical simulation of TEM images for In(Ga)As/GaAs quantum dots with various shapes. *Opt. Quant. Electron.* **52**, 257 (2020).

63. J. K. Kim, T. A. Strand, R. L. Naone, L. A. Coldren, Design parameters for lateral carrier confinement in quantum-dot lasers. *Appl. Phys. Lett.* **74**, 2752–2754 (1999).

64. J. Shumway, A. J. Williamson, A. Zunger, A. Passaseo, M. DeGiorgi, R. Cingolani, M. Catalano, P. Crozier, Electronic structure consequences of In/Ga composition variations in self-assembled $In_xGa_{1-x}As$/GaAs alloy quantum dots. *Phys. Rev. B* **64**, 125302 (2001).

65. A. N. Kosarev, V. V. Chaldyshev, Carrier localization by a quantum dot in a quantum well. *Phys. Rev. Appl.* **16**, 044046 (2021).

66. U. B. Singh, D. Singh, S. Kumar, R. Dhar, M. B. Pandey, The optical properties of quantum dots in anisotropic media. *J. Mol. Liq.* **241**, 1009–1012 (2017).

67. S. Ramanathan, G. Petersen, K. Wijesundara, R. Thota, E. A. Stinaff, M. L. Kerfoot, M. Scheibner, A. S. Bracker, D. Gammon, Quantum-confined Stark effects in coupled InAs/GaAs quantum dots. *Appl. Phys. Lett.* **102**, 213101 (2013).

68. H. Ye, P. Lu, Z. Yu, B. Jia, H. Feng, Y. Liu, Equilibrium critical size of coherent InSb/GaSb quantum dot. *Phys. E* **42**, 2402–2405 (2010).

69. N. Baer, S. Schulz, P. Gartner, S. Schumacher, G. Czycholl, F. Jahnke, Influence of symmetry and Coulomb correlation effects on the optical properties of nitride quantum dots. *Phys. Rev. B* **76**, 075310 (2007).

70. X. Zhang, P. Sharma, Size dependency of strain in arbitrary shaped anisotropic embedded quantum dots due to nonlocal dispersive effects. *Phys. Rev. B* **72**, 195345 (2005).

71. G. Bastard, *Wave mechanics applied to semiconductor heterostructures*. (1990).

72. D. L. Aronstein, C. R. Stroud Jr., General series solution for finite square-well energy levels for use in wave-packet studies. *Am. J. Phys.* **68**, 943–949 (2000).

73. A. L. Efros, L. E. Brus, Nanocrystal quantum dots: From discovery to modern development. *ACS Nano* **15**, 6192–6210 (2021).

74. M. Wang, C. Li, F. Ke, Recurrent multi-level residual and global attention network for single image deraining. *Neural Comput. Appl.* **35**, 3697–3708 (2023).

75. S. Cheng, R. Chan, A. Du, CACFTNet: A hybrid cov-attention and cross-layer fusion transformer network for hyperspectral image classification. *IEEE Trans. Geosci. Remote Sens.* **62**, 1–17 (2024).

76. D. Hong, Z. Han, J. Yao, L. Gao, B. Zhang, A. Plaza, J. Chanussot, SpectralFormer: Rethinking hyperspectral image classification with transformers. *IEEE Trans. Geosci. Remote Sens.* **60**, 1–15 (2022).

77. D. Kohen, S. Bao, K. H. Lee, K. E. K. Lee, C. S. Tan, S. F. Yoon, E. A. Fitzgerald, The role of AsH3 partial pressure on anti-phase boundary in GaAs-on-Ge grown by MOCVD–Application to a 200mm GaAs virtual substrate. *J. Cryst. Growth* **421**, 58–65 (2015).

78. S. Lazzari, M. Abolhasani, K. F. Jensen, Modeling of the formation kinetics and size distribution evolution of II–VI quantum dots. *React. Chem. Eng.* **2**, 567–576 (2017).
